# Electrical recordings from dendritic spines of adult mouse hippocampus and effect of the actin cytoskeleton

**DOI:** 10.3389/fnmol.2022.769725

**Published:** 2022-08-25

**Authors:** Avner Priel, Xiao-Qing Dai, Xing-Zhen Chen, Noelia Scarinci, María del Rocío Cantero, Horacio F. Cantiello

**Affiliations:** ^1^The Mina & Everard Goodman Faculty of Life Sciences, Bar-Ilan University, Ramat-Gan, Israel; ^2^Department of Pharmacology, Alberta Diabetes Institute, University of Alberta, Edmonton, AB, Canada; ^3^Department of Physiology, University of Alberta, Edmonton, AB, Canada; ^4^Laboratorio de Canales Iónicos, Instituto Multidisciplinario de Salud, Tecnología y Desarrollo, Consejo Nacional de Investigaciones Científicas y Técnicas de Argentina (CONICET) - Universidad Nacional de Santiago del Estero (UNSE), Santiago del Estero, Argentina

**Keywords:** hippocampus, hippocampal neurons, dendritic spines, NMDA receptor, synapse, electrical oscillations, patch-clamping

## Abstract

Dendritic spines (DS) are tiny protrusions implicated in excitatory postsynaptic responses in the CNS. To achieve their function, DS concentrate a high density of ion channels and dynamic actin networks in a tiny specialized compartment. However, to date there is no direct information on DS ionic conductances. Here, we used several experimental techniques to obtain direct electrical information from DS of the adult mouse hippocampus. First, we optimized a method to isolate DS from the dissected hippocampus. Second, we used the lipid bilayer membrane (BLM) reconstitution and patch clamping techniques and obtained heretofore unavailable electrical phenotypes on ion channels present in the DS membrane. Third, we also patch clamped DS directly in cultured adult mouse hippocampal neurons, to validate the electrical information observed with the isolated preparation. Electron microscopy and immunochemistry of PDS-95 and NMDA receptors and intrinsic actin networks confirmed the enrichment of the isolated DS preparation, showing open and closed DS, and multi-headed DS. The preparation was used to identify single channel activities and “whole-DS” electrical conductance. We identified NMDA and Ca^2+^-dependent intrinsic electrical activity in isolated DS and *in situ* DS of cultured adult mouse hippocampal neurons. *In situ* recordings in the presence of local NMDA, showed that individual DS intrinsic electrical activity often back-propagated to the dendrite from which it sprouted. The DS electrical oscillations were modulated by changes in actin cytoskeleton dynamics by addition of the F-actin disrupter agent, cytochalasin D, and exogenous actin-binding proteins. The data indicate that DS are elaborate excitable electrical devices, whose activity is a functional interplay between ion channels and the underlying actin networks. The data argue in favor of the active contribution of individual DS to the electrical activity of neurons at the level of both the membrane conductance and cytoskeletal signaling.

## Introduction

Dendritic spines (DS) are small protrusions that stud the surface of neuronal dendrites and represent the postsynaptic connection of most excitatory synapses in the CNS (Ramón y Cajal, 1891; Gray, 1959; Harris and Kater, 1994; Harris, 1999). DS are unique in that they are localized condensations of ion channels and receptors with an underlying network of actin filaments. DS are highly dynamic structures that actively move and are characterized by their morphological diversity thought to provide an anatomical substrate for memory storage and synaptic transmission (Muller et al., 2000; Leuner and Shors, 2004). Within the hippocampus, spines vary greatly in size and shape (Engert and Bonhoeffer, 1999; Bourne and Harris, 2008; von Bohlen Und Halbach, 2009). DS appear in different shapes and forms based on the relative sizes of the spine head and neck (Peters and Kaiserman-Abramof, 1970), including mushroom spines with a large head and a narrow neck, thin spines with a smaller head and a narrow neck, and stubby spines without constriction between the head and the attachment to the shaft. Other types include the so-called filopodium type, with hair-like morphology (Skoff and Hamburger, 1974; Lohmann and Bonhoeffer, 2008), and branched DS in the hippocampus, characterized by having multiple heads (Sorra et al., 1998). Thus, the morphological plasticity of DS reflects a continuum rather than separated classes (Pchitskaya and Bezprozvanny, 2020). In this variety, the role of the DS neck has been the focus of attention and controversy. The length of the neck is associated with the electrical insulation of the DS from the parental dendrite (Perkel and Perkel, 1985; Araya et al., 2014), and compartmentalizing Ca^2+^ (Gamble and Koch, 1987) that underlies input-specific synaptic plasticity. However, electrochromic voltage-sensitive dye labeling showed that the DS may not be electrically isolated from the dendrite to any meaningful extent (Popovic et al., 2014). The electrical function of the DS has largely remained an open question (Tønnesen and Nägerl, 2016).

Spine shape and function are intimately associated with actin cytoskeleton organization (Fifkova and Delay, 1982; Halpain et al., 1998; Fischer et al., 2000; Matus, 2000; Star et al., 2002; Hotulainen and Hoogenraad, 2010), which is quite distinct from the dendritic shaft and other parts of the neuron (Fifkova and Delay, 1982; Oertner and Matus, 2005; Bonilla-Quintana et al., 2020). DS express several actin-associated proteins, including adducin (Matsuoka et al., 1998), α-actinin that binds to NMDA receptors (NMDAR) (Wyszynski et al., 1997), and profilin, which modulates DS function (Ackermann and Matus, 2003). DS-specific actin-binding proteins such as drebrins may mediate plastic responses (Shim and Lubec, 2002). There is a rich interplay between Ca^2+^ signals, and Ca^2+^ dependent proteins, offering further cytoskeletal regulation in DS (Matsuoka et al., 1998; Agassandian et al., 2000). Gelsolin, a Ca^2+^-dependent F-actin severing protein, affects DS actin stability (Star et al., 2002) and regulates NMDA receptors (Furukawa et al., 1997).

Various types of ion channels are present in DS, including voltage-gated Na^+^, K^+^, and Ca^2+^ channels subunits (Mills et al., 1994; Alonso and Widmer, 1997; Drake et al., 1997; Caldwell et al., 2000), acid-sensitive channels, TRP channels (Toth et al., 2005; Zha et al., 2006), and ligand-gated channels, such as glutamate and GABA receptors (Nusser et al., 1998) that provide a broad spectrum of electrical and regulatory functions not presently defined. Direct electrical information on ionic conductance in DS remains largely lacking despite copious literature on their structure and function. Because of their size and dynamic behavior DS have been refractory to conventional electrophysiological approaches. Although no electrical recordings of DS membrane have heretofore been reported, several optical methods have been successfully applied (Palmer and Stuart, 2009) to assess Na^+^ (Rose and Konnerth, 2001) and Ca^2+^ (Holthoff et al., 2002; Araya et al., 2014) transport, and voltage properties of DS were directly obtained with nanopipettes and electroporation by Jayant et al. (2017). Ca^2+^ imaging of DS has confirmed the presence of functional NMDA and AMPA receptors (Yuste et al., 1999; Kovalchuk et al., 2000), and more recently optical voltage methods have provided direct voltage recordings of DS (Popovic et al., 2015; Jayant et al., 2017; Cartailler et al., 2018). Current models of DS function implicate either an active membrane (Perkel and Perkel, 1985; Shepherd et al., 1985; Tsay and Yuste, 2004), or a passive biochemical compartments that control local Ca^2+^ transients required for signaling events associated with long-term potentiation (LTP) (Lynch et al., 1983; Malenka et al., 1988).

Here, we isolated DS from adult mouse hippocampi that were used for bilayer membrane (BLM) reconstitution and patch clamping studies. We identified NMDA and Ca^2+^-dependent cation-selective ion channels and observed DS-generated autonomous electrical activity. Electrical recordings were also obtained by patch clamping of DS *in situ* in cultured hippocampal neurons, which elicited back-propagating dendritic electrical oscillations. The NMDA-induced electrical activity of DS was profoundly modulated by changes in the actin cytoskeleton elicited by the F-actin disrupter cytochalasin D, and actin-binding proteins. The data indicate that DS function as dynamic non-linear filters, with interplay between ligand-activated ion channels and intrinsic actin networks.

## Materials and methods

### Isolation of mouse hippocampi

**FIGURE 1:**
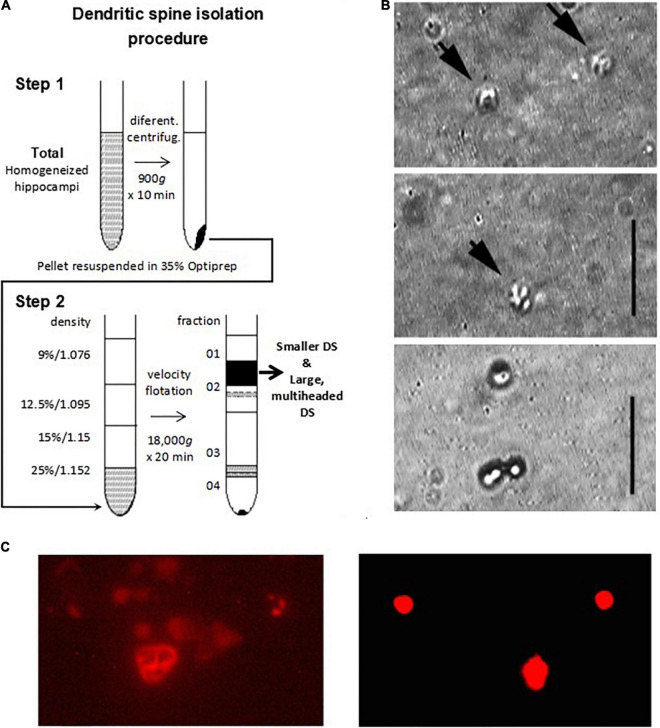
Isolation and imaging of adult mouse hippocampus dendritic spines. **(A)** Isolation procedure of DS developed in the present study. Adapted from Kiebler et al. (1999). **(B)** Isolated DS could be identified by DIC (x100) (panels 1 and 2 from Top to Bottom, vertical line 5 μm), and recognized as “wrinkled sac” shape, of approximately 1 μm diameter, as compared with 1 μm beads (Bottom panel). (**C**, Left) TRITC-phalloidin labeling of fresh (not-fixed) DS allowed rapid identification of abundant F-actin inside DS under fluorescence. (Right) 1 μm diameter fluorescent beads are shown for size comparison.

To isolate mouse hippocampal dendritic spines, we adapted the method previously described by Kiebler et al. (1999) to obtain rat mossy fiber hippocampal DS, with several modifications. Briefly, young adult 5–7-week-old C57BL/6 male mice (Charles River, Laboratories Inc., Wilmington, MA, United States) brains were dissected. The brains were washed with ice-cold saline solution HB, containing 300 mM sucrose, 15 mM Na^+^, N-Tris-(hydroxymethyl-2-aminoetahnesulfonic acid, Na^+^-TES), adjusted at pH 7.4, and 1 mM MgSO_4_ (Terrian et al., 1988). In this condition, mouse hippocampi were obtained. Single DS were isolated from hippocampi following a method previously reported (Kiebler et al., 1999), with modifications, as outlined in Figure 1A.

### Isolation and visualization of adult mouse hippocampal dendritic spines

**FIGURE 2:**
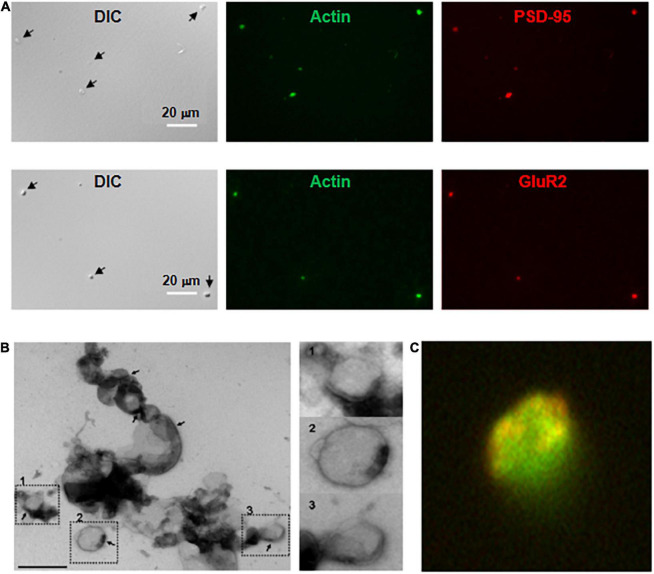
Imaging and immunocytochemical analysis of isolated DS. (**A**, Top) Isolated DS were identified by DIC (×60) and recognized by their “wrinkled sac” shape, of approximately 1 μm diameter. Labeling of fresh (not-fixed) DS allowed identification of abundant intra-DS F-actin (FITC-phalloidin, Green) and PSD-95 (Cy3, Red). (Bottom) Similar approach as in (Top), but co-localization of glutamate receptor GluR2 (Cy3, Red) and FITC-phalloidin to identify F-actin (Green) (×60). **(B)** Negative staining of freshly isolated DS labeled with phosphotungstic acid. Large clusters of individual DS were often identified. However, it is expected that clustering may be a consequence of negative staining. In particular, PSD were observed in several membranes (marked in numbers), with a clear intravesicular content, as expected from DS and not synaptosomes. Right, higher magnification imaging shows DS of different sizes. **(C)** DS with the shape features of the membrane and abundant filamentous material depicting to the open left, identified as polymerized actin (Green). Isolated DS often showed clearly defined PSD (×50,000).

Briefly, mouse hippocampi (*n* = 8) were dissected and immediately washed with HB solution. The isolation procedure was conducted three separate times. Hippocampal tissue was manually homogenized and stored in HB (including Mg^2 +^ to stabilize large membrane structures) using a glass-Teflon Dounce-type homogenizer with a clearance of 0.15 mm. The material was centrifuged at 900*g* with a Sorvall SS34 rotor for 10 min at 4°C without filtering through nylon filters to remove larger aggregates and blood vessels, as indicated (Kiebler et al., 1999). The resulting pellet was washed, resedimented, and resuspended in 0.25 volume of HB, adjusted to 25% Optiprep (Gibco BRL, Life Technologies GmbH, Eggenstein, Germany). The step gradient was conducted by loading 8 ml of 25% Optiprep/pellet solution in an SW28 ultraclear centrifuge tube containing 9, 12.5, 15, and 25% Optiprep solution in HB (8 ml/each). The tube was spun in an SW28 Beckman ultracentrifuge at 18,000*g* for 20 min. A typical gradient (Figure 1A) yielded two bands (O1–O3) as well as a pellet (O4). Bands O1–O2 were not distinguishable as in Kiebler et al. (1999) for the rat material, although it is likely that the amount of initial tissue played a role in the thickness of the bands observed. Bands O1–O2 were removed with a pipette and visualized and further tested by immunocytochemistry and electrophysiological techniques. The preparation contained abundant DS, which were individually revealed with DIC optics (Figure 1B) and after phalloidin labeling (Figure 1C). Little contamination was observed from either the plasma membrane or ER, as disclosed by PSD-95 labeling (Figure 2A, Top).

### Dissociation and culture of hippocampal neurons

C57Bl mice of 5–7 weeks old were killed by cervical dislocation and decapitated according to IACUC guidelines. Hippocampi were dissected out into ice-cold Ca^2+^-free medium Hibernate A (BrainBits, Springfield, IL, United States), and minced into small pieces. Flushing a few times through a fire-polished Pasteur pipette further dispersed tissue. Undispersed pieces were allowed to settle by gravity for 1 min, while the supernatant containing the dissociated hippocampal neurons was transferred to a new tube, which was centrifuged at 200*g* for 1 min. The cell pellet was resuspended in NbActive4 medium (BrainBitz, Springfield, IL, United States) and seeded onto poly-L-lysine-coated glass coverslips. Hippocampal cells were incubated at 37°C, in a wet incubator gassed with 5% CO_2_, 20% O_2_. Hippocampal neurons were kept alive for up to 2 weeks with NbActive4 medium changes every 5 days.

### Immunocytochemistry

Cultured hippocampal cells were fixed for 15 min in freshly prepared para-formaldehyde (4%) and sucrose (2%) in phosphate buffer saline (PBS). Cells were rinsed (×3) with PBS and blocked with BSA (1%) for 30 min prior to exposure to primary antibodies, which are a mouse anti-PSD-95 (1:400) or a mouse anti-GluR2 (1:400) antibody (Neuromab, Davis, CA, United States). After incubation for 1 h in primary antibodies, cells were incubated in a 1:200 donkey anti-mouse Cy3-conjugated secondary antibody (Santa Cruz Biotechnology, Santa Cruz, CA, United States). The cells were then incubated with a 1:250 dilution of either TRITC or FITC-conjugated phalloidin to stain actin filaments. Isolated DS were stained without fixation, so that they could be subject to electrophysiological analysis. Briefly, isolated DS were seeded on a coverslip, and incubated with 1:400 mouse anti-PSD-95 antibody, 1:200 donkey anti-mouse Cy3-conjugated secondary antibody (Santa Cruz Biotechnology, Santa Cruz, CA, United States) and 1:250 FITC-conjugated phalloidin. After 30 min, isolated DS were attached to coverslip and were washed with PBS to reduce background fluorescence. Images were captured with an inverted Olympus microscope (IX71, Olympus America, Center Valley, PA, United States) connected to a digital CCD camera (C4742-80-12AG, Hamamatsu Photonics, Bridgewater, NJ, United States). Images were collected and analyzed with the IPLab 4.0 (Scanalytics, Vienna, VA, United States) acquisition and analysis software.

### Calculations of dendritic spines enrichment

For quantitation studies, immunolabeling of the various sample preparations was conducted with two different monoclonal antibodies, namely K28/74 (PSD-95 MAGUK scaffold protein, dilution 1:10), and N327/95 (Glutamate receptor ionotropic, NMDA2A, dilution 1:10) from Developmental Studies Hybridoma Bank, University of Iowa (United States) to confirm the enrichment of DS in our preparation. The labeling was made on the fresh samples by incubation with both primary and secondary antibodies and FITC-phalloidin, from which co-labeling was explored in each case. The criteria for identifying a positive DS were the co-labeling of either PSD-95 and actin or Glutamate receptor and actin, always in combination with a DIC image consistent with a DS by shape and size (see Supplementary Appendix 1, 2). A quantitative assessment of enrichment was conducted as follows (see Supplementary Appendix 2):

Briefly, we marked in an aliquot of the pellet corresponding to step 1 of the centrifugation (Figure 1A) (“whole supernatant”, Supplementary Appendix 2) both actin and PSD-95, and we counted by field the number of times that rounded-shaped vesicles in DIC overlapped with the double marking (points with three positive criteria). We simultaneously counted the number of rounded structures in DIC, the number of phalloidin-labeled dots (green dots), and the number of PSD-95-labeled dots (red dots). Thus, we calculated for each aliquot of the pellet (prior to centrifugation in step 2) the following relationship:


R⁢a⁢t⁢i⁢o1=points⁢with⁢the⁢ 3⁢criteriaround⁢structures⁢in⁢DIC+green⁢points+red⁢points3

We performed the same procedure for band O2 of step 2 of the centrifugation (Figure 1), (“DS fraction”, Supplementary Appendix 2), which is the band that would contain the fraction enriched in DS, thus obtaining Ratio_2_. In each case, measurements were made from 4 aliquots, and the ratios were averaged. The enrichment was calculated as:


$$E\,n\,r\,i\,c\,h\,m\,e\,n\,t=\frac{\mathrm{Mean}\,{\mathrm{Ratio}}_{2}}{\mathrm{Mean}\,{\mathrm{Ratio}}_{1}}$$

In this way, we obtained the following values that were subjected to the t test:

Ratio 1 = 0.0995 ± 0.0398, Ratio 2 = 0.4520 ± 0.0781, Enrichment = 4.59, *p* = 0.007.

### Electron microscopy

High-resolution electron microscopy with a negative staining protocol of isolated DS was conducted at the Microscopy Core of the Membrane Biology Program at MGH. A sample (10 μl drop) of the original isolated DS preparation was applied to a formvar-coated gold-200 mesh grid (Electron Microscopy Sciences, Hatfield, PA, United States) for one minute. The sample was drawn off and replaced with a drop of 2.0% aqueous phosphotungstic acid (EMS, 10 s), which was drawn off and rinsed with drops of double distilled water and dried. Grids were examined in a JEOL JEM 1011 transmission electron microscope at 80 kV. Images were collected using an AMT (Advanced Microscopy Techniques, Danvers, MA, United States) digital imaging system.

### Electrophysiology. Dendritic spines and dendrite electrical recordings

Dendritic spines electrophysiological data were acquired with an Axon Patch 200B amplifier, low pass filtered at 10 kHz, and digitized with 1400A Digidata (Axon Instruments, Union City, CA, United States). The pCLAMP 10.0 software (Axon Instruments) was used to acquire and analyze the data. Patch pipettes were pulled from borosilicate glass (Garner Glass Co, Claremont, CA, United States) with a two-stage Narishige PB-7 vertical puller and then fire-polished to a resistance between 8–15 MΩ using a Narishige MF-9 microforge (Narishige International USA, East Meadow, NY, United States). For the cell-attached patch on DS of cultured hippocampal neurons, the bathing solution contained (in mM): 135 NaCl, 5 KCl, 1.2 CaCl_2_ and 10 HEPES (free acid), pH was adjusted to 7.4 with NaOH (cultured cells). For patches of isolated DS, the bathing solution contained (in mM): 135 KCl, 5 NaCl, 1 EGTA, and 10 HEPES, pH was adjusted to 7.4 with NaOH. Patch pipettes were filled with a solution containing (in mM): 135 NaCl, 5 KCl, 1.2 CaCl_2_, 10 HEPES (free acid), 100 μM glycine and 10 μM NMDA, pH was adjusted to 7.4 with NaOH. Tight seals were established on DS of cultured hippocampal neurons or isolated DS preparation under ×100 oil lens of the inverted Olympus (IX71) microscope. DS signals were recorded, as indicated, in the presence or absence of 10 μM NMDA and 100 μM glycine to stimulate NMDA receptors. Whenever indicated, the DS-attached pipette contained instead 20–60 μg/ml of the actin filament disrupter cytochalasin D. The DS membrane potentials were recorded under current-clamp mode without current injection. Whenever dual *in situ* DS-dendrite recordings were conducted, a second patch clamp amplifier (Dagan 3900A) was connected through a second pipette, which was kept in current mode throughout the experiment. The dendrite-attached pipette contained a saline solution with following composition (in mM): 135 KCl, 5 NaCl, 10 HEPES, 1 EGTA, pH 7.4, always in the absence of activating ligands.

### Electrophysiology. Double-electrode patch-clamping on cultured hippocampal neurons

Cultured hippocampal neurons were bathed in an extracellular saline solution containing (in mM): 135 NaCl, 5 KCl, 1.2 CaCl_2_, 10 HEPES, pH 7.4, and were viewed under objectives of ×60 and ×100. The dendrites were patched using a pipette filled with (in mM): 135 KCl, 5 NaCl, 10 HEPES, 1 EGTA, pH 7.4. The membrane voltages on dendrites were recorded under current clamp using Axopatch 200B amplifier (Axon instruments). At the same time, the DS from the same dendrites were patched using a pipette filled with the extracellular saline solution. In some cases, the saline solution also contained 10 μM NMDA and 100 μM Glycine to activate NMDA receptor, or 20–60 μM cytochalasin D (CD) to modulate the cytoskeletons. The electrical signals in DS were recorded in voltage clamp mode using a Dagan 3900A amplifier. The signal from both dendrites and DS were digitized with a 1440A Digidata (Axon instrument) and analyzed with pClamp10.0 software (Axon instrument).

### Electrophysiology. Lipid bilayer reconstitution of dendritic spines membranes

**FIGURE 3:**
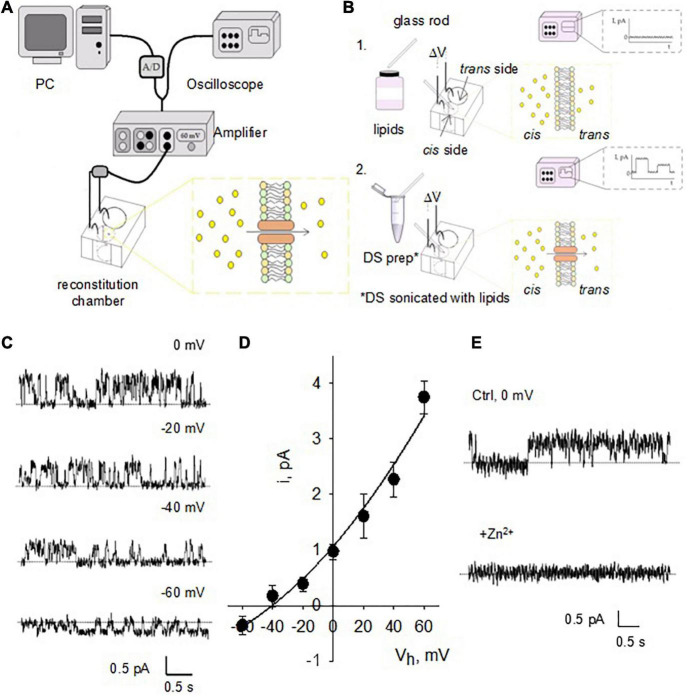
Single channel currents of reconstituted DS. **(A)** Design of the instruments used for BLM reconstitution. The hemi-chambers (*cis* and *trans*) that make up the reconstitution chamber are connected using agar bridges and 200 mM KCl solution to Ag/AgCl electrodes connected to the amplifier, high input impedance current-to-voltage converter. Through an analog-digital system, the signal is digitized and sent to a computer to be stored and later analyzed. The signal is observed in real time by means of an oscilloscope connected in parallel with the circuit. **(B)** The BLMs were formed with a POPC: POPE (7: 3) mixture (lipids) over an opening located on the wall of a polystyrene bucket (step 1). The cuvette was inserted into a chamber reconstitution, thus defining two compartments. Both compartments are filled with electrolyte solutions. The DS electrical activity was studied after insertion in the bilayer with a glass rod (step 2). Channel activity is observed as temporary fluctuations in current (oscilloscope) a once it is inserted. **(C)** Single channel currents from reconstituted DS were observed at various holding potentials after addition of NMDA to the *trans* side of the reconstitution chamber. **(D)** Current-to-voltage relationship of NMDA-activated channels similar to those shown in panel **c** reveals a conductance of 53.8 ± 5.2 pS (*n* = 4). Experimental values (filled circles) are the mean ± SEM from *n* = 4 experiments. Solid line is best fitting of data with a generalized GHK equation (see section “Materials and methods”). **(E)** NMDA-activated currents were readily inhibited by addition of either Zn^2+^ or Mg^2+^ (not shown) to the *cis* chamber (*n* = 3).

For the reconstitution of isolated DS in a lipid bilayer system, a lipid mixture was prepared with a content of 70% 1-palmitoyl-2-oleoyl-phosphatidyl-choline and 30% 1-palmitoyl-2-oleoyl phosphatidyl-ethanolamine in n-decane (∼20–25 mg/ml) (Figures 3A,B). Isolated DS were mixed with the above lipid solution at 1:1 ratio followed by brief sonication to form liposomes. The liposomes were painted with a glass rod to the aperture (150 μm diameter) of a polystyrene cuvette (CP13-150) that fits in a lipid bilayer chamber (model BCH-13, Warner Instruments Corp.). The *cis* side of the lipid bilayer was bathed with 150 mM KCl and 10 mM HEPES (pH 7.4), while the *trans* side of the bilayer was bathed with 15 mM KCl and 10 mM HEPES (pH 7.4). Channel activity and membrane oscillations were recorded in voltage-clamp mode.

### Chemicals

All chemicals and drugs were obtained from Sigma-Aldrich (St. Louis, MO, United States) unless otherwise stated.

### Statistics and data fitting

Values throughout the manuscript were expressed as the mean ± SEM, where *n* = number of individual experiments. Statistical significance was achieved with a *p* < 0.05. Single channel current-to-voltage relationships were fitted with a generalized Goldman-Hodgkin-Katz (GHK) equation of the form:


$$I=\sum_{C_{i}=1}^{n}\left(\frac{z_{i}^{2}\,F^{2}\,V\,P_{i}^{2}}{R\,T}\right)\,\left(\frac{C_{i}^{i\,n}-C_{i}^{o\,u\,t}\,e^{\frac{-z_{i}\,F\,V}{R\,T}}}{1-e^{\frac{-z_{i}\,F\,V}{R\,T}}}\right)$$

Where “*i*” is the type of ion, K^+^ and Cl^–^, *z* is the valence, *F* is the Faraday constant, *R* is the ideal gas constant, V (voltage) is the actual holding potential, T is temperature, *C_*i*_*^in^** and *C_*i*_*^out^** are the ionic concentrations on either side of the membrane. In BLM experiments, the inside represents the *cis* compartment, and the outside is the *trans* compartment of the hemi-chambers.

## Results

### Staining of isolated dendritic spines

Dendritic spines contained abundant actin structures and glutamate receptors, as indicated by phalloidin and anti-GluR2 antibody staining, respectively (Figure 2A) and NMDA2A (Supplementary Appendix 1). This finding also suggested that the isolation procedure primarily damaged the structured cytoskeleton, confirmed by electron microscopy (Figure 2B), where we observed multiple DS, both in dissociated and aggregated forms. It is important to note, however, that DS clustering may be enhanced by high electron density negative staining. DS were often observed as saccular vesicles, either open or closed, with postsynaptic density (PSD), and cleaner lumen (see for comparison Gray, 1959; Blackstad and Kjaerheim, 1961; Peters and Kaiserman-Abramof, 1970; Peters et al., 2008; García-López et al., 2010; Frotscher et al., 2014). Contrary to presynaptic components, which are most commonly axon terminals characterized by synaptic vesicular content that often mingle with mitochondria; the apposing postsynaptic elements in the CNS have features similar to any part of a neuron (Peters et al., 2008). The images in Figure 2 show freshly single DS, which were labeled with TRITC-phalloidin, displaying an abundant cytoskeleton. Isolated DS were of different sizes, and some of them appeared open. Many DS contained PSD (Figure 2C). The enriched DS preparation was assessed without any fixation or further procedures, such that any morphological features may be directly correlated with electrophysiological information. The material amounted to 58 μg protein/ml.

### Lipid bilayer reconstitution of isolated dendritic spines

To characterize the electrical properties of DS without interference from soma, dendritic shaft or other DS, we first reconstituted DS in a BLM system (Figure 3A) to characterize single channel currents. This standard technique has been extensively used in the reconstitution of ion channels of various origins, and the basics can be found in classic texts on the subject (see Miller, 1986; Hanke and Schlue, 1993). Briefly, the DS preparation was sonicated with lipids as described in Methods and incorporated in the bilayer membrane (Figure 3B) by painting the preparation with a glass rod. We observed NMDAR single-channel currents in the reconstituted DS, as expected (Figure 3C) in the presence of NMDA (10 mM) and Glycine (100 mM) in the *trans* chamber. The channels had a single channel conductance of 53.8 ± 5.2 pS (Figure 3D, mean ± SEM, *n* = 4) and were blocked by 1 mM Zn^2+^ applied through the *cis* chamber (Figure 3E, *n* = 3).

**FIGURE 4:**
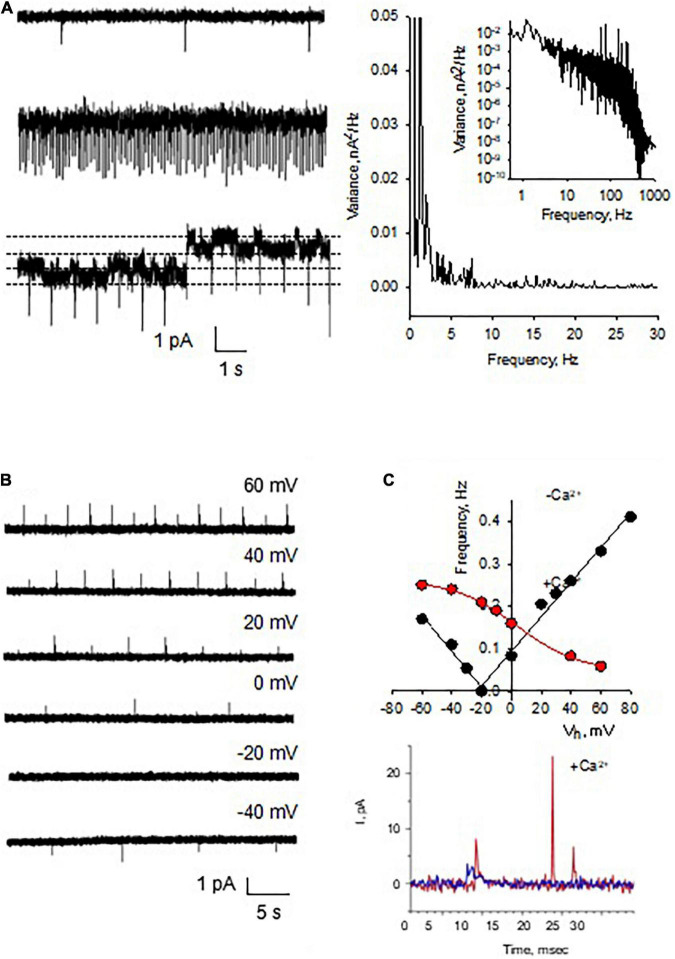
Spontaneous electric oscillations of reconstituted DS. (**A**, Left) Current oscillations (in voltage-clamp mode) in the form of spikes were observed in the reconstituted membranes (*n* = 36), which were independent of ion channel recordings, as observed in the bottom tracing where two channel levels were indicated by the horizontal lines. Right. Power spectra of tracings at Left, disclosing spontaneous oscillations with peak frequency at 1-2 Hz, and ion channel activity (Inset, showing a Lorentzian shoulder). **(B)** The spontaneous electrical oscillations were detected in the absence of NMDA added from the *trans* side of the chamber, whose frequency depended on the holding potential. **(C)** Spontaneous activity was strongly dependent on the presence of external (*trans*) Ca^2+^ (15 mM). The amplitude (as well as polarity) and frequency were strongly dependent on the presence of Ca^2+^. (Top panel) The black solid lines were the best linear fitting of the recorded frequencies vs. holding potential, with the function –0.0042*V* – 0.0749 (*R* = 0.9867) for negative values, and 0.0041*V* + 0.0949 (*R* = 0.9935) for positive values, respectively. The red line was the best fitting with a sigmoid function 0.0471 + 0.2150 (1 + exp(–3.6039*V*/21.1850))^– 1^ (*R* = 0.9994). Bottom panel shows representative Ca^2+^-dependent frequency spikes in the absence (Blue), and presence (Red) of the ion.

We also observed spontaneous activity in the form of electrical oscillations after reconstitution of DS membranes in the BLM reconstitution system (Figures 4A,B, *n* = 36), which were further explored with membrane-attached patch clamping both *in vitro* and *in situ*. The oscillations observed in the BLM system suggested that DS membranes contain the required active electrical components to support the generation of active electrical signals. The Fourier spectrum revealed ion-channel activity (Figure 4A, Right). Although the amplitude of these changes in membrane conductance was mainly unchanged, their frequency was strongly dependent on the holding potential, with almost complete inhibition at –20 mV (Figure 4C). To further explore the nature of these oscillations, we added Ca^2+^ (1 mM) to the bathing solution after the spontaneous generation of the oscillations, to mimic maximal Ca^2+^-channel activation. We observed a dramatic shift in the frequency and “reversal potential” of the spontaneous activity, which almost disappeared at positive, but not negative potentials (Figure 4C). The data suggest the presence of Ca^2+^-dependent oscillatory behavior, which is intrinsic to DS function. This, we believe is the first demonstration of such behavior, and raises the interesting possibility of being the electrical properties of DS not previously revealed.

### Patch clamping of isolated dendritic spines

**FIGURE 5:**
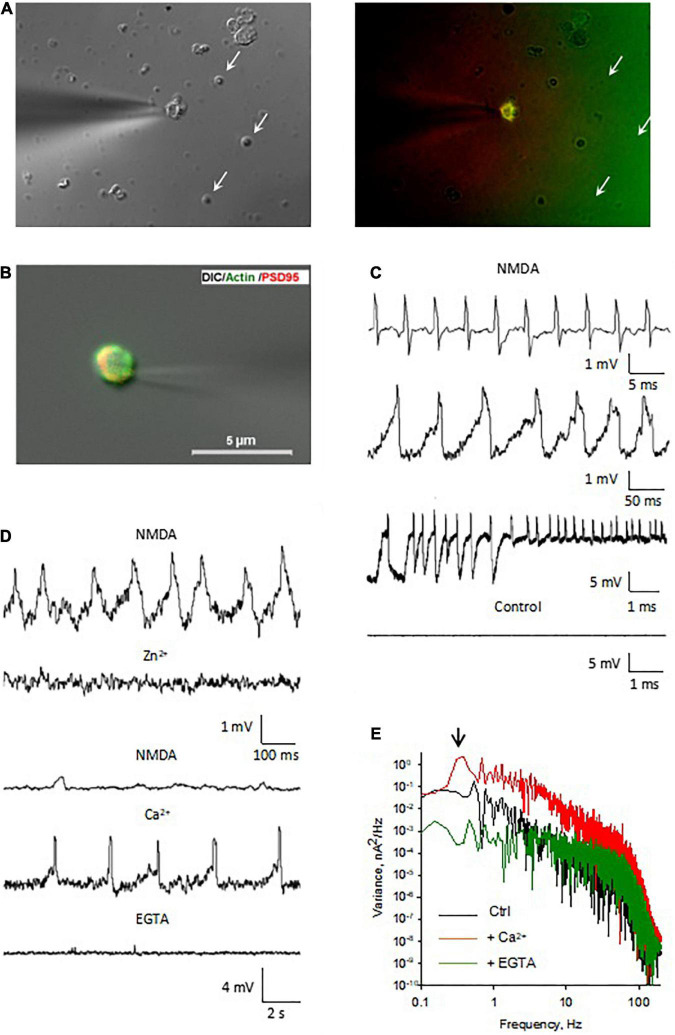
Electrical oscillations in isolated DS. (**A**, Left) We have been able to identify and select isolated DS for electrical manipulation. The patch pipette approached an isolated DS and the seal was obtained by gentle suction of the DS to the pipette (×100). Right. Several isolated DS are observed in the field, but only the DS approached with the patch pipette allowed immunocytochemical labeling with PSD-95 MAGUK, labeled in red and FITC-phalloidin labeled in green. This isolated and others readily labeled were used for the DS-attached approach to study single channel behavior and cytoskeletal manipulations. Spontaneously sealed DS (arrows) allowed “whole-cell” electrical manipulation after patching and breaking in, as shown below. **(B)** The patch pipette approached an isolated DS and the seal was obtained by gentle suction of the DS to the pipette (×60). **(C)** Several patterns of electrical oscillations were observed with NMDA in the pipette (*n* = 12). Electrical oscillations were usually absent in the absence of NMDA (*n* = 4). (**D**, Top). Application of Zn^2+^ (1 mM) to the bath blocked the NMDA-activated oscillations (*n* = 4). Bottom. The electrical oscillations of isolated DS were potentiated by increase in intracellular Ca^2+^ (1 mM Ca^2+^ to bath, *n* = 7) and decreased by EGTA (5 mM to the bath, *n* = 4). **(E)** Fourier spectra of the tracings in d. in the presence of NMDA (black line), and after subsequent addition of Ca^2+^ (Red line) and EGTA (Green line). The downward arrow indicates the fundamental frequency of the oscillations in the presence of Ca^2+^, but not in the control, or EGTA conditions.

We further assessed the electrical properties of isolated DS by membrane-attached patch clamping in the presence of NMDA (10 μM) and Glycine (100 μM) applied through the patch pipettes (Figures 5A,B). Voltage oscillations were often observed at diverse frequencies and amplitudes (Figure 5C, *n* = 12). These electrical oscillations were absent when NMDA was omitted from the pipettes (Figure 5C, “Control”, *n* = 4), suggesting that NMDAR activation might be required for this phenomenon to occur. This was further supported by the inhibitory effect of Zn^2+^ applied to the bath (1 mM, an NMDAR blocker), which eliminated the electrical oscillations (Figure 5D, *n* = 4). The DS electrical oscillations were also Ca^2+^-dependent, as an increase in intracellular Ca^2+^ (1–5 mM) activated oscillations in silent DS patches (Figure 5D, *n* = 7). Conversely, Ca^2+^ depletion by addition of EGTA (5–10 mM) to the bath eliminated the activated oscillations (Figure 5D, *n* = 4). Fourier analysis of the oscillations disclosed a peak frequency at 0.3 Hz in the presence, but not the absence of Ca^2+^ (Figure 5E).

### Voltage and current clamp of isolated dendritic spines

**FIGURE 6:**
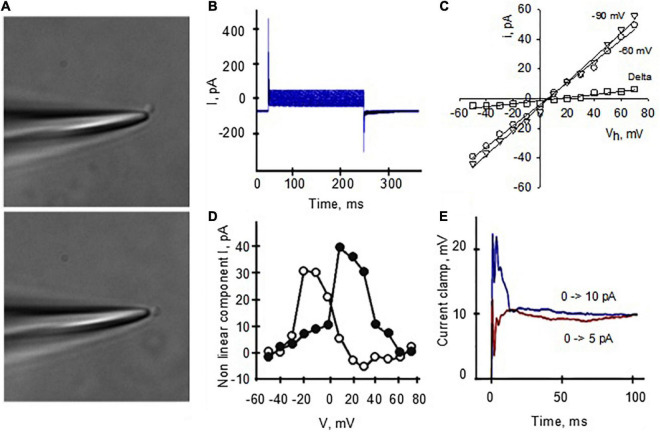
Voltage and current clamping of isolated DS. **(A)** The patch pipette, filled with a high KCl solution, approached an isolated DS and the seal was obtained by gentle suction of the DS to the pipette. Further suction allowed access to the lumen. **(B)** “Whole-cell” type DS currents under voltage clamp condition allowed the calculation of a linear DS conductance **(C)**, which was slightly dependent on the holding potential, 824 ± 174 pS (*r* = 0.9976, *n* = 13), and 721 ± 14.6 pS (*r* = 0.9978, *n* = 13), for resting potentials of –90 and –60 mV, respectively. The calculated delta between conductances was 0.10 nS (*r* = 0.9712). **(D)** Analysis of the tail currents after subtraction of the linear component shows voltage dependent conductance contribution consistent with both Na^+^ and K^+^ currents. **(E)** Voltages in the graph indicate holding potential prior to test. This experimental approach also allowed current clamp manipulations, which elicited action potentials.

Spontaneously sealed isolated DS (Figure 6A) were also patched to obtain “whole cell-like” voltage and current clamp information. The “whole-cell” voltage clamp configuration (Figure 6B) allowed the calculation of the isolated DS conductance (Figure 6C), which was only slightly dependent on the holding potential. Analysis of the tail currents (Figure 6D) after subtraction of the linear components showed voltage dependent conductance contributions consistent with both Na^+^ and K^+^ currents. This experimental approach also allowed current clamp manipulations, which elicited action potentials (Figure 6E).

### Electrophysiology of individual dendritic spines *in situ*

**FIGURE 7:**
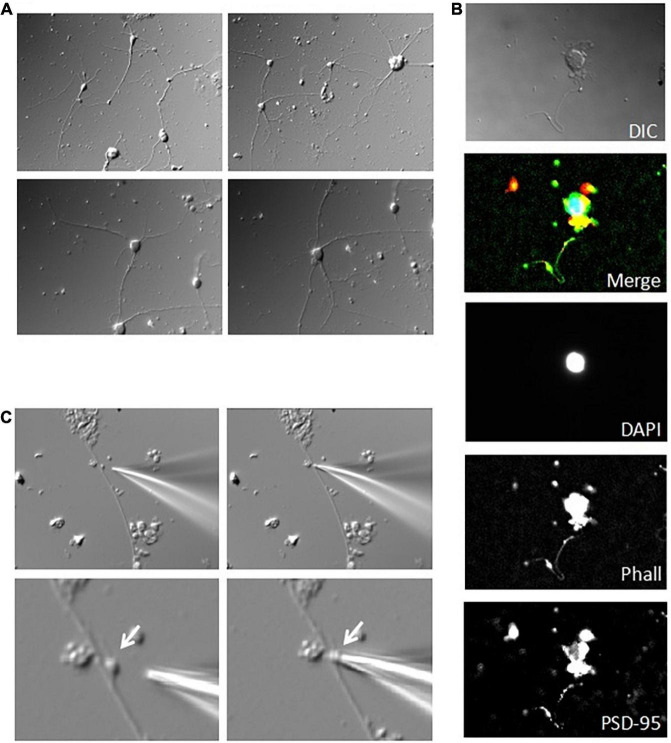
Patch clamping of DS in cultured hippocampal neurons. **(A)** Acutely dissociated adult mouse hippocampal neurons were sparsely placed and maintained in culture for up to two weeks. Images show a week old culture (×40). **(B)** Immunocytochemical labeling of cultured neurons showed little connectivity, and discrete level expression of PSD-95 (Red) and actin (Phalloidin staining, Green) (nuclear counter-staining with DAPI in Blue). From Top to Bottom images are merged and original DAPI, actin, and PSD-95 staining, respectively. Please note that single staining images were further contrasted to allow better identification of regions of interest. **(C)** Identifiable mushroom DS on the dendritic shaft of a cultured hippocampal neuron was amenable to patch clamping *in situ*, as shown by the approaching patch pipette (Left), and after seal (Right). Images are shown under lower (Top) (×40), and higher magnification (Bottom) (×100).

These *in vitro* studies clearly demonstrated the active properties of isolated DS. To further characterize the electrical properties of DS and to evaluate the possible role of connecting dendrites and soma, we patched DS *in situ* using cultured adult mouse hippocampal neurons maintained in culture medium for up to two weeks (Figure 7A). DS appear in different shapes and forms based on the relative sizes of the spine head and neck (Peters and Kaiserman-Abramof, 1970), including mushroom spines with a large head and a narrow neck, thin spines with a smaller head and a narrow neck, and stubby spines without constriction between the head and the attachment to the shaft. Neurons were labeled for actin, and PSD-95 to visualize DS, and DAPI counterstaining to label the nucleus (Figure 7B). Patch pipette was approach to DS *in situ* (Figure 7C) and obtained seals and electrical information.

**FIGURE 8:**
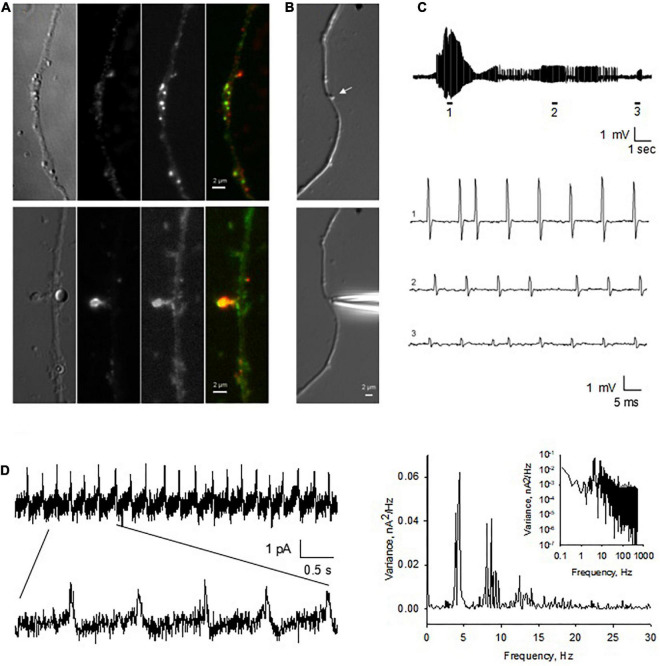
Endogenous electrical activity of DS in hippocampal neurons. **(A)** Cultured hippocampal neurons were maintained in culture for up to two weeks. Here shown a neurite of a one week-old culture. DS were observed to protrude from the dendritic shaft (×100). Immunocytochemical labeling showed elongated dendrites, with identifiable DS, as revealed by the high level expression of PSD-95 (Red) and actin staining (Phalloidin, Green) throughout the dendrites. On DS, usually strong actin staining was present, especially on large DS (Lower panels). **(B)** Identifiable (stubby) DS on the dendritic shaft of a cultured hippocampal neuron (Upper panel) (arrow). DS were amenable to patch clamping *in situ*, as shown by the approaching patch pipette. **(C)** Spontaneous as well as current-induced electrical oscillations were observed in most DS patched (*n* = 11). Electrical activity varied in amplitude and frequency even within the same DS (expanded traces 1–3). (**D**, Left) The spontaneous electrical signals showed a seemingly capacitive nature. (Right) Fourier power spectra of Left tracing showing at least three fundamental frequencies around 4, 9, and 13 Hz in Linear-Linear, and Log-Log (Inset), plots (representative of *n* = 3).

Elongating dendrites were often observed (Figures 8A,B), where stubby DS were observed to protrude from the dendritic shaft (Figure 8A). DS in cultured neurons were further identified by immunostaining, as revealed by high level expression of PSD-95 and the presence of actin networks (Figure 8A). Actin staining was especially prominent on large spines (Figure 8A, bottom panels). We performed the cell-attached patch clamping on DS from hippocampal neurons *in situ* (Figure 8B, bottom). Consistent with the findings in isolated DS, in the presence of NMDA (10 μM) and glycine (100 μM) in the pipette, and under current clamping conditions, we observed EPSP-resembling intrinsic oscillations on DS (Figure 8C, *n* = 11). Interestingly, these oscillations showed diverse amplitudes and frequencies, showing at least three fundamental frequencies around 4, 9, and 13 Hz (Figure 8D).

### Back-propagation of dendritic spines electrical signal along dendrites

**FIGURE 9:**
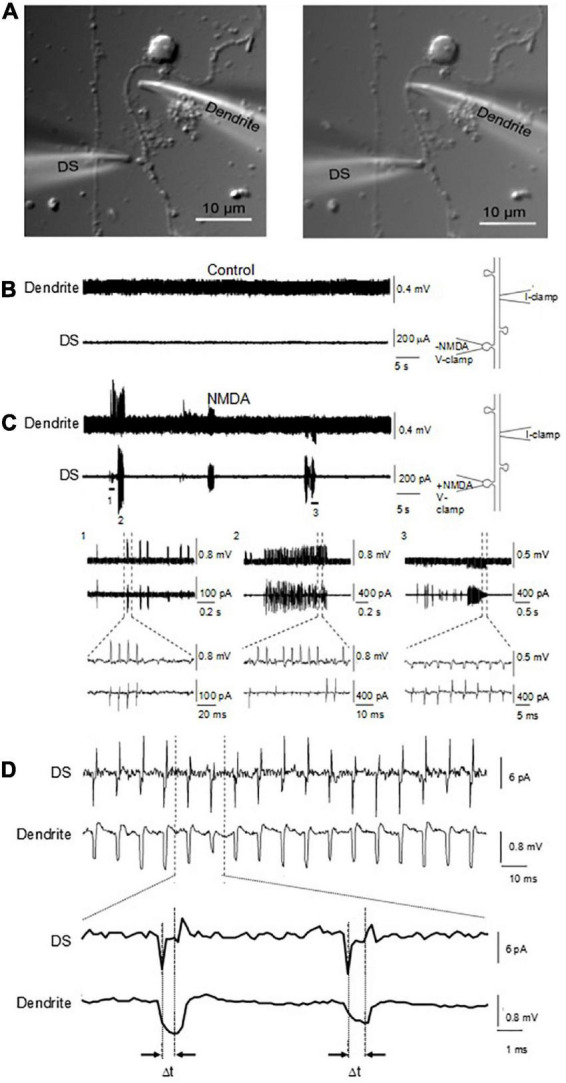
Coupling of DS-generated electrical activity to connecting dendrite and different waveforms. **(A)** Cultured adult mouse hippocampal neurons were maintained for up to one week in culture, where dendrite and DS were observed under DIC (×100). Under these conditions, electrical recordings were obtained by sealing and voltage clamping one electrode in a DS (DS), while the second electrode was current clamped in the sprouting dendrite (Dendrite) a few micrometers away. **(B)** In the absence of ligand, neither the DS nor the sprouting dendrite showed any electrical activity. **(C)** In the presence of ligand in the DS-attached pipette, however, in six out of twenty six experiments, NMDA-induced electrical oscillations were observed, that back propagated to the attached dendrite. Expanded tracings are shown at the bottom. Different waveforms and polarity were observed in a single experiment, including periodic and bursts of oscillations. **(D)** Delay of electrical signaling between DS and dendrite. The spontaneous electrical DS signals showed a seemingly capacitive nature.

To study how the electrical signals generated by the DS back propagated along dendrites, an important mechanism for signal integration, we performed double-electrode patch clamping recordings (Figure 9A). One electrode was sealed against the DS and voltage clamp was performed. The second electrode was sealed against the dendrite from which the DS sprouted, which was current clamped. NMDA (10 μM) and glycine (100 μM) were usually included in the DS pipettes, to activate NMDAR and electrical oscillations. As a control, in the absence of NMDA, no oscillations were observed on either dendrites or DS (Figure 9B, *n* = 18). In contrast, we found NMDA-activated electrical oscillations in both DS and dendrites in the presence of NMDA and glycine (Figure 9C, 6 of 26). The oscillations were coupled between DS and dendrites, suggesting the signal on dendrites are propagated from DS. Similar to findings with other techniques, oscillations in both DS and dendrites showed diverse frequencies and waveforms (Figure 9C), including short periodic (Figure 9C, 1), burst of oscillations (Figure 9C, 2), and oscillations of reverse polarity (Figure 9C, 3). The signaling ratio between DS and dendrites was also of wide range and showed quick transitions between large and small amplifications (Figure 9C), with the underlying mechanism yet to be studied. There were usually very short delays (∼20 μs) between the DS-generated waveforms, and those evoked at the dendritic site (Figure 9D).

### Cytoskeletal control of dendritic spines channel activity

**FIGURE 10:**
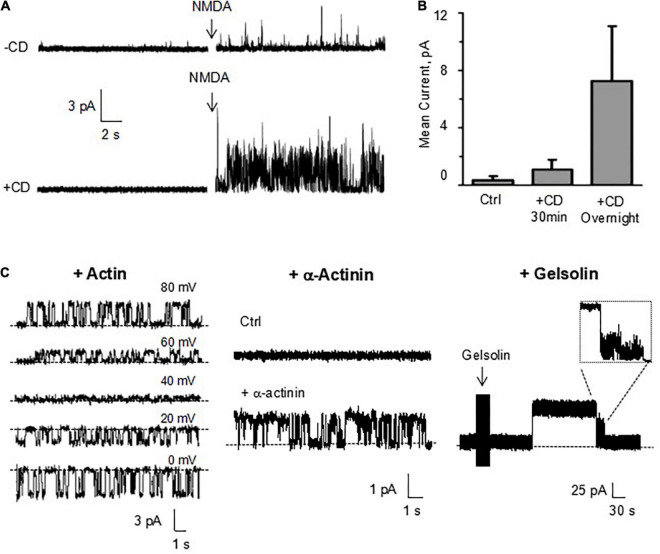
Effect of cytoskeletal manipulations on ion channels from reconstituted DS. **(A)** Channel activity was determined in DS membranes reconstituted in a lipid bilayer system. Cytochalasin D (CD, 10 μM) addition to the *cis* chamber increased instances of *trans* NMDA (10 μM) channel activation. **(B)** Average data of conditions in a, and longer (overnight) exposure of isolated DS to CD (10 μM) prior to reconstitution and activation by NMDA. Data are the mean ± SEM (*n* = 9, 11 and 6 for control, CD 30 min and CD overnight, respectively). (**C**, Left) In the absence of NMDA, addition of actin (20 μM) and ATP (1 mM) to the *cis* chamber was sufficient to activate channel currents in the presence of 150 mM KCl, and 15 mM NaCl in the *cis* chamber, and 150 mM NaCl, and 15 mM KCl in the *trans* chamber. In the absence of NMDA, the addition of either α-actinin (250 nM) (Middle) or gelsolin (Right) to the *cis* (cytosolic) chamber induced time-dependent ion channel activation. Recordings were obtained at 70 and 120 mV, respectively.

The actin cytoskeleton plays an essential role in the DS shape and function (Matus et al., 2000; Rao and Craig, 2000). In fact, it is thought that the actin cytoskeleton is instrumental in spinogenesis (Sekino et al., 2007; Zito et al., 2014). To date, however, information concerning a direct regulation by the actin cytoskeleton of DS ion channel activity and electrical function are unavailable. To determine whether the actin cytoskeleton regulates ion channel activity in DS, we explored the effect of the actin filament disrupter cytochalasin D (CD, 10 μg/ml) both in reconstituted DS with the BLM system, and membrane-attached conditions, in isolated DS. Addition of CD to the *cis* chamber increased instances of *trans* NMDA (10 μM) channel activation (Figures 10A,B). In the absence of NMDA, addition of actin (20 μM) and ATP (1 mM) to the *cis* chamber was sufficient to activate channel currents (Figure 10C, Left) with a current-to-voltage dependence. In the absence of NMDA, the addition of α-actinin (250 nM) to the *cis* (cytosolic) chamber induced time-dependent ion channel activation (Figure 10C, Middle). To further explore whether the actin cytoskeleton regulates DS ion channel activity, the effect of the actin severing protein gelsolin (10 μg/ml) was also assessed. Addition of gelsolin induced time-dependent channel activation (Figure 10C, Right), suggesting that dynamic changes in cytoskeletal organization help modulating DS electrical activity. The effect of gelsolin also confirms our original observations that the actin filament disrupter cytochalasin D modulates DS electrical activity and channel behavior.

**FIGURE 11:**
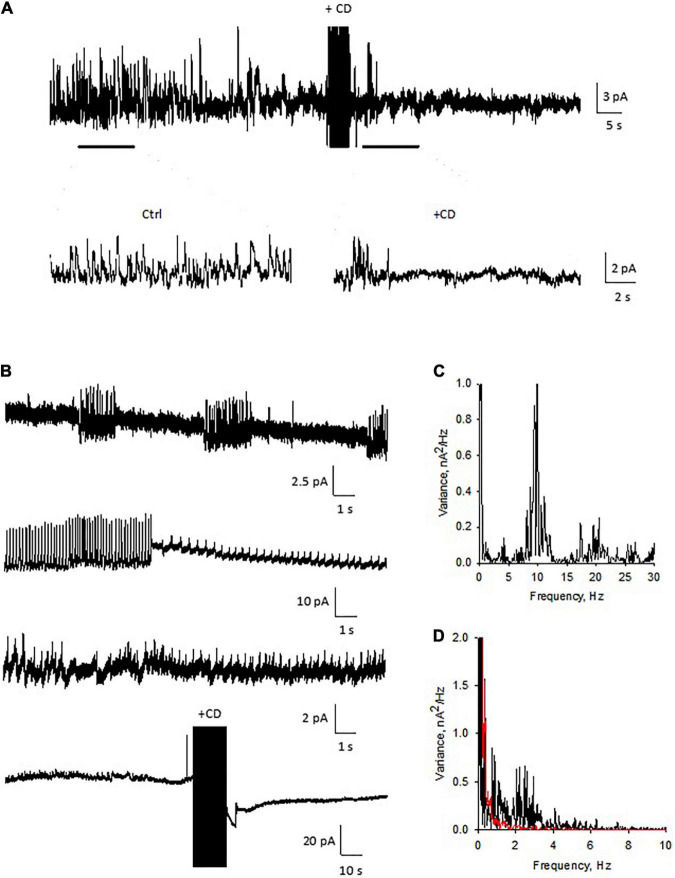
Patch clamping of isolated dendritic spines. **(A)** Electrical oscillations were often observed, with different regimes (*n* = 4). Holding potential for these representative recordings was 100 mV. Addition of cytochalasin D (CD, 10 μM) to the bathing solution modified the spontaneous oscillations elicited by isolated DS. Data are representative of three experiments. **(B)** Several patterns of electrical oscillations could be observed under spontaneous conditions, which varied at different holding potentials. The addition of cytochalasin D (10 μM) abolished DS electrical activity (*n* = 3). **(C)** Power spectrum of second tracing in panel **(B)**, showing at least three fundamental frequencies at between 10 and 20 Hz. **(D)** Fourier power spectra before (Black) and after (Red) CD. The actin filament disrupter completely eliminated the spontaneous electrical activity of the isolated DS.

To further evaluate the role of the actin cytoskeleton in the electrical activity of DS, we also performed membrane-attached patch clamping of isolated DS. Similar to experiments in the current clamp mode, the voltage-clamped DS showed intrinsic electrical activity (Figure 11) that was largely diminished (Figures 11A,B, *n* = 3) when we disrupted the actin cytoskeletal network with CD. This dramatic effect of CD confirmed the importance of the cytoskeleton on the intrinsic oscillatory function of DS, as shown by the spectral density in the Fourier analysis (Figures 11C,D).

## Discussion

Since the discovery of the dendritic spines by Ramón y Cajal in the late 19th century (see García-López et al., 2007 for a review), knowledge about these neuronal structures has increased enormously. However, because of their small size and dynamic nature direct electrical information from individual DS has been lacking. Thus, many key questions about their function have remained unanswered. The presence of NMDA and AMPA receptors (Yuste et al., 1999; Kovalchuk et al., 2000), and several voltage-, and ligand-gated ion channels, including various Ca^2+^ channels and glutamate and GABA receptors (Nusser et al., 1998) have been confirmed in DS, although their function and their contribution to the DS conductance have yet to be determined.

To bridge this gap, and particularly because of our interest in obtaining a suitable preparation to study ion channel-cytoskeletal connections, in this study we developed a preparation of isolated DS from the adult mouse hippocampus that was amenable to electrophysiological studies, including patch clamping and BLM reconstitution. The enriched DS preparation contained structures of different sizes and complexity, including small “round” sealed DS, as well as open and larger multi-headed DS. By both approaches we identified a ∼50 pS cation-selective ion channel that was activated by NMDA and Ca^2+^, and was inhibited by Zn^2+^. Another seldom observed cation channel had a large single channel conductance of 211 pS (data not shown). We also observed NMDA-induced electrical oscillations that acted concurrently with single ion channel activities and that were both profoundly modulated by changes in the actin cytoskeleton with the F-actin disrupter cytochalasin D and addition of actin-binding proteins. Single channel currents were activated by addition of either external actin, the actin bundling protein α-actinin, or the F-actin cleaving gelsolin in the presence of Ca^2+^. The data support the idea that the individual DS is a functional interface that engages in dynamic interplay between ligand-activated ion channels and intrinsic actin networks.

Perhaps the most interesting finding with this preparation is the presence of self-sustained electrical oscillations. This is in agreement with the fact that several hippocampal preparations both *in vivo* and *in vitro* generate various regimes of electrical oscillations that may invoke the electrical activity of specific ion channel species, including Ca^2+^ channels. The mammalian hippocampus presents endogenous electrical oscillations, particularly slow waves known as theta-alpha frequencies (Arnolds et al., 1980) that have been linked to mnemonic processes (Miller, 1989), and generates some of the largest EEG signals as theta waves (Buzsáki, 2002) that synchronize in traveling wave patterns (Lubenov and Siapas, 2009). The intrinsic oscillatory properties of hippocampal cells (Llinás, 1988) have been associated with identifiable ionic conductances including GABAA and NMDA receptors (Kramis et al., 1975; Soltesz and Deschénes, 1993), dendritic Ca^2+^ currents that amplify NMDA receptor-activated somatic oscillations, and include several Ca^2+^ channel isotypes. It has been suggested that a high density of voltage-gated (or Ca^2+^-dependent) Na^+^ and K^+^ permeable channels would endow DS with the ability to induce action potentials (Perkel and Perkel, 1985). Tsay and Yuste (2004) have postulated that localized changes in DS channel density would make them “hot spots,” to promote electrical conduction. DS-generated action potentials could spread to neighboring DS, triggering a chain of regenerative events enabling sustained signal and implementing logical operations (Shepherd and Brayton, 1987). Our present data on the current clamp-induced action potentials in closed DS, and the intrinsic electrical oscillations with identifiable frequencies agree with this hypothesis. This was also observed in the propagated signals in the DS-dendrite coupling. Our data indicated that NMDA stimulation of a single DS was able to backpropagate several micrometers into the dendrite, and elicit bursts and periodic oscillations, which were only delayed by fewer than 20 μs, regardless of distance from the DS. The Ca^2+^ signaling events in the DS may also trigger the Ca^2+^-dependent remodeling of the DS cytoskeleton, in agreement with the effects of gelsolin and α-actinin observed here.

It has long been speculated that memory storage in the brain implicates altering the strength of large assemblies of interconnected neurons (Nadel et al., 1975), where synaptic plasticity may constitute the physical correlates of memory storage (Kandel and Squire, 2000). However, the “intrinsic electrical activity” in neurons (Llinás, 1988) that includes both passive and active membrane characteristics, does not include the neuronal cytoskeleton, which is essential to experience-related plasticity, and changes associated with neural stimulation (Honkura et al., 2008).

Actin cytoskeletal dynamics is essential in DS morphogenesis and regulated plasticity (Matus et al., 2000; Rao and Craig, 2000; Sekino et al., 2007; Zito et al., 2014). Actin-associated morphological changes in DS correlate with LTP in the hippocampal tissue (Yuste and Majewska, 2001). Interestingly, various studies have disclosed relevant non-linear electrical properties of cytoskeletal polymers. Actin filaments behave like “cables” that act as transmission lines with the ability to conduct ion condensation waves (Lin and Cantiello, 1993), and microtubules (MTs) in turn, generate, propagate and amplify electrical oscillations (Priel et al., 2006; Cantero et al., 2016, 2018). Thus, the neuronal cytoskeleton may play a relevant role in the generation and encoding of electrical information at the subcellular level; thus contributing to the modulation of DS ion channel activity, and the formation of such events as synaptic strengthening, LTP, and memory enhancement and consolidation (Nelson et al., 2004). Rat brain MTs displayed self-sustained electrical oscillations (Cantero et al., 2018), and MT bundles also elicited highly synchronized trains of current oscillations that mimicked bursts of action potentials. We further determined that actin polymerization amplified the electrical oscillations of brain MTs as well (Cantero et al., 2020). Actin filaments and MTs interact with each other for intrinsic structural support in the formation of axons to send, and dendritic networks to receive synaptic signals, respectively (Stiess and Bradke, 2011). It is expected therefore, that cytoskeletal polymers may also contribute to the intrinsic electrical properties of DS.

Our present data suggest that DS electrical activity is a complex interplay between membrane-associated channels and the underlying cytoskeleton. Thus, ligand-gated Ca^2+^ influx might modulate two levels of activity: the first one by interaction with Ca^2+^ dependent cytoskeletal structures that remodel the actin cytoskeleton, and the second one by targeting feedback responses of DS channel activity. Conversely, cytoskeletal-remodeling feeds back on several DS ion channels, including NMDA receptors and other ligand-gated, as well as voltage-gated channels. The present study demonstrated that DS are active sites of ion channel-cytoskeleton interactions.

In conclusion, we obtained direct electrical single channel and whole-DS conductance data, showing intrinsically non-linear activity in the form of electrical oscillations and action potentials that provides experimental evidence for DS to be highly elaborate electrical compartments that contribute actively to neuronal behavior.

## Data availability statement

The raw data supporting the conclusions of this article will be made available by the authors, without undue reservation.

## Ethics statement

This animal study was reviewed and approved by ECOR at the Massachusetts General Hospital.

## Author contributions

AP, X-QD, NS, and HC carried out experimental procedures. MdRC and HC conducted the analysis of the experimental data and prepared the figures. AP, X-ZC, and HC designed all the experiments. HC and MdRC wrote the main manuscript text. All authors contributed to the article and approved the submitted version.

## References

[B1] Ackermann M. Matus A. (2003). Activity-induced targeting of profilin and stabilization of dendritic spine morphology. *Nat. Neurosci.* 6 1194–1200. 10.1038/nn1135 14555951

[B2] Agassandian C. Plantier M. Fattoum A. Represa A. Der Terrosian E. (2000). Subcellular distribution of calponin and caldesmon in rat hippocampus. *Brain Res.* 887 444–449. 10.1016/S0006-8993(00)03030-4 11134639

[B3] Alonso G. Widmer H. (1997). Clustering of KV4.2 potassium channels in postsynaptic membrane of rat supraoptic neurons: An ultrastructural study. *Neuroscience* 77 617–621. 9070739 10.1016/s0306-4522(96)00561-1

[B4] Araya R. Vogels T. P. Yuste R. (2014). Activity-dependent dendritic spine neck changes are correlated with synaptic strength. *Proc. Ntal. Acad. Sci.U.S.A.* 30 E2895–E2904. 10.1073/pnas.1321869111 24982196 PMC4104910

[B5] Arnolds D. E. Lopes da Silva F. H. Aitink J. W. Kamp A. Boeijinga P. (1980). The spectral properties of hippocampal EEG related to behaviour in man. *Electroencephalogr. Clin. Neurophysiol.* 50 324–328. 10.1016/0013-4694(80)90160-1 6160974

[B6] Blackstad T. W. Kjaerheim A. (1961). Special axo-dendritic synapses in the hippocampal cortex: Elctron and light microscopic studies on the layer of mossy fibers. *J. Comp. Neurol.* 117, 133–159. 13869693 10.1002/cne.901170202

[B7] Bonilla-Quintana M. Wörgötter F. Tetzlaff C. Fauth M. (2020). Modeling the shape of synaptic spines by their actin dynamics. *Front. Synaptic Neurosci.* 12:9. 10.3389/fnsyn.2020.00009 32218728 PMC7078677

[B8] Bourne J. N. Harris K. M. (2008). Balancing structure and function at hippocampal dendritic spines. *Annu. Rev. Neurosci.* 31 47–67. 10.1146/annurev.neuro.31.060407.125646 18284372 PMC2561948

[B9] Buzsáki G. (2002). Theta oscillations in the hippocampus. *Neuron* 33 325–240. 10.1016/S0896-6273(02)00586-X 11832222

[B10] Caldwell J. H. Schaller K. L. Lasher R. S. Peles E. Levinson S. R. (2000). Sodium channel Na(v)1.6 is localized at nodes of Ranvier, dendrites, and synapses. *Proc. Natl. Acad. Sci. U.S.A.* 97 5616–5620. 10.1073/pnas.090034797 10779552 PMC25877

[B11] Cantero M. R. Gutierrez B. C. Cantiello H. F. (2020). Actin filaments modulate electrical activity of brain microtubule protein two-dimensional sheets. *Cytoskeleton* 77 167–177. 10.1002/cm.21596 31953911

[B12] Cantero M. R. Perez P. L. Smoler M. Villa Etchegoyen C. Cantiello H. F. (2016). Electrical oscillations in two-dimensional microtubular structures. *Sci. Rep.* 6:27143. 10.1038/srep27143 27256791 PMC4891677

[B13] Cantero M. R. Villa Etchegoyen C. Perez P. L. Scarinci N. Cantiello H. F. (2018). Bundles of brain microtubules generate electrical oscillations. *Sci. Rep.* 8:11899. 10.1038/s41598-018-30453-2 30093720 PMC6085364

[B14] Cartailler J. Kwon T. Yuste R. Holcman D. (2018). Deconvolution of voltage sensor time series and electro-diffusion modeling reveal the role of spine geometry in controlling synaptic strength. *Neuron* 97 1126–1136. 10.1016/j.neuron.2018.01.034 29429935 PMC5933057

[B15] Drake C. T. Bausch S. B. Milner T. A. Chavkin C. (1997). GIRK1 immunoreactivity is present predominantly in dendrites, dendritic spines, and somata in the CA1 region of the hippocampus. *Proc. Natl. Acad. Sci. U.S.A.* 94 1007–1012. 10.1073/pnas.94.3.1007 9023373 PMC19630

[B16] Engert F. Bonhoeffer T. (1999). Dendritic spine changes associated with hippocampal long-term synaptic plasticity. *Nature* 399 66–70. 10.1038/19978 10331391

[B17] Fifkova E. Delay R. J. (1982). Cytoplasmic actin in neuronal processes as a possible mediator of synaptic plasticity. *J. Cell Biol.* 95 345–350. 10.1083/jcb.95.1.345 6890558 PMC2112353

[B18] Fischer M. Kaech S. Wagner U. Brinkhaus H. Matus A. (2000). Glutamate receptors regulate actin-based plasticity in dendritic spines. *Nat. Neurosci.* 3 887–894. 10.1038/78791 10966619

[B19] Frotscher M. Studer D. Graber W. Chai X. Nestel S. Zhao S. (2014). Fine structure of synapses on dendritic spines. *Front. Neuroanat.* 8:94. 10.3389/fnana.2014.00094 25249945 PMC4158982

[B20] Furukawa K. Fu W. Li Y. Witke W. Kwiatkowski D. J. Mattson M. P. (1997). The actin-severing protein gelsolin modulates calcium channel and NMDA receptor activities and vulnerability to excitotoxicity in hippocampal neurons. *J. Neurosci.* 17 8178–8186. 10.1523/JNEUROSCI.17-21-08178.1997 9334393 PMC6573728

[B21] Gamble E. Koch C. (1987). The dynamics of free calcium in dendritic spines in response to repetitive synaptic input. *Science* 236 1311–1315. 10.1126/science.3495885 3495885

[B22] García-López P. García-Marín V. Freire M. (2007). The discovery of dendritic spines by Cajal in 1888 and its relevance in the present neuroscience. *Prog. Neurobiol.* 83 110–130. 10.1016/j.pneurobio.2007.06.002 17681416

[B23] García-López P. García-Marín V. Freire M. (2010). Dendritic spines and development: Towards a unifying model of spinogenesis-a present day review of cajal’s histological slides and drawings. *Neural Plast*. 2010:769207. 10.1155/2010/769207 21584262 PMC3091278

[B24] Gray E. G. (1959). Electron microscopy of synaptic contacts on dendritic spines of the cerebral cortex. *Nature* 183 1592–1594. 10.1038/1831592a0 13666826

[B25] Halpain S. Hipolito A. Saffer L. (1998). Regulation of F-actin stability in dendritic spines by glutamate receptors and calcineurin. *J. Neurosci.* 18 9835–9844. 10.1523/JNEUROSCI.18-23-09835.1998 9822742 PMC6793298

[B26] Hanke W. Schlue W.-R. (1993). *Biological Techniques: Planar Lipid Bilayers, Methods and Applications.* London: Academic Press.

[B27] Harris K. M. (1999). Structure, development, and plasticity of dendritic spines. *Curr. Opin. Neurobiol.* 9 343–348. 10.1016/S0959-4388(99)80050-6 10395574

[B28] Harris K. M. Kater S. B. (1994). Dendritic spines: Cellular specializations imparting both stability and flexibility to synaptic function. *Annu. Rev. Neurosci.* 17 341–371. 10.1146/annurev.ne.17.030194.002013 8210179

[B29] Holthoff K. Tsai D. Yuste R. (2002). Calcium dynamics of spines depend on their dendritic location. *Neuron* 33 425–437. 10.1016/S0896-6273(02)00576-7 11832229

[B30] Honkura N. Matsuzaki M. Noguchi J. Ellis-Davies G. C. R. Kasai H. (2008). The subspine organization of actin fibers regulates the structure and plasticity of dendritic spines. *Neuron* 57 719–729. 10.1016/j.neuron.2008.01.013 18341992

[B31] Hotulainen P. Hoogenraad C. C. (2010). Actin in dendritic spines: Connecting dynamics to function. *J. Cell Biol.* 189 619–629. 10.1083/jcb.201003008 20457765 PMC2872912

[B32] Jayant K. Hirtz J. J. Jen-La Plante I. Tsai D. M. De Boer W. D. A. M. Semonche A. (2017). Targeted intracellular voltage recordings from dendritic spines using quantum-dot-coated nanopipettes. *Nat. Nanotechnol.* 12 335–342. 10.1038/nnano.2016.268 27941898 PMC5901699

[B33] Kandel E. F. Squire L. F. (2000). Neuroscience: Breaking down scientific barriers to the study of brain and mind. *Science* 290 1113–1120. 10.1126/science.290.5494.1113 11185010

[B34] Kiebler M. A. López-García J. C. Leopold P. H. (1999). Purification and characterization of rat hippocampal CA3-dendritic spines associated with mossy fiber terminals. *FEBS Lett.* 445 80–86. 10.1016/S0014-5793(99)00077-0 10069378

[B35] Kovalchuk Y. Eilers J. Lisman J. Konnerth A. (2000). NMDA receptor-mediated subthreshold Ca^2+^ signals in spines of hippocampal neurons. *J. Neurosci.* 20 1791–1799. 10.1523/JNEUROSCI.20-05-01791.2000 10684880 PMC6772937

[B36] Kramis R. Vanderwolf C. H. Bland B. H. (1975). Two types of hippocampal rhythmical slow activity in both the rabbit and the rat: Relations to behavior and effects of atropine, diethyl ether, urethane, and pentobarbital. *Exp. Neurol.* 49 58–85. 10.1016/0014-4886(75)90195-8 1183532

[B37] Leuner B. Shors T. J. (2004). New spines, new memories. *Mol. Neurobiol.* 29 117–130. 10.1385/MN:29:2:117 15126680 PMC3279151

[B38] Lin E. Cantiello H. F. (1993). Novel method to study the electrodynamic behavior of actin filaments. The cable properties of actin filaments. *Biophys. J.* 65 1371–1378. 10.1016/S0006-3495(93)81188-3 8274631 PMC1225863

[B39] Llinás R. R. (1988). The intrinsic electrophysiological properties of mammalian neurons: Insights into central nervous system function. *Science* 242 1654–1664. 10.1126/science.3059497 3059497

[B40] Lohmann C. Bonhoeffer T. (2008). A role for local calcium signaling in rapid synaptic partner selection by dendritic filopodia. *Neuron* 59 253–260. 10.1016/j.neuron.2008.05.025 18667153

[B41] Lubenov E. V. Siapas A. G. (2009). Hippocampal theta oscillations are travelling waves. *Nature* 459 534–539. 10.1038/nature08010 19489117

[B42] Lynch G. Larson J. Kelso S. Barrionuevo G. Schottler F. (1983). Intracellular injections of EGTA block induction of hippocampal long-term potentiation. *Nature* 305 719–721. 10.1038/305719a0 6415483

[B43] Malenka R. C. Kauer J. A. Zucker R. S. Nicoll R. A. (1988). Postsynaptic calcium is sufficient for potentiation of hippocampal slice transmission. *Science* 242 81–84. 10.1126/science.2845577 2845577

[B44] Matsuoka Y. Li X. Bennett V. (1998). Adducin is an in vivo substrate for protein kinase C: Phosphorylation in the MARKS-related domain inhibits activity in promoting spectrin-actin complexes and occurs in many cells, including dendric spines of neurons. *J. Cell Biol.* 142 485–497. 10.1083/jcb.142.2.485 9679146 PMC2133059

[B45] Matus A. (2000). Actin-based plasticity in dendritic spines. *Science* 290 754–758. 10.1126/science.290.5492.754 11052932

[B46] Matus A. Brinkhaus H. Wagner U. (2000). Actin dynamics in dendritic spines: A form of regulated plasticity at excitatory synapses. *Hippocampus* 10 555–560. 10.1002/1098-1063(2000)10:5<555::AID-HIPO5>3.0.CO;2-Z 11075825

[B47] Miller C. (1986). *Ion Channel Reconstitution.* New York: Plenum Press. 10.1007/978-1-4757-1361-9

[B48] Miller R. (1989). Cortico-hippocampal interplay: Self-organizing phase-locked loops for indexing memories. *Psychobiology* 17 115–128. 10.3758/BF03337827

[B49] Mills L. R. Niesen C. E. So A. P. Carlen P. L. Pigelman I. Jones O. T. (1994). N-type Ca^2+^ channels are located on somata, dendrites, and a subpopulation of dendritic spines on live hippocampal pyramidal neurons. *J. Neurosci.* 14 6815–6824. 10.1523/JNEUROSCI.14-11-06815.1994 7525892 PMC6577227

[B50] Muller D. Toni N. Buchs P. A. (2000). Spine changes associated with long-term potentiation. *Hippocampus* 10 596–604. 10.1002/1098-1063(2000)10:5<596::AID-HIPO10>3.0.CO;2-Y 11075830

[B51] Nadel L. O’Keefe J. Black A. (1975). Slam on the brakes: A critique of Altman, Brunner, and Bayer’s response-inhibition model of hippocampal function. *Behav. Biol.* 14 151–162. 10.1016/S0091-6773(75)90148-0 1137539

[B52] Nelson T. J. Backlund P. S. Jr. Alkon D. L. (2004). Hippocampal protein-protein interactions in spatial memory. *Hippocampus* 14 46–57. 10.1002/hipo.10152 15058482

[B53] Nusser Z. Lujan R. Laube G. Roberts J. D. Molnar E. Somogyi P. (1998). Cell type and pathway dependence of synaptic AMPA receptor number and variability in the hippocampus. *Neuron* 21 545–559. 10.1016/S0896-6273(00)80565-6 9768841

[B54] Oertner T. G. Matus A. (2005). Calcium regulation of actin dynamics in dendritic spines. *Cell Calcium* 37 477–482. 10.1016/j.ceca.2005.01.016 15820396

[B55] Palmer L. M. Stuart G. J. (2009). Membrane potential changes in dendritic spines during action potentials and synaptic input. *J. Neurosci.* 29 6897–6903. 10.1523/JNEUROSCI.5847-08.2009 19474316 PMC6665597

[B56] Pchitskaya E. Bezprozvanny I. (2020). Dendritic spines shape analysis: Classification or Clusterization? Perspective. *Front. Synaptic Neurosci* 12:31. 10.3389/fnsyn.2020.00031 33117142 PMC7561369

[B57] Perkel D. H. Perkel D. J. (1985). Dendritic spines: Role of active membrane in modulating synaptic efficacy. *Brain Res.* 325 331–335. 10.1016/0006-8993(85)90334-8 2579708

[B58] Peters A. Kaiserman-Abramof I. R. (1970). The small pyramidal neuron of the rat cerebral cortex. The perikaryon, dendrites and spines. *Am. J. Anat.* 127 321–355. 10.1002/aja.1001270402 4985058

[B59] Peters A. Sethares C. Luebke J. I. (2008). Synapses are lost during aging in the primate prefrontal cortex. *Neuroscience* 152, 970–981. 10.1016/j.neuroscience.2007.07.014 18329176 PMC2441531

[B60] Popovic M. A. Carnevale N. Rozsa B. Zecevic D. (2015). Electrical behaviour of dendritic spines as revealed by voltage imaging. *Nat. Commun.* 6:8436. 10.1038/ncomms9436 26436431 PMC4594633

[B61] Popovic M. A. Gao X. Carnevale N. T. Zecevic D. (2014). Cortical dendritic spine heads are not electrically isolated by the spine neck from membrane potential signals in parent dendrites. *Cereb. Cortex* 24 385–395. 10.1093/cercor/bhs320 23054810 PMC3888368

[B62] Priel A. Ramos A. J. Tuszynski J. A. Cantiello H. F. (2006). A biopolymer transistor: Electrical amplification by microtubules. *Biophys. J.* 90 4639–4643. 10.1529/biophysj.105.078915 16565058 PMC1471843

[B63] Ramón y Cajal S. (1891). Significación fisiológica de las expansiones protoplásmicas y nerviosas de la sustancia gris. *Rev. Ciencias Méd. Barc.* 22:23.

[B64] Rao A. Craig A. M. (2000). Signaling between the actin cytoskeleton and the postsynaptic density of dendritic spines. *Hippocampus* 10 527–541. 10.1002/1098-1063(2000)10:5<527::AID-HIPO3>3.0.CO;2-B 11075823

[B65] Rose C. Konnerth A. (2001). NMDA receptor-mediated Na^+^ signals in spines and dendrites. *J. Neurosci.* 21 4207–4214. 10.1523/JNEUROSCI.21-12-04207.2001 11404406 PMC6762772

[B66] Sekino Y. Kojima N. Shirao T. (2007). Role of actin cytoskeleton in dendritic spine morphogenesis. *Neurochem. Internat.* 51 92–104. 10.1016/j.neuint.2007.04.029 17590478

[B67] Shepherd G. M. Brayton R. K. (1987). Logic operations and properties of computer-simulated interactions between excitable-dendritic spines. *Neuroscience* 21 151–165. 10.1016/0306-4522(87)90329-0 3601072

[B68] Shepherd G. M. Brayton R. K. Miller J. P. Segev I. Rinzel J. Rall W. (1985). Signal enhancement in distal cortical dendrites by means of interactions between active dendritic spines. *Proc. Natl. Acad. Sci. U.S.A.* 82 2192–2195. 10.1073/pnas.82.7.2192 3856892 PMC397519

[B69] Shim K. S. Lubec G. (2002). Drebrin, a dendritic spine protein, is manifold decreased in brains of patients with Alzheimer’s disease and Down syndrome. *Neurosci. Lett.* 324 209–212. 10.1016/S0304-3940(02)00210-0 12009525

[B70] Skoff R. P. Hamburger V. (1974). Fine structure of dendritic and axonal growth cones in embryonic chick spinal cord. *J. Comp. Neurol.* 153 107–147. 10.1002/cne.901530202 4810722

[B71] Soltesz I. Deschénes M. (1993). Low- and high-frequency membrane potential oscillations during theta activity in CA1 and CA3 pyramidal neurons of the rat hippocampus under ketamine-xylazine anesthesia. *J. Neurophysiol.* 70 97–116. 10.1152/jn.1993.70.1.97 8395591

[B72] Sorra K. E. Fiala J. C. Harris K. M. (1998). Critical assessment of the involvement of perforations, spinules, and spine branching in hippocampal synapse formation. *J. Comp. Neurol.* 398 225–240. 10.1002/(SICI)1096-9861(19980824)398:2<225::AID-CNE5>3.0.CO;2-2 9700568

[B73] Star E. N. Kwiatkowski D. J. Murthy V. N. (2002). Rapid turnover of actin in dendritic spines and its regulation by activity. *Nat. Neurosci.* 5 239–246. 10.1038/nn811 11850630

[B74] Stiess M. Bradke F. (2011). Neuronal polarization: The cytoskeleton leads the way. *Dev. Neurobiol.* 71 430–444. 10.1002/dneu.20849 21557499

[B75] Terrian V. M. Johnston D. Clairborne B. J. Ansh-Yiadom R. Strittmatter W. J. Rea M. A. (1988). Glutamate and dynorphin release from a subcellular fraction enriched in hippocampal mossy fiber synaptosomes. *Brain Res. Bull.* 21 343–351. 10.1016/0361-9230(88)90146-3 2905627

[B76] Tønnesen J. Nägerl U. V. (2016). Dendritic spines as tunable regulators of synaptic signals. *Front. Psychiatry* 7:101. 10.3389/fpsyt.2016.00101 27340393 PMC4899469

[B77] Toth A. Boczan J. Kedei N. Lizanecz E. Bagi Z. Papp Z. (2005). Expression and distribution of vanilloid receptor 1 (TRPV1) in the adult rat brain. *Brain Res. Mol. Brain Res.* 135 162–168. 10.1016/j.molbrainres.2004.12.003 15857679

[B78] Tsay D. Yuste R. (2004). On the electrical function of dendritic spines. *Trends Neurosci.* 27 77–83. 10.1016/j.tins.2003.11.008 15102486

[B79] von Bohlen Und Halbach O. (2009). Structure and function of dendritic spines within the hippocampus. *Ann. Anat.* 191 518–531. 10.1016/j.aanat.2009.08.006 19783417

[B80] Wyszynski M. Lin J. Rao A. Nigh E. Beggs A. H. Craig A. M. (1997). Competitive binding of α-actinin and calmodulin to the NMDA receptor. *Nature* 385 439–442. 10.1038/385439a0 9009191

[B81] Yuste R. Majewska A. (2001). On the function of dendritic spines. *Neuroscientist* 7 387–395. 10.1177/107385840100700508 11597098

[B82] Yuste R. Majewska A. Cash S. S. Denk W. (1999). Mechanisms of calcium influx into spines: Heterogeneity among spines, coincidence detection by NMDA receptors and optical quantal analysis. *J. Neurosci.* 19 1976–1987. 10.1523/JNEUROSCI.19-06-01976.1999 10066251 PMC6782554

[B83] Zha X. M. Wemmie J. A. Green S. H. Welsh M. J. (2006). Acid-sensing ion channel 1a is a postsynaptic proton receptor that affects the density of dendritic spines. *Proc. Natl. Acad. Sci. U.S.A.* 103 16556–16561. 10.1073/pnas.0608018103 17060608 PMC1621052

[B84] Zito K. Knott G. Shepherd G. M. G. Shenolikar S. Svoboda K. (2014). Induction of spine growth and synapse formation by regulation of the spine actin cytoskeleton. *Neuron* 44 321–334. 10.1016/j.neuron.2004.09.022 15473970

