# Supplementary figures and images for: Electrical recordings from dendritic spines of adult mouse hippocampus and effect of the actin cytoskeleton
Source: Front Mol Neurosci. 2022 Aug 25;15:769725. doi: 10.3389/fnmol.2022.769725 (PMC9453158; doi:10.3389/fnmol.2022.769725)

# PSD95

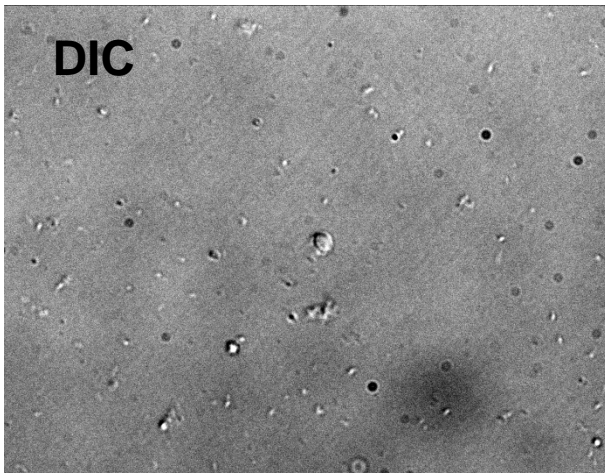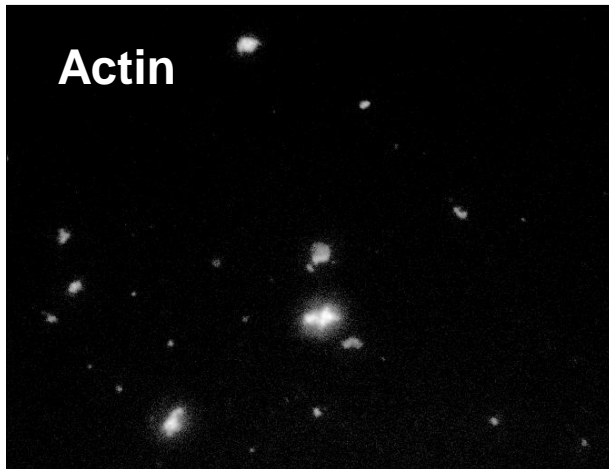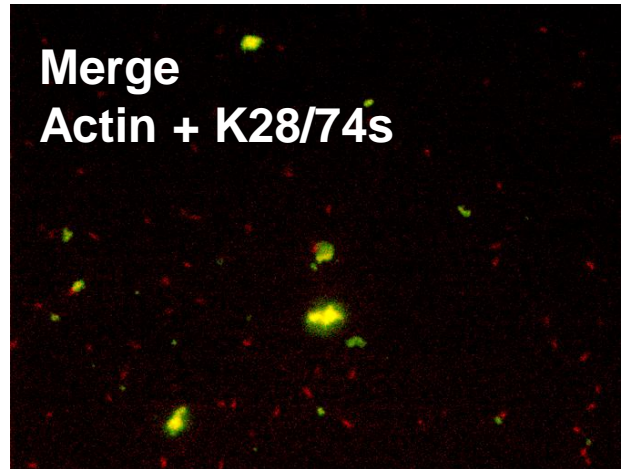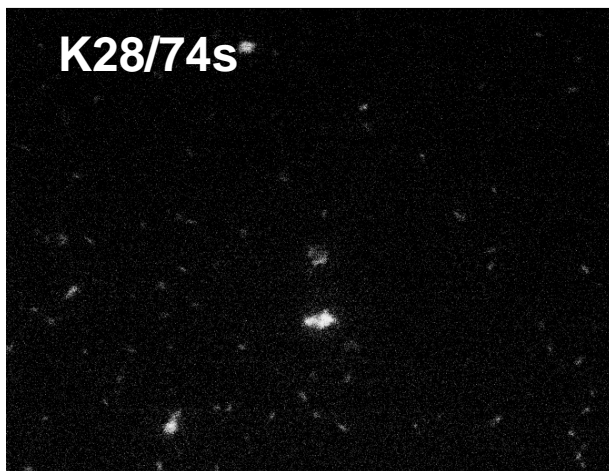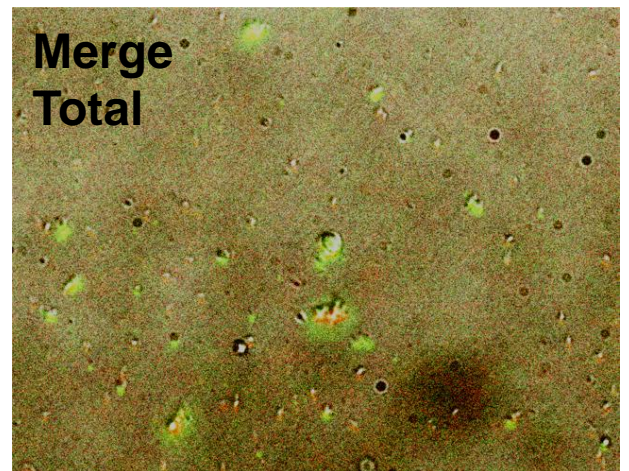

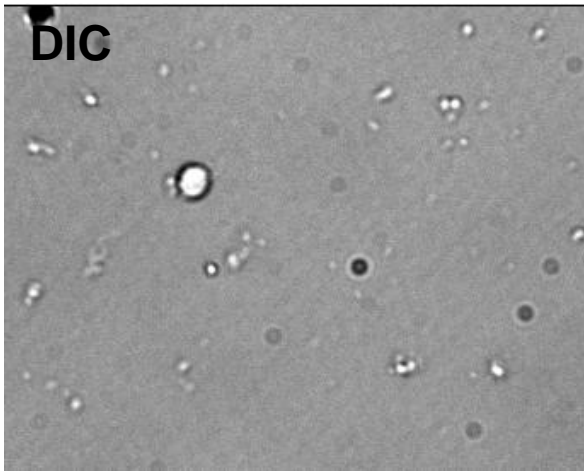

# Glutamate Receptor Ionotropic, NMDA2A

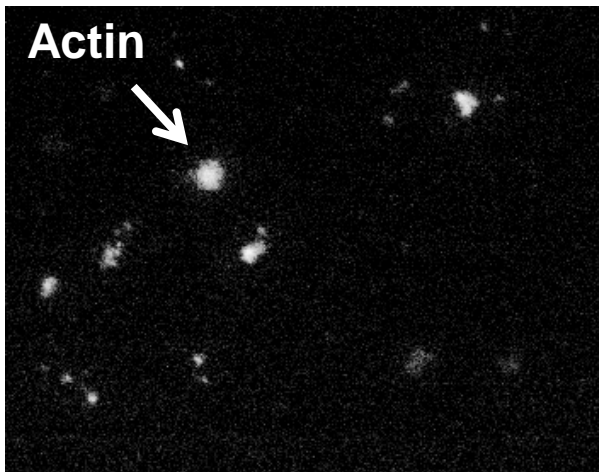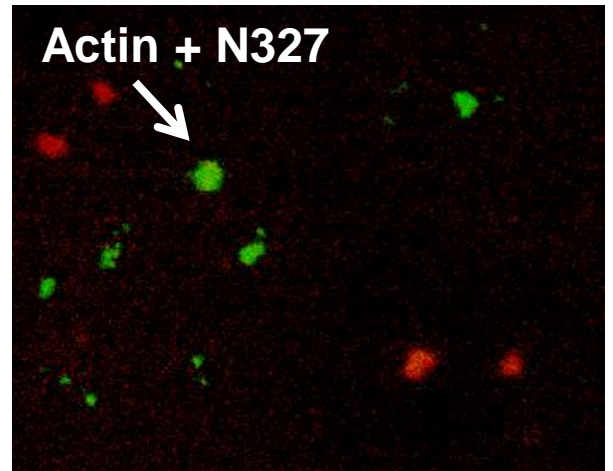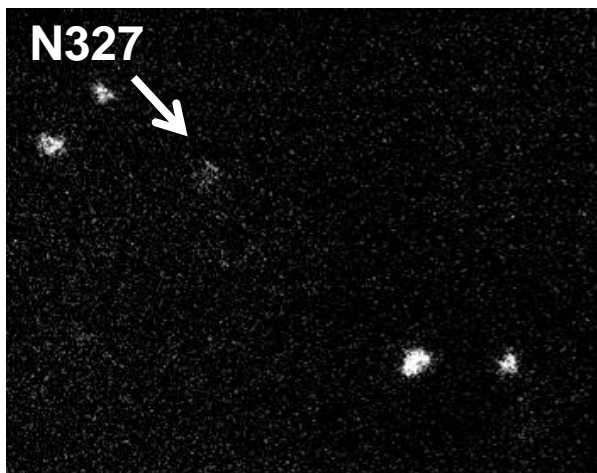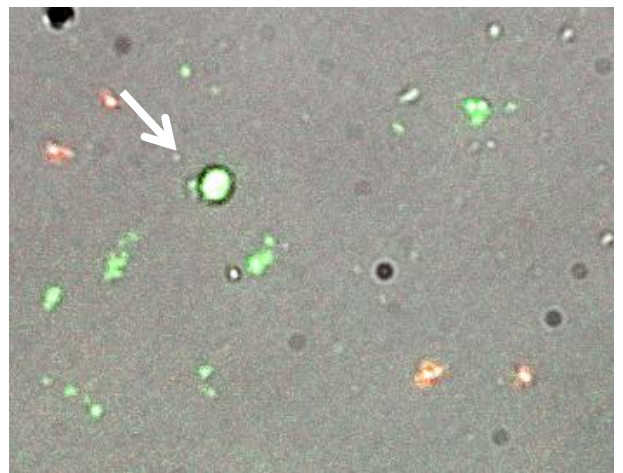

Supplement: Supplementary file 1 [file Data_Sheet_1.PDF]
