# Supplementary material for: Electrical recordings from dendritic spines of adult mouse hippocampus and effect of the actin cytoskeleton
Source: Front Mol Neurosci. 2022 Aug 25;15:769725. doi: 10.3389/fnmol.2022.769725 (PMC9453158; doi:10.3389/fnmol.2022.769725)
Supplement: Supplementary file 2 [file Data_Sheet_2.pdf]

Whole supernantant - Sample 1

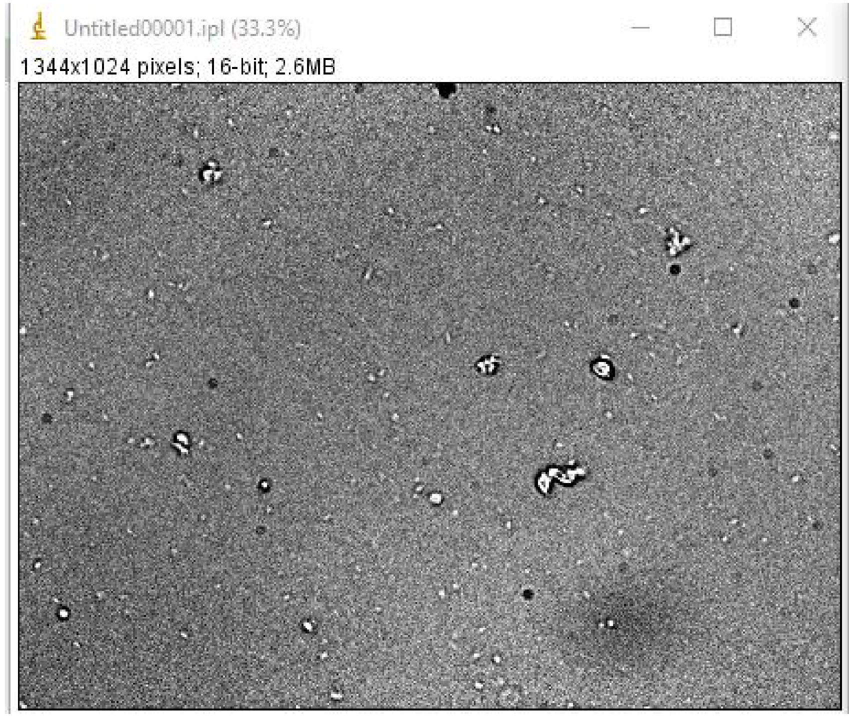

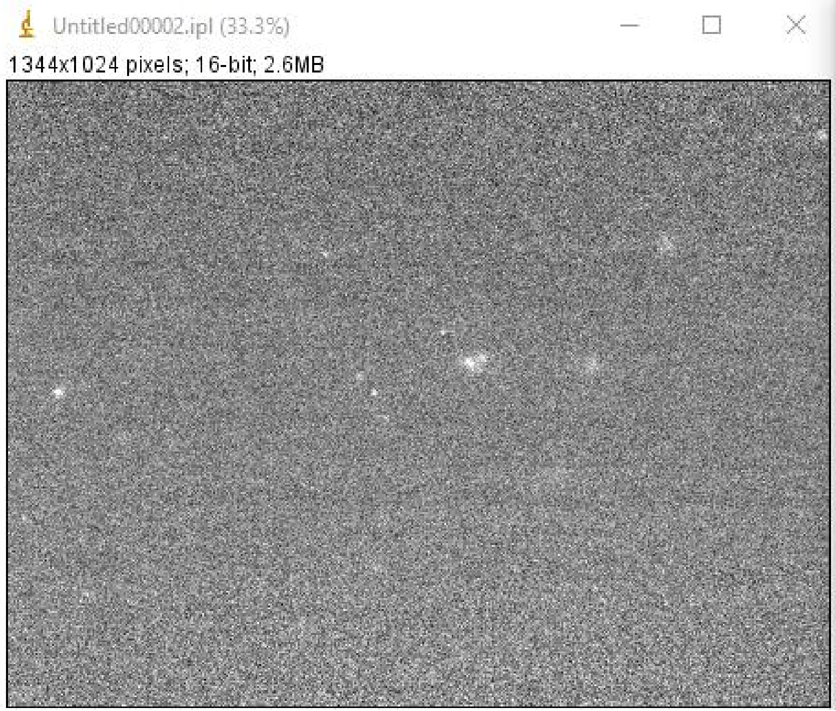

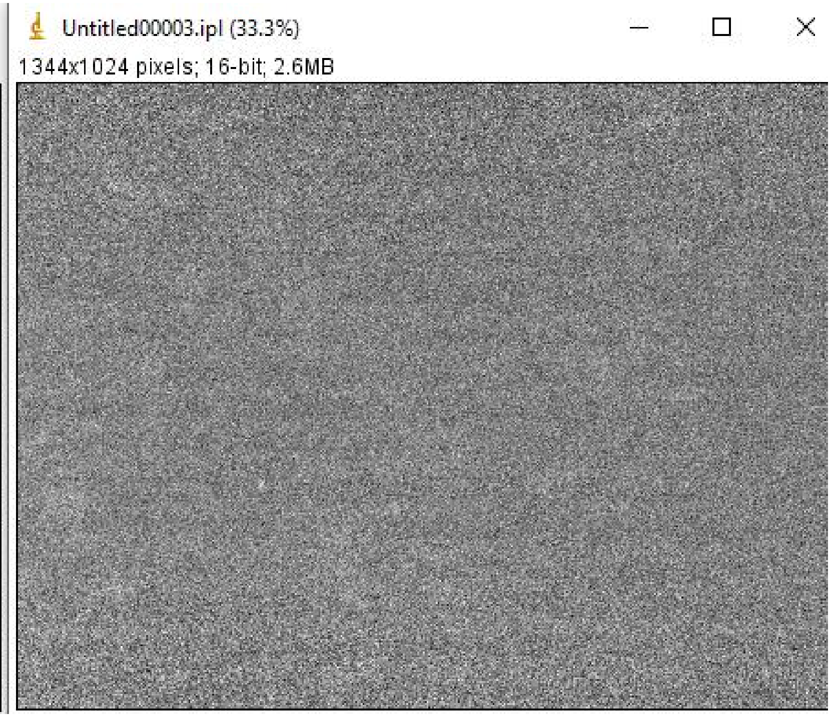

Whole supernantant - Sample 1

21 marks (FITC)

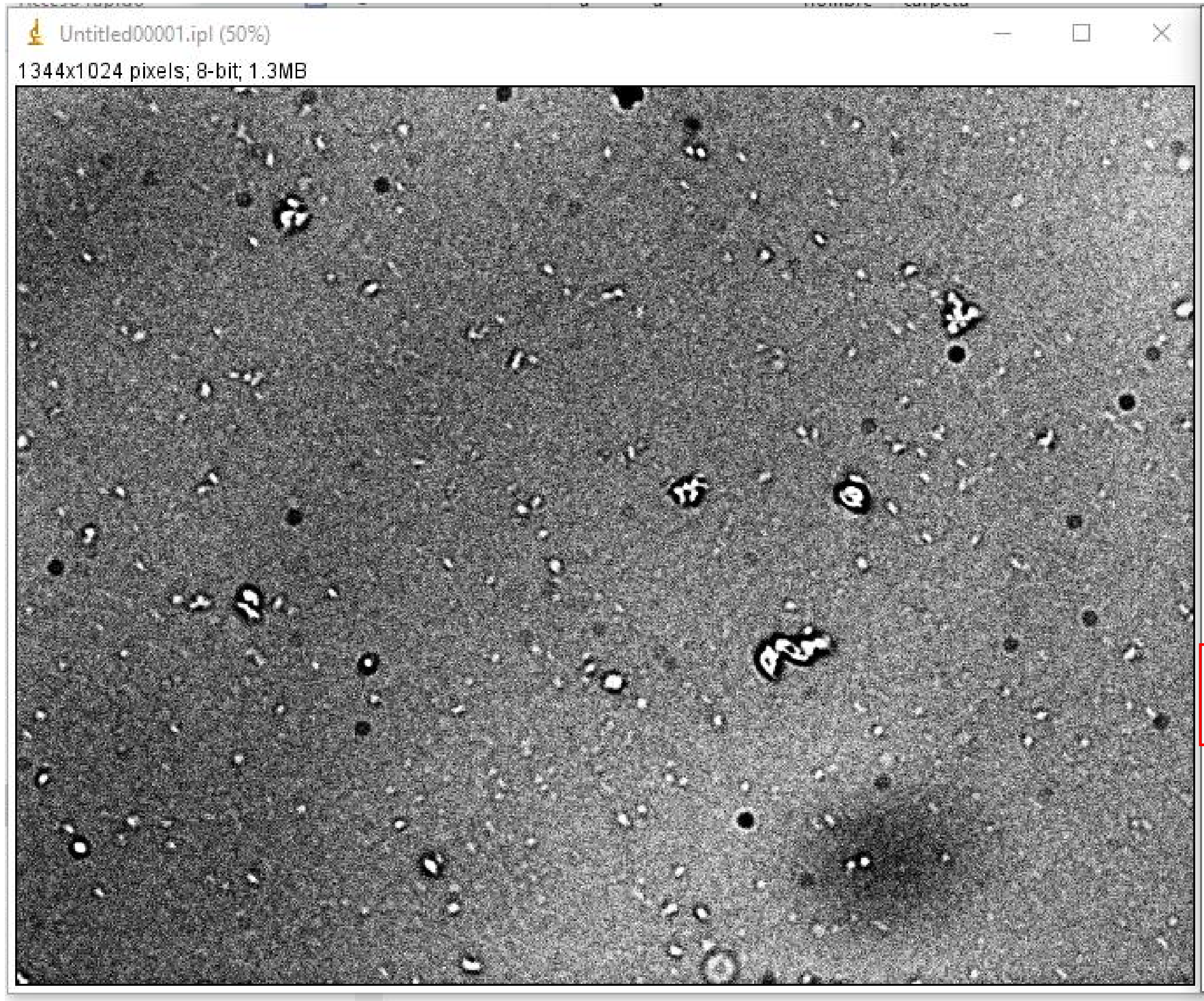

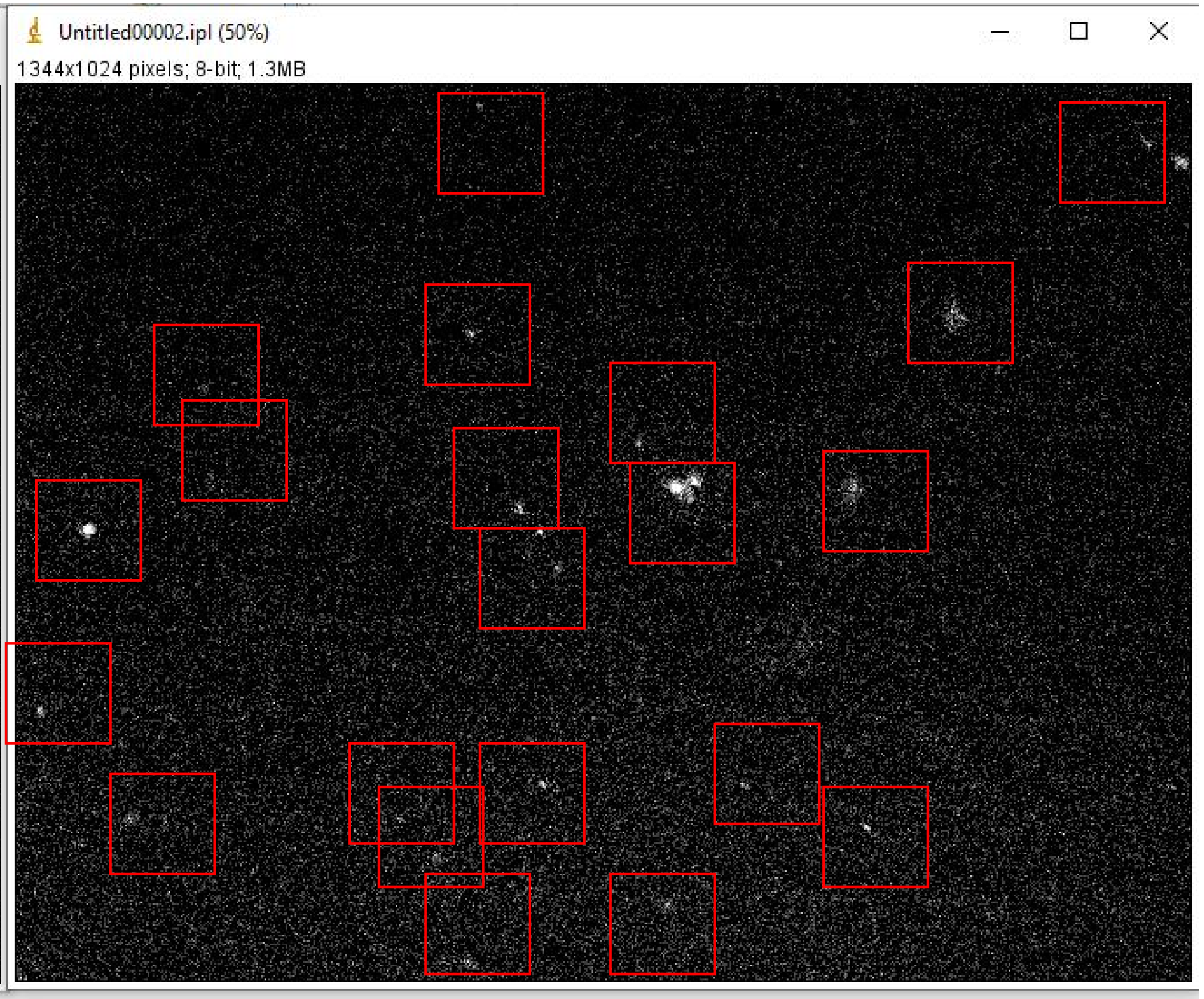

Whole supernantant - Sample 1

13 marks (TxR)

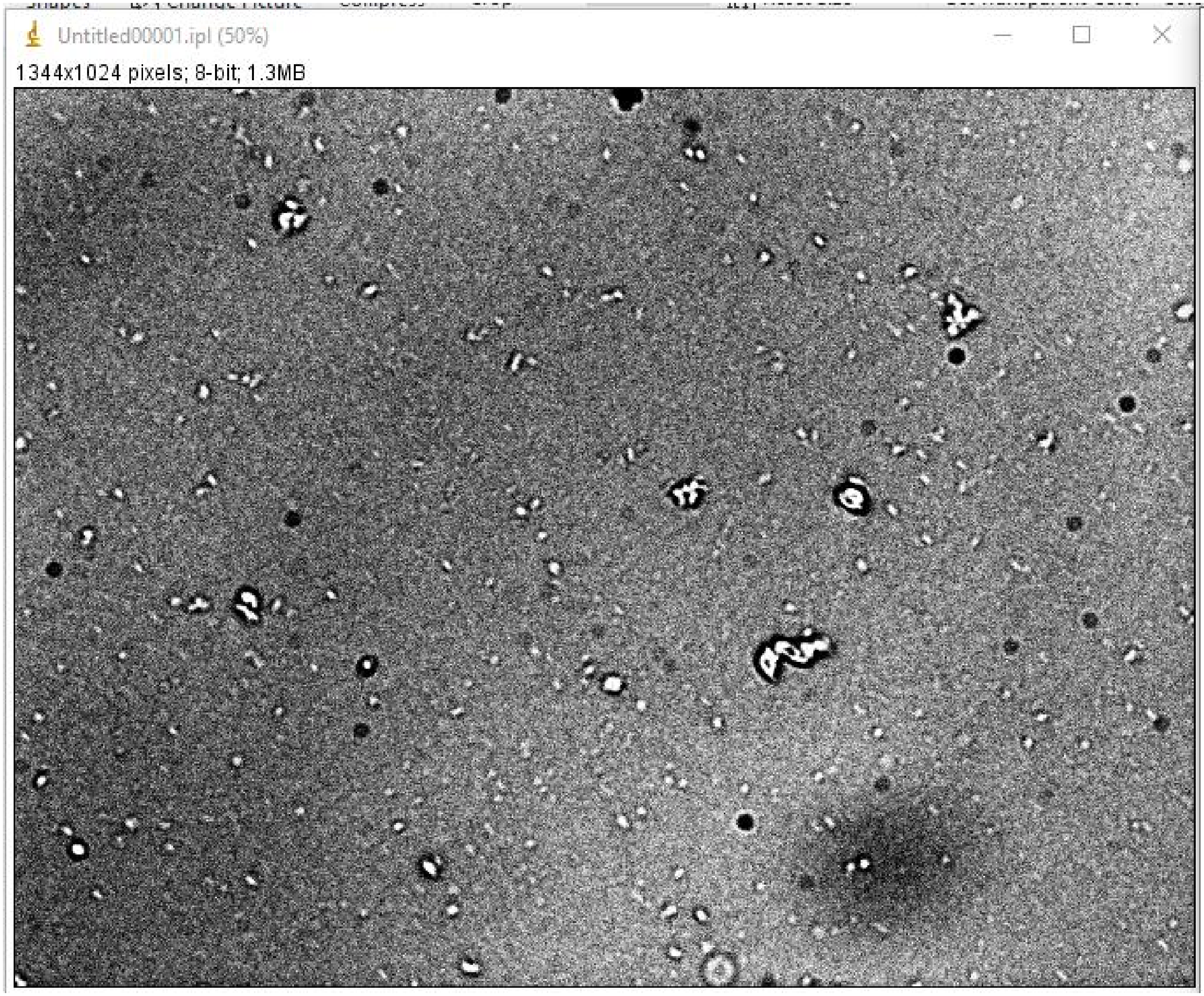

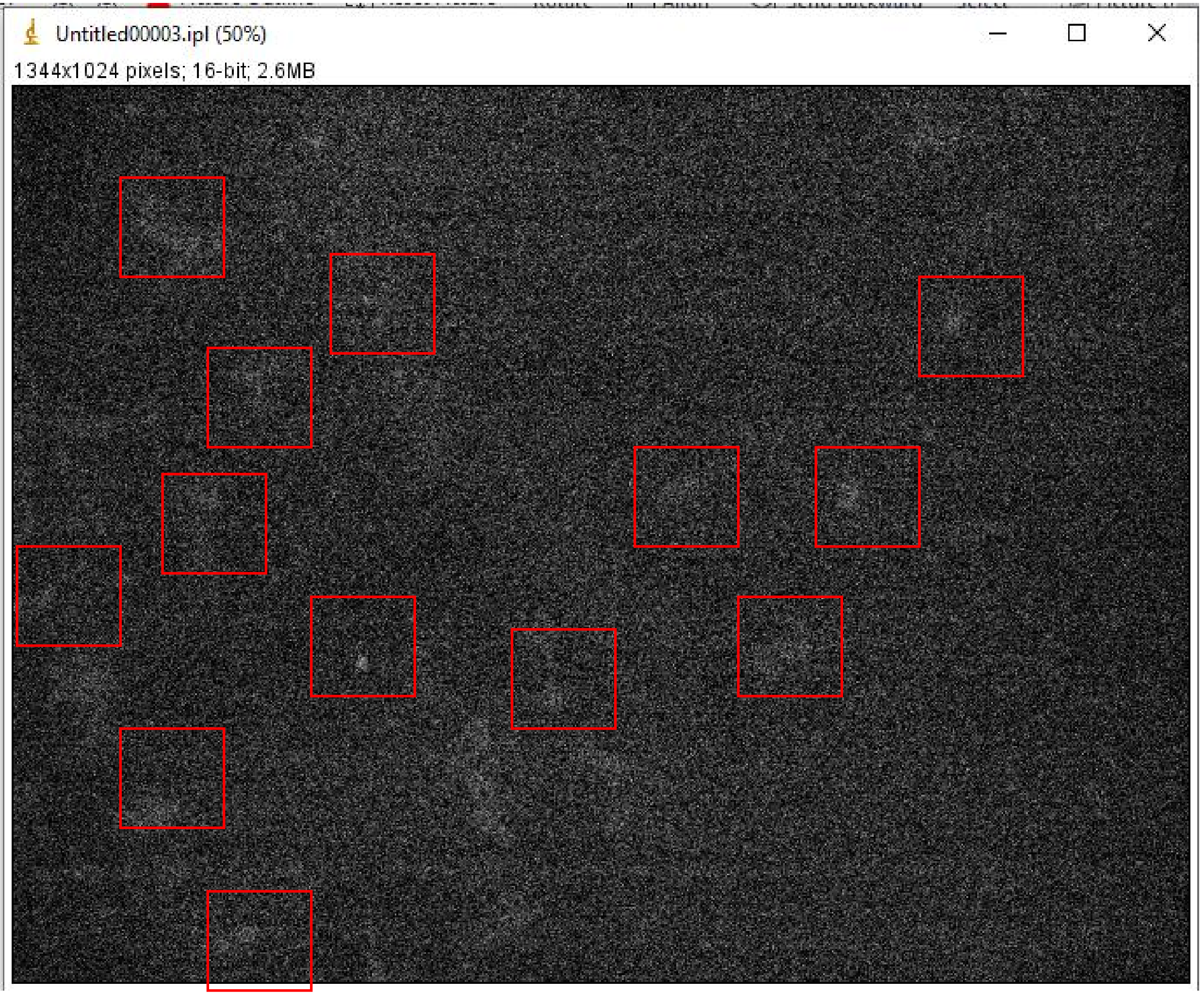

Whole supernantant - Sample 1

1 round mark

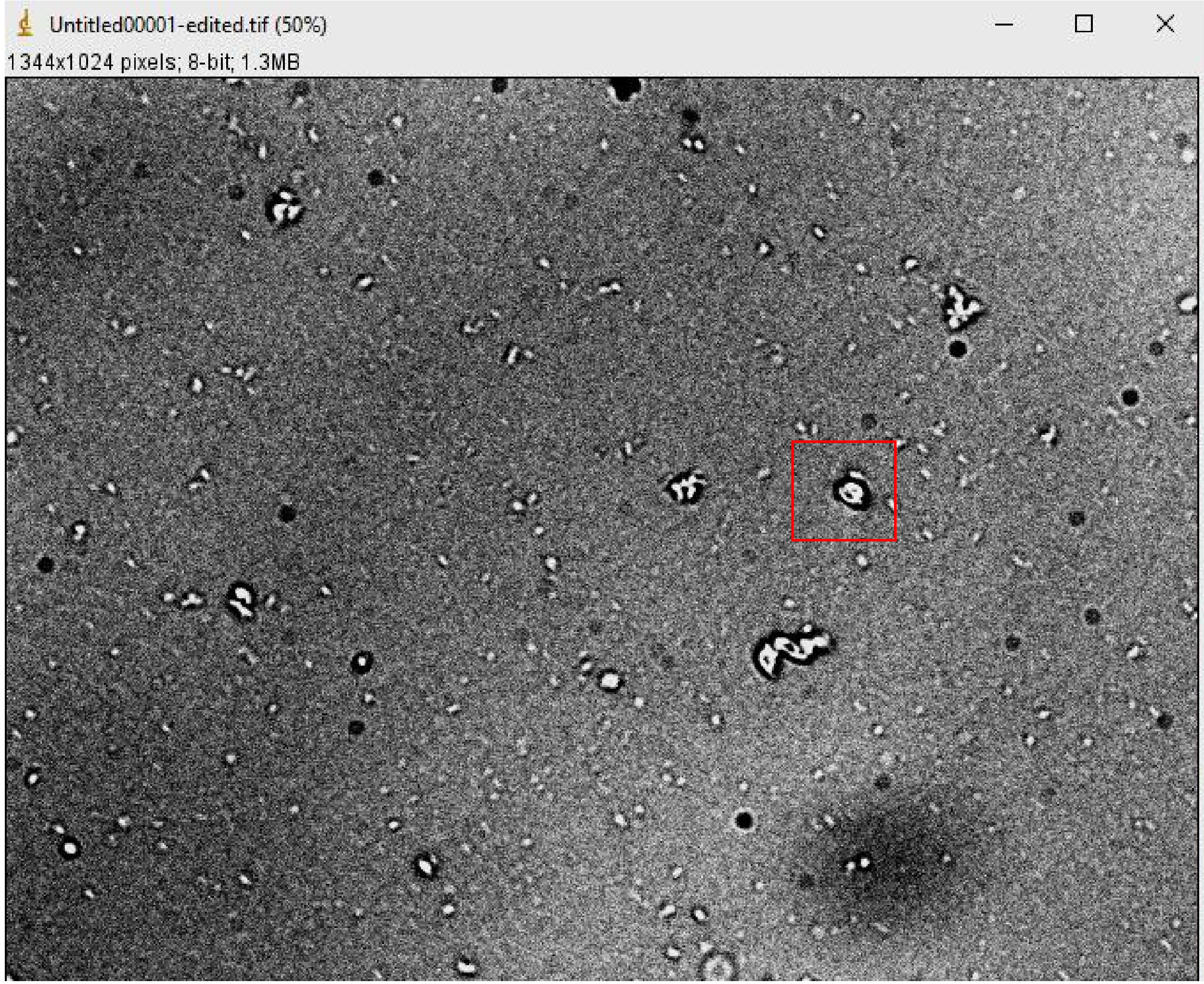

1 DIC + FITC + TxR merge

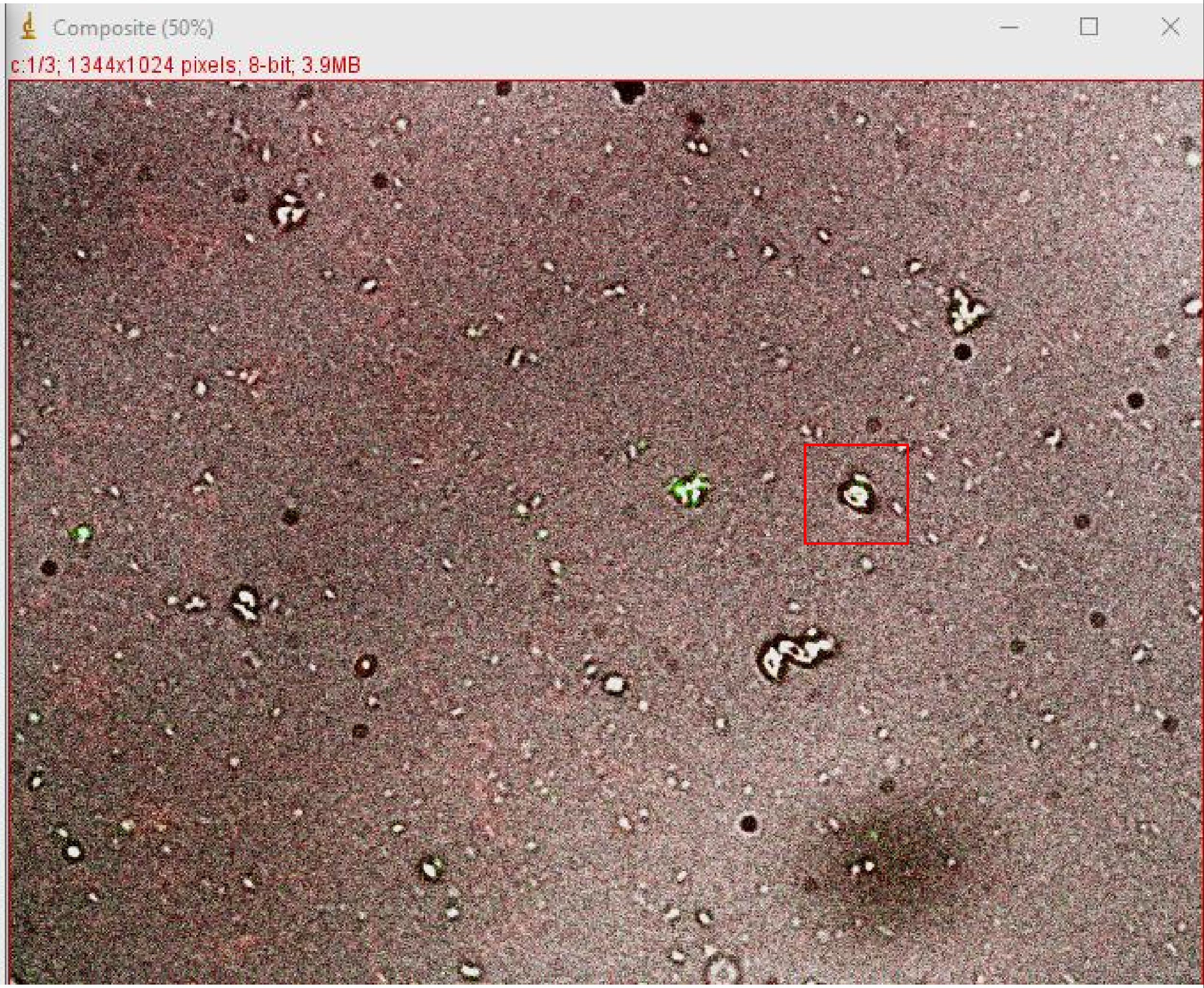

Whole supernantant - Sample 1

$$Ratio = \frac{1}{(13 + 21 + 1)/3} = 0.08$$

Whole supernantant - Sample 2

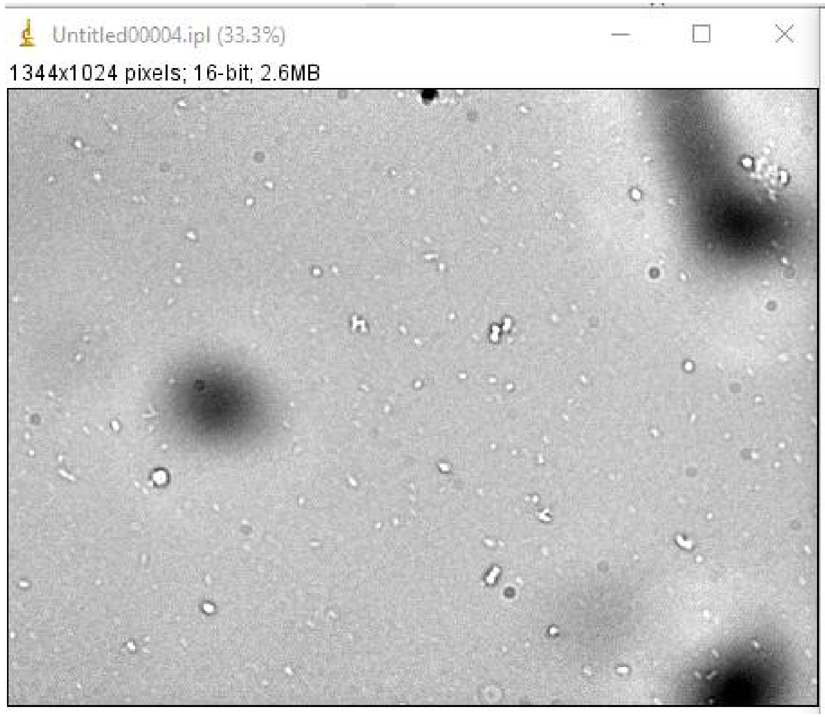

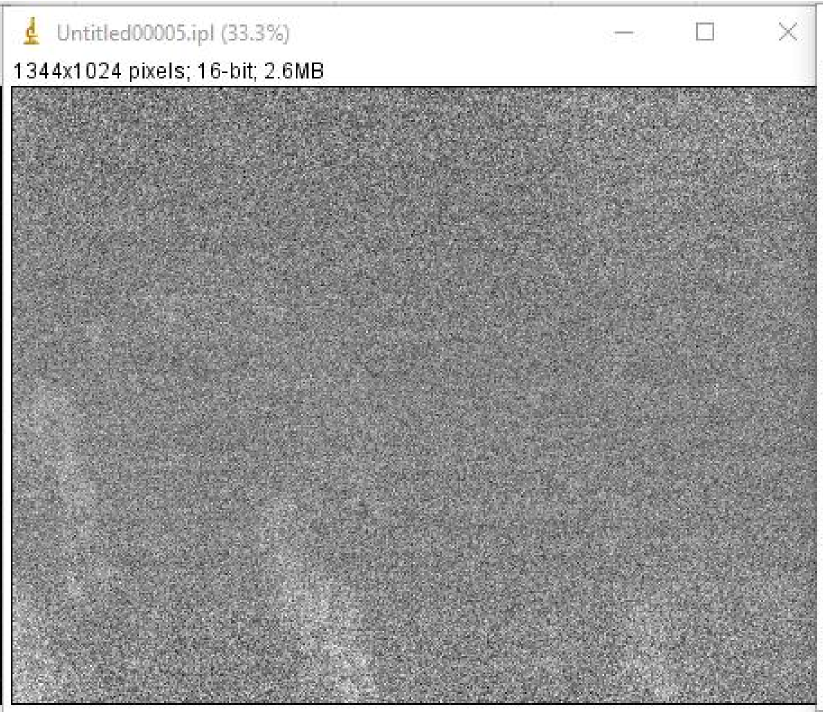

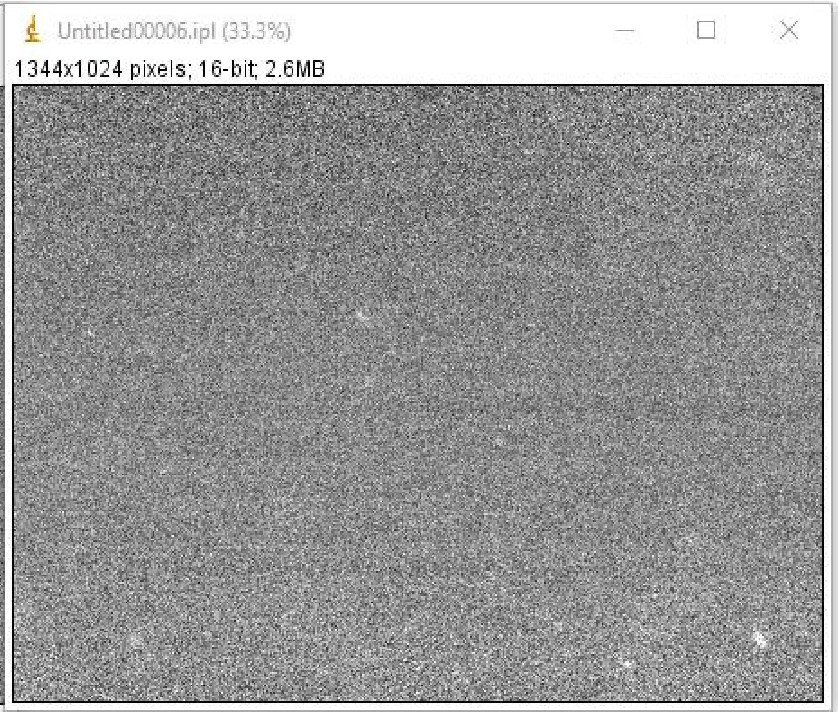

Whole supernantant - Sample 2

0 puntos TxR

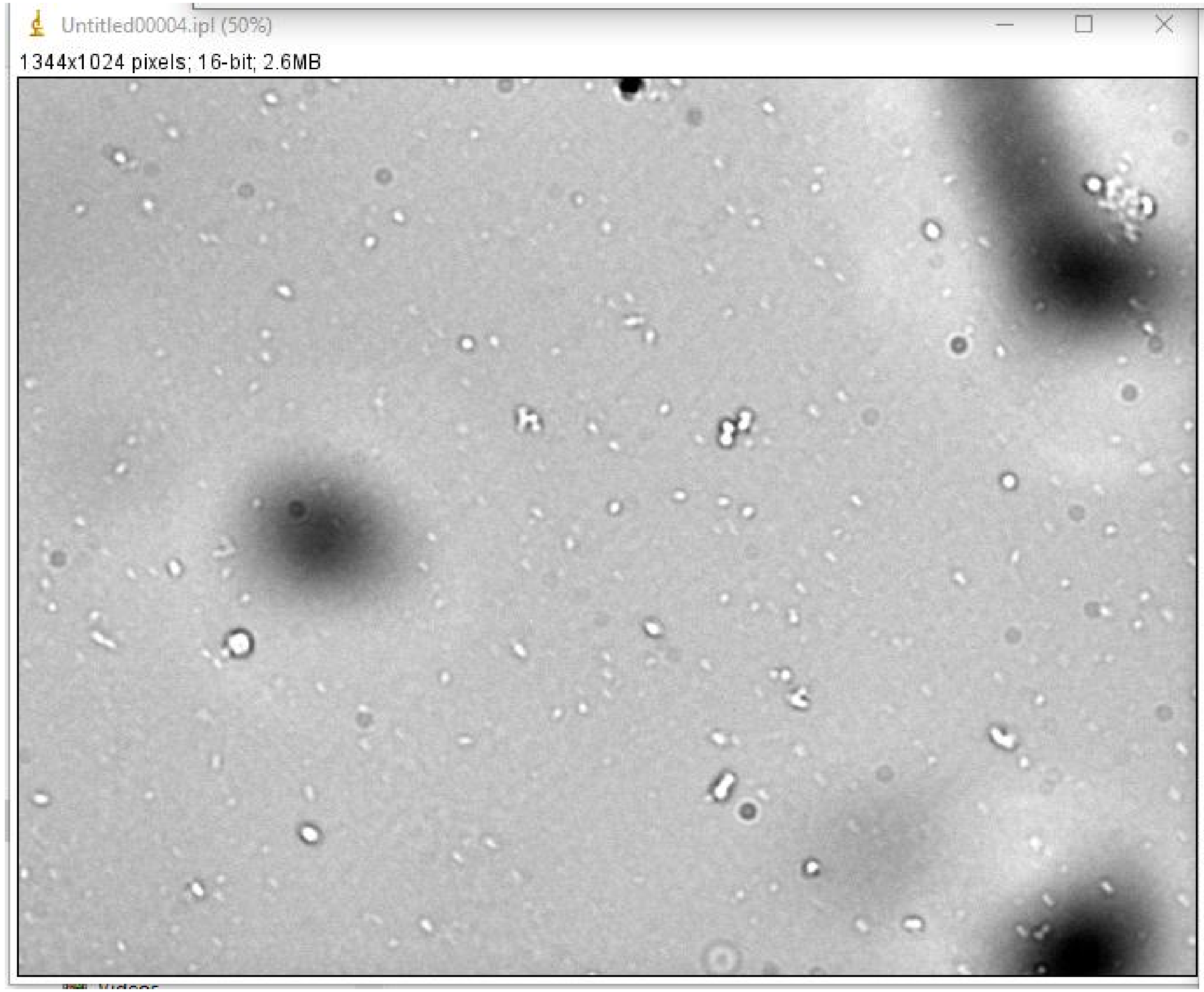

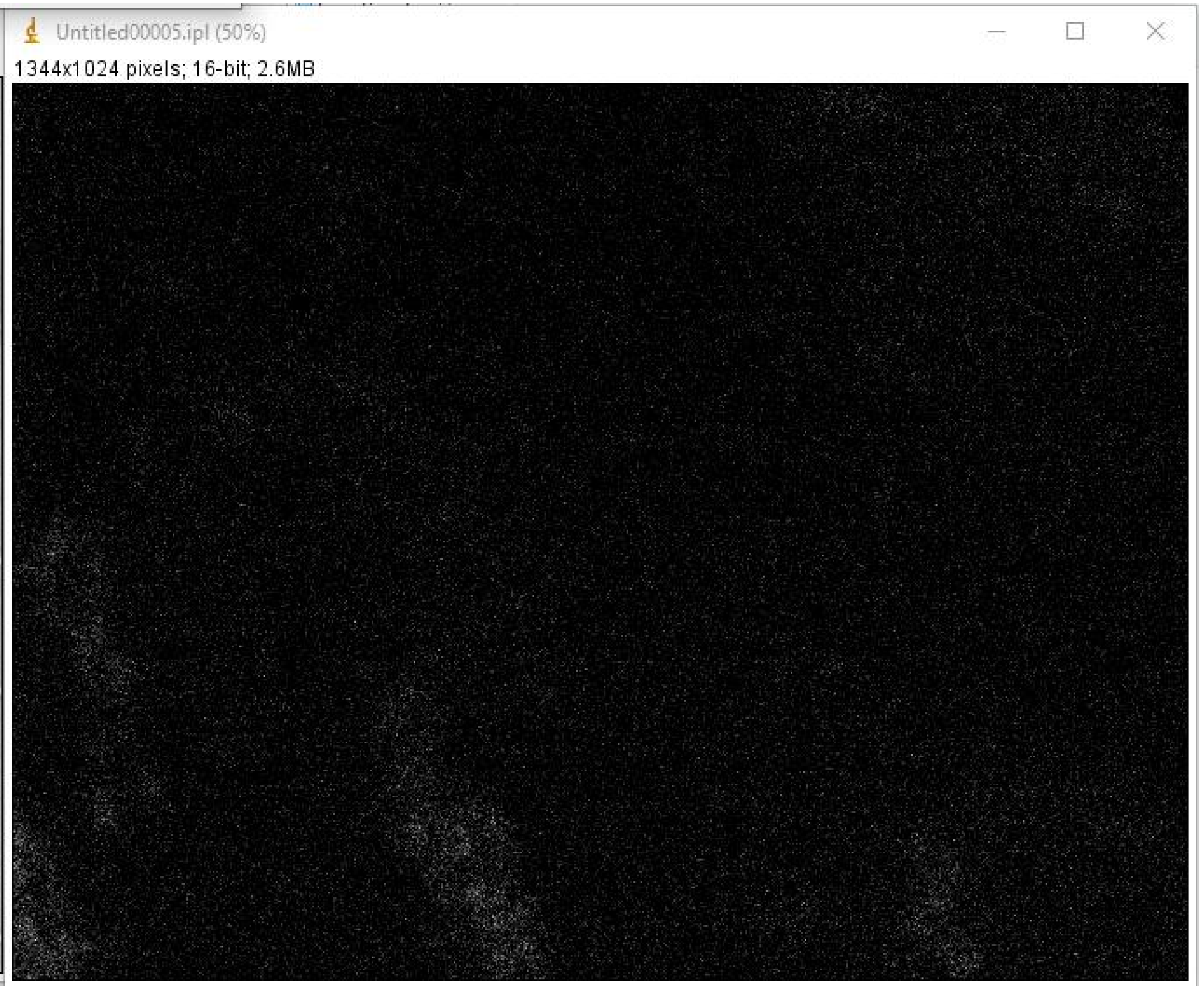

Whole supernatant - Sample 2

4 round marks

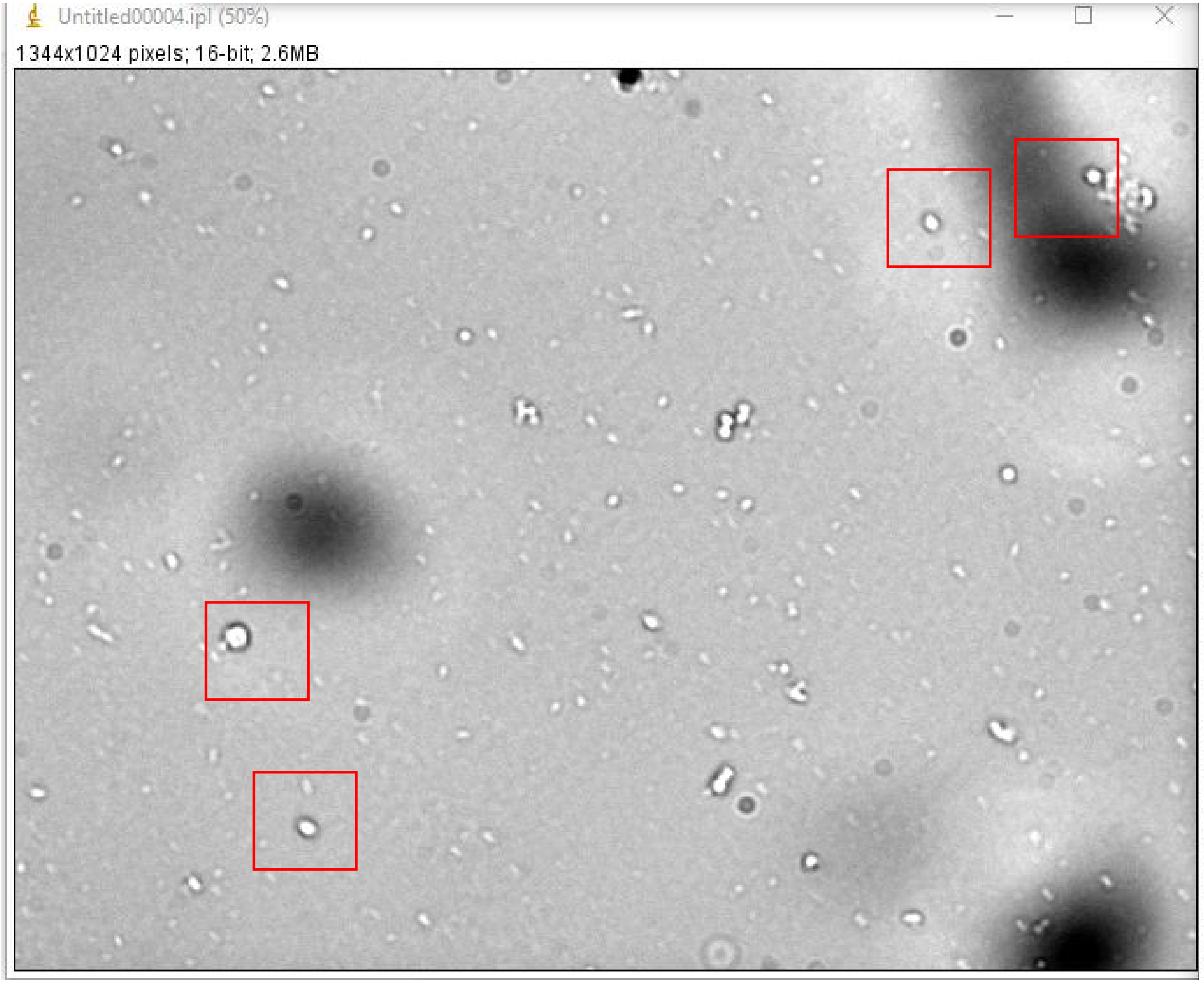

11 marks FITC

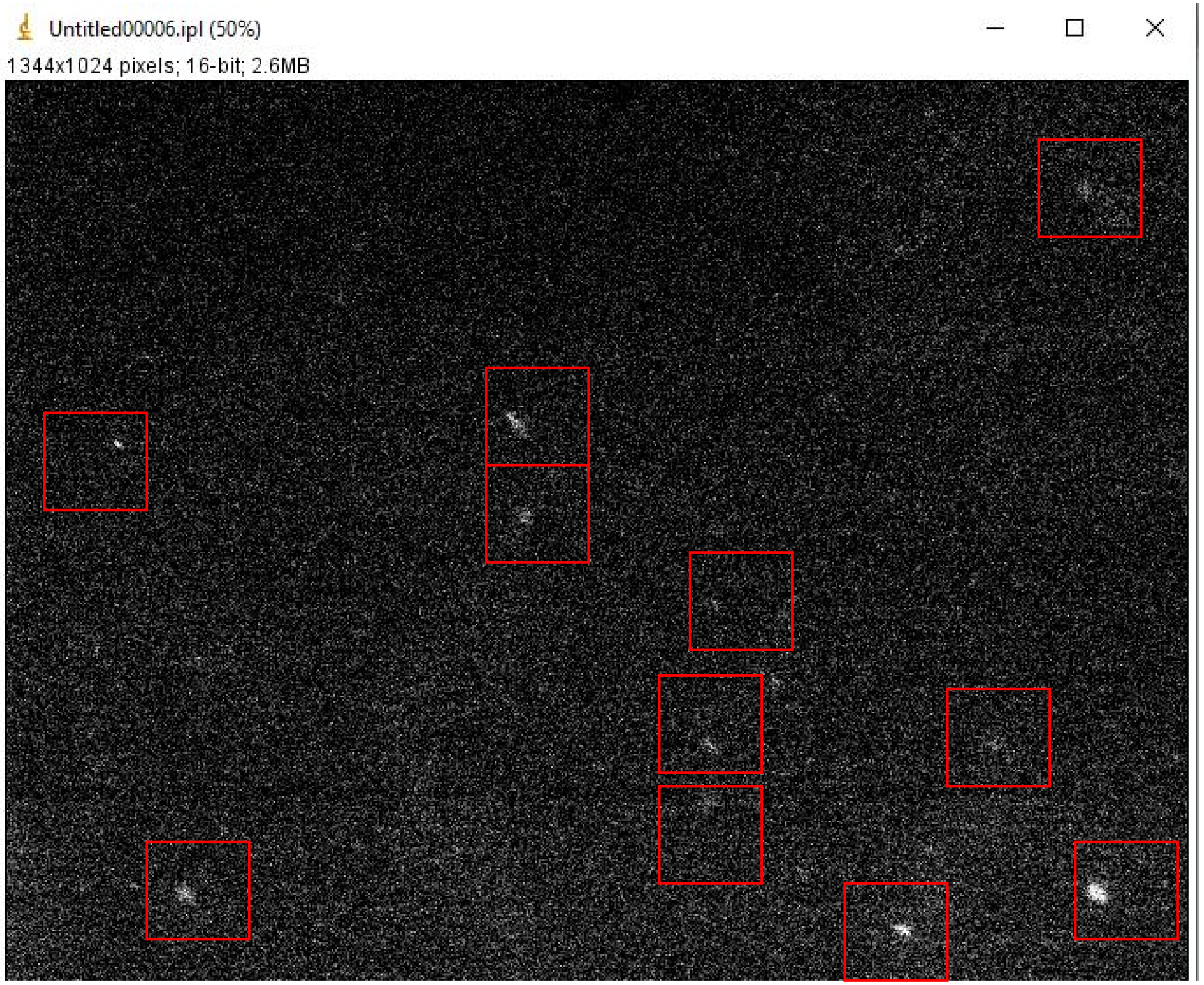

Whole supernantant - Sample 2

0 DIC + FITC + TxR merge

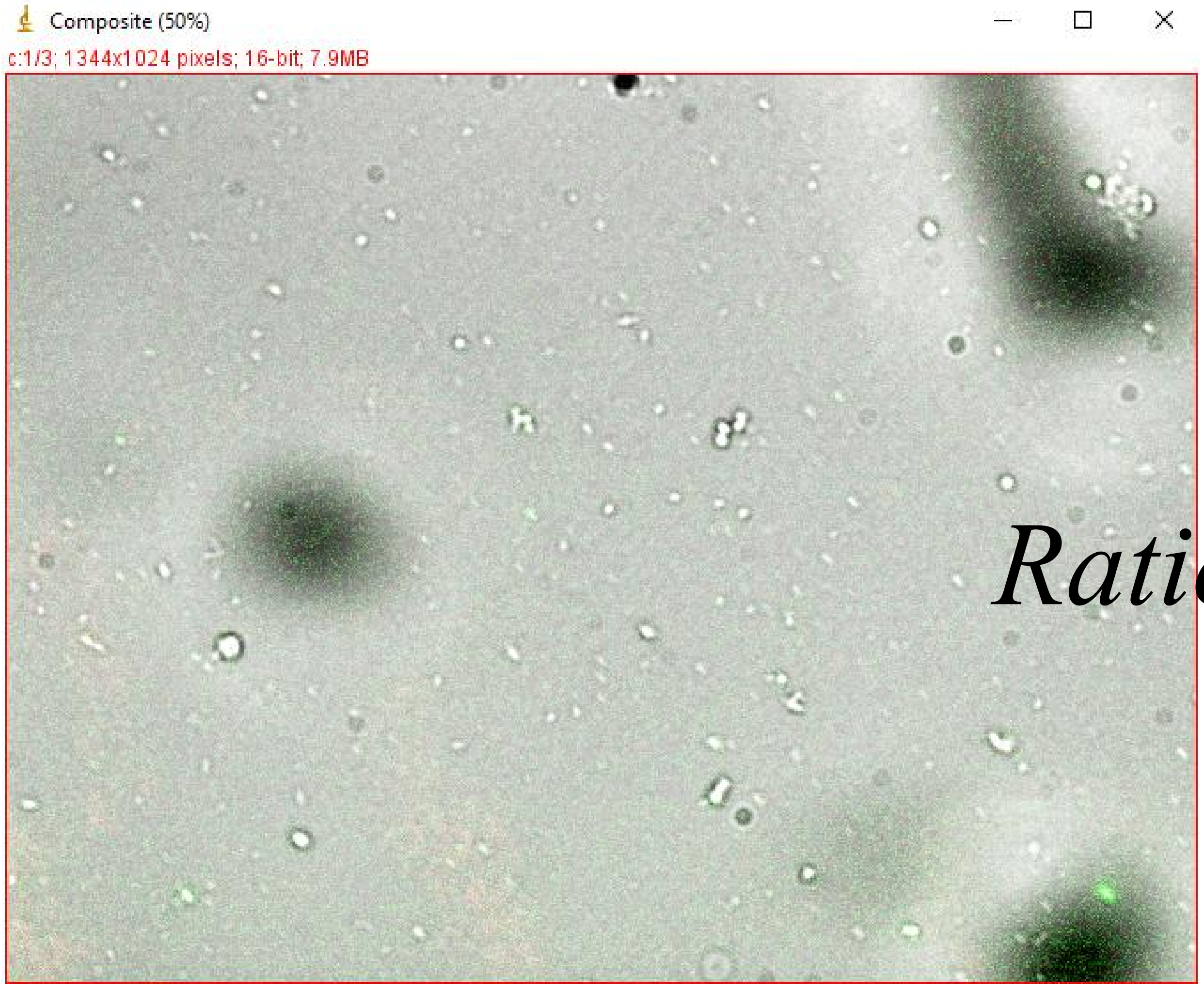

$$Ratio = \frac{0}{(11+4+0)/3} = 0$$

Whole supernantant - Sample 3

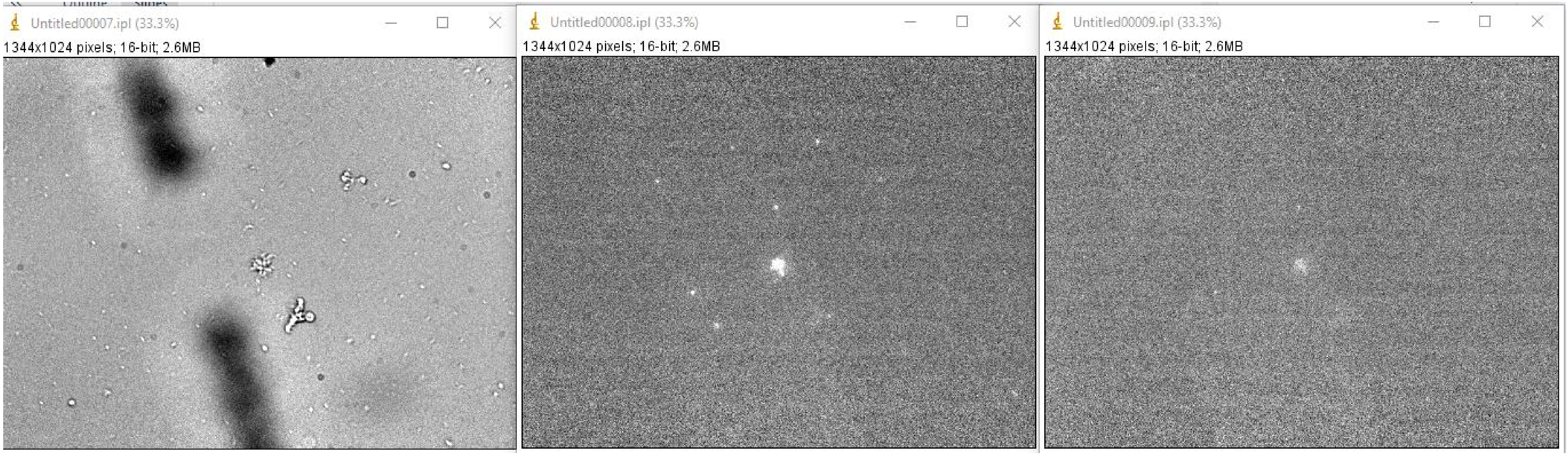

Whole supernantant - Sample 3

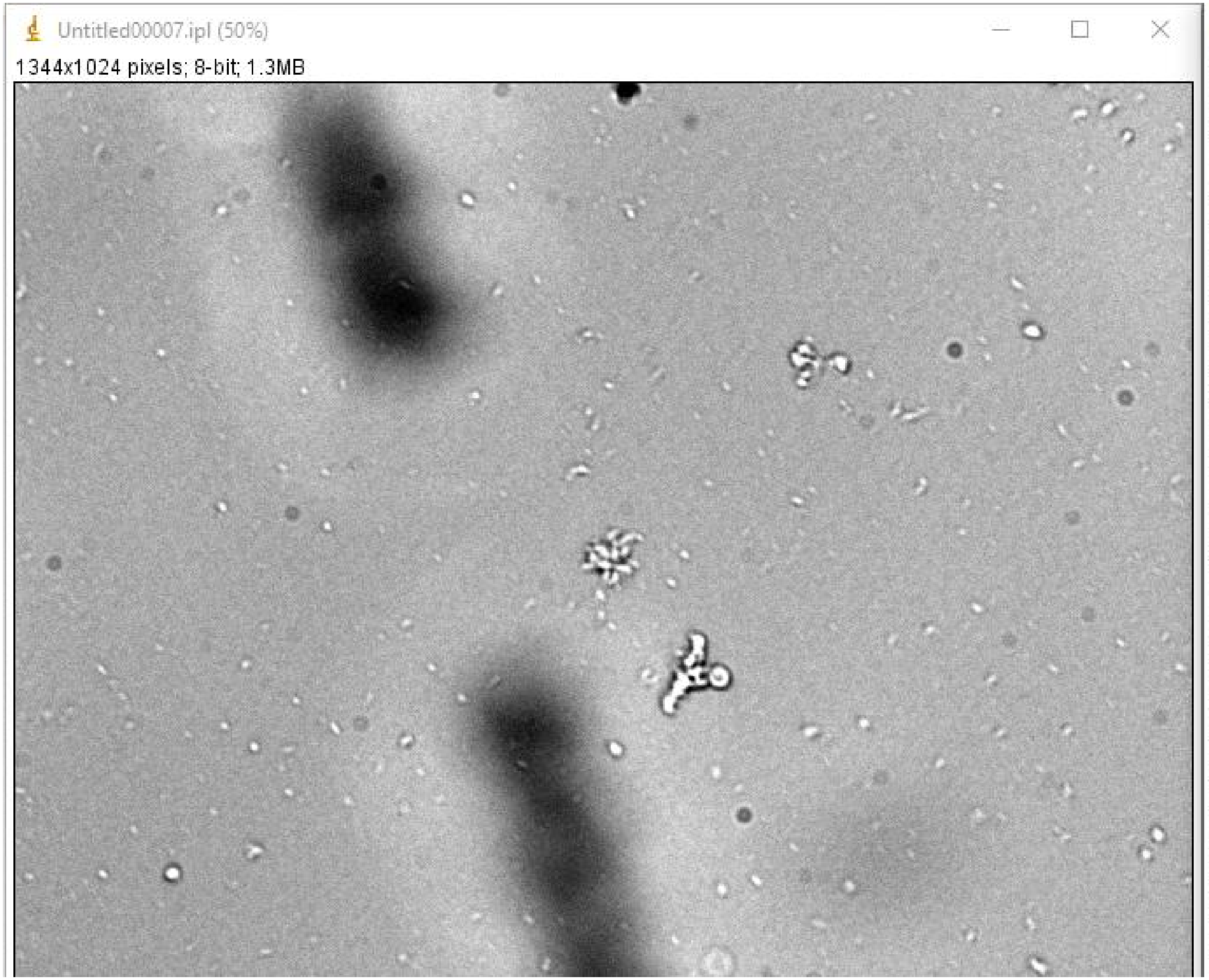

12 marks FITC

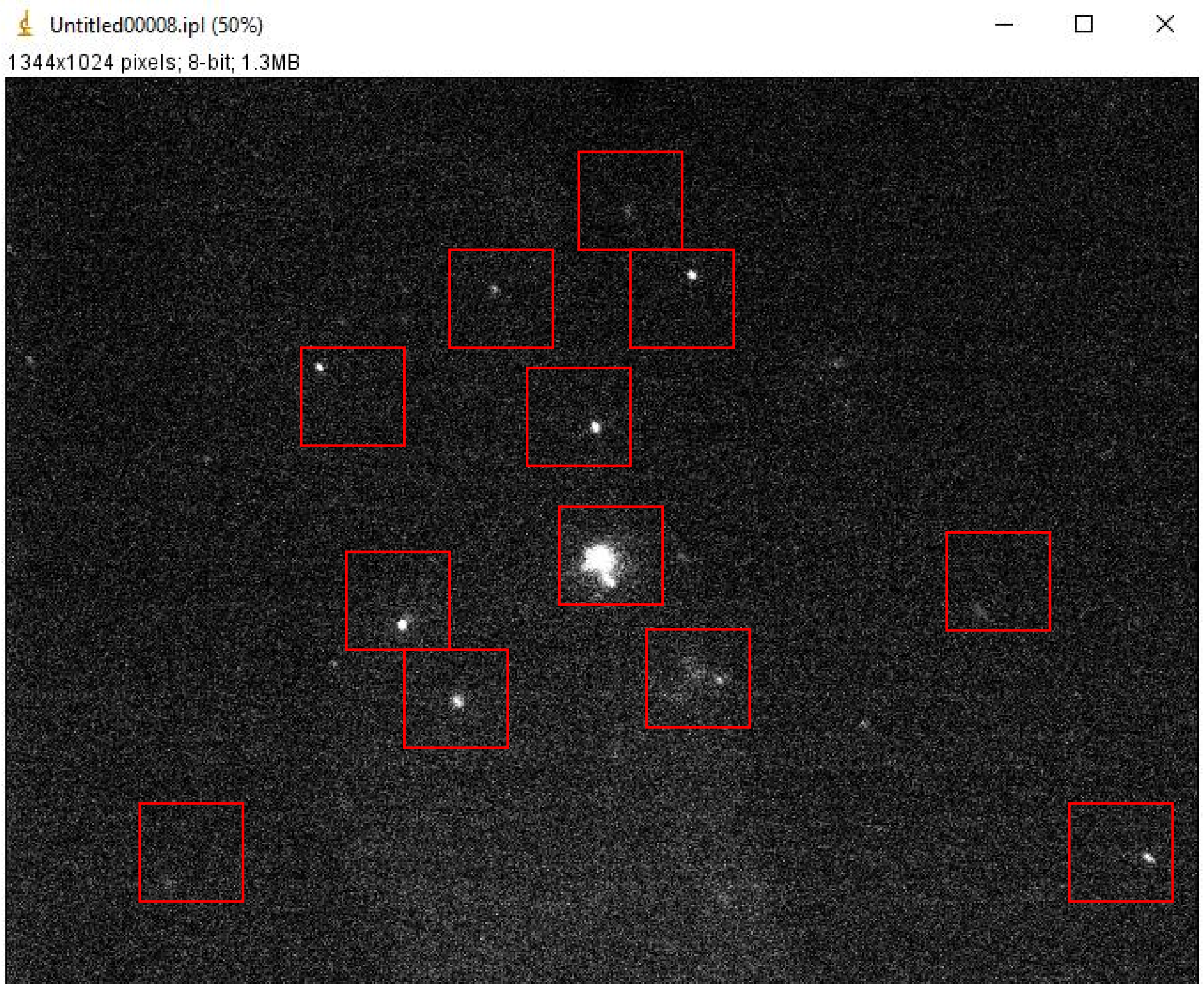

Whole supernantant - Sample 3

1 round mark DIC

3 marks TxR

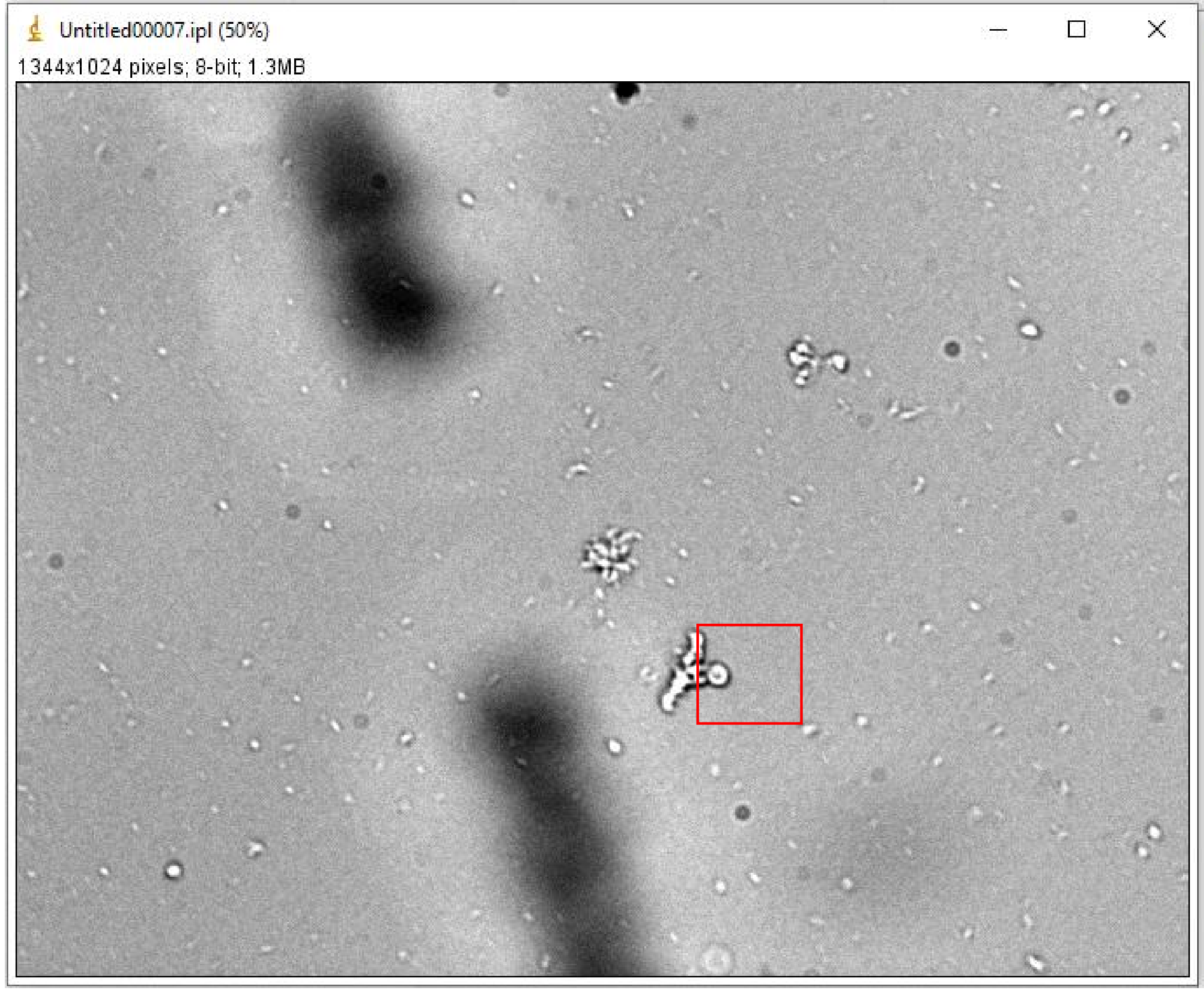

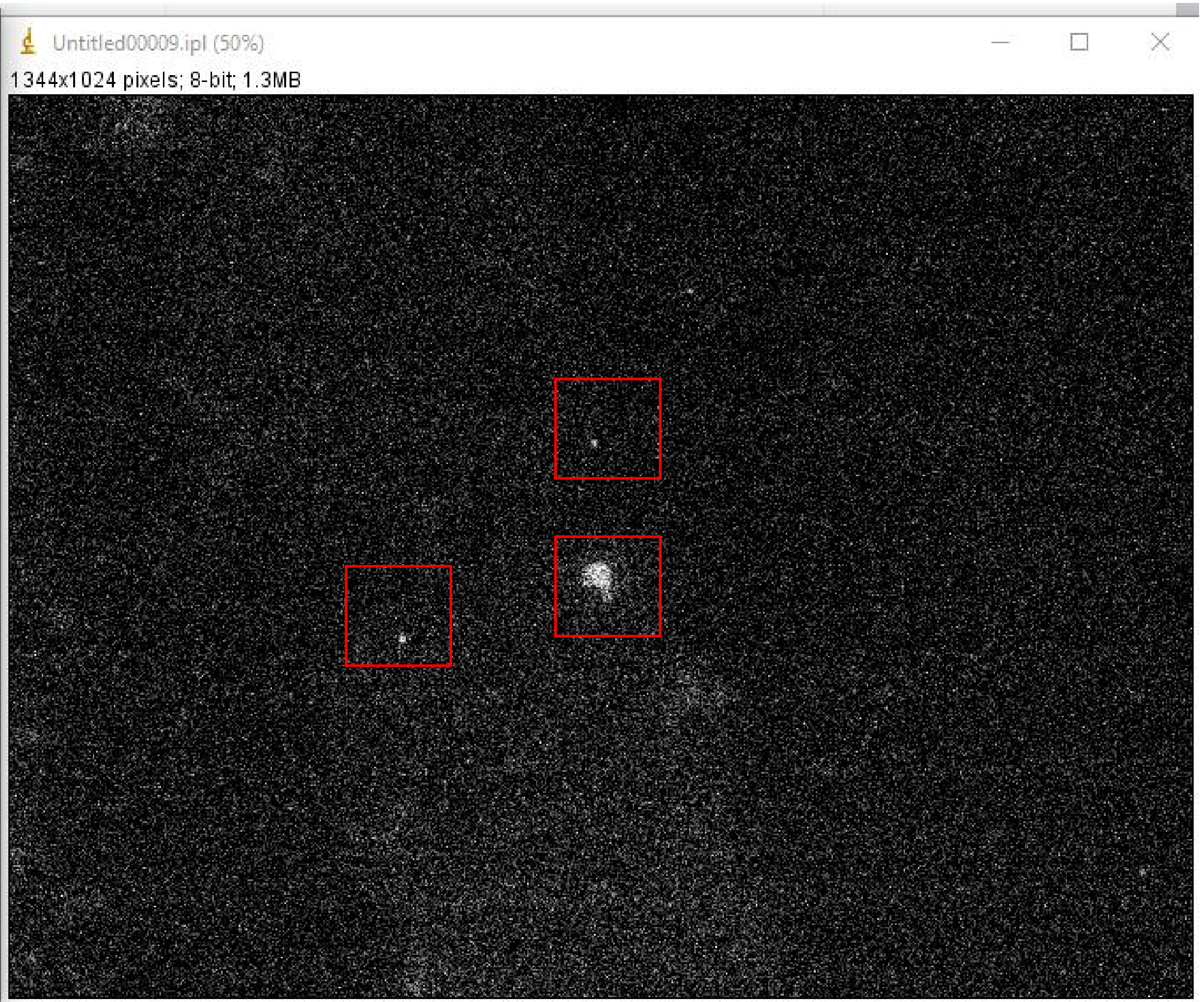

Whole supernantant - Sample 3

1 DIC + FITC + TxR merge

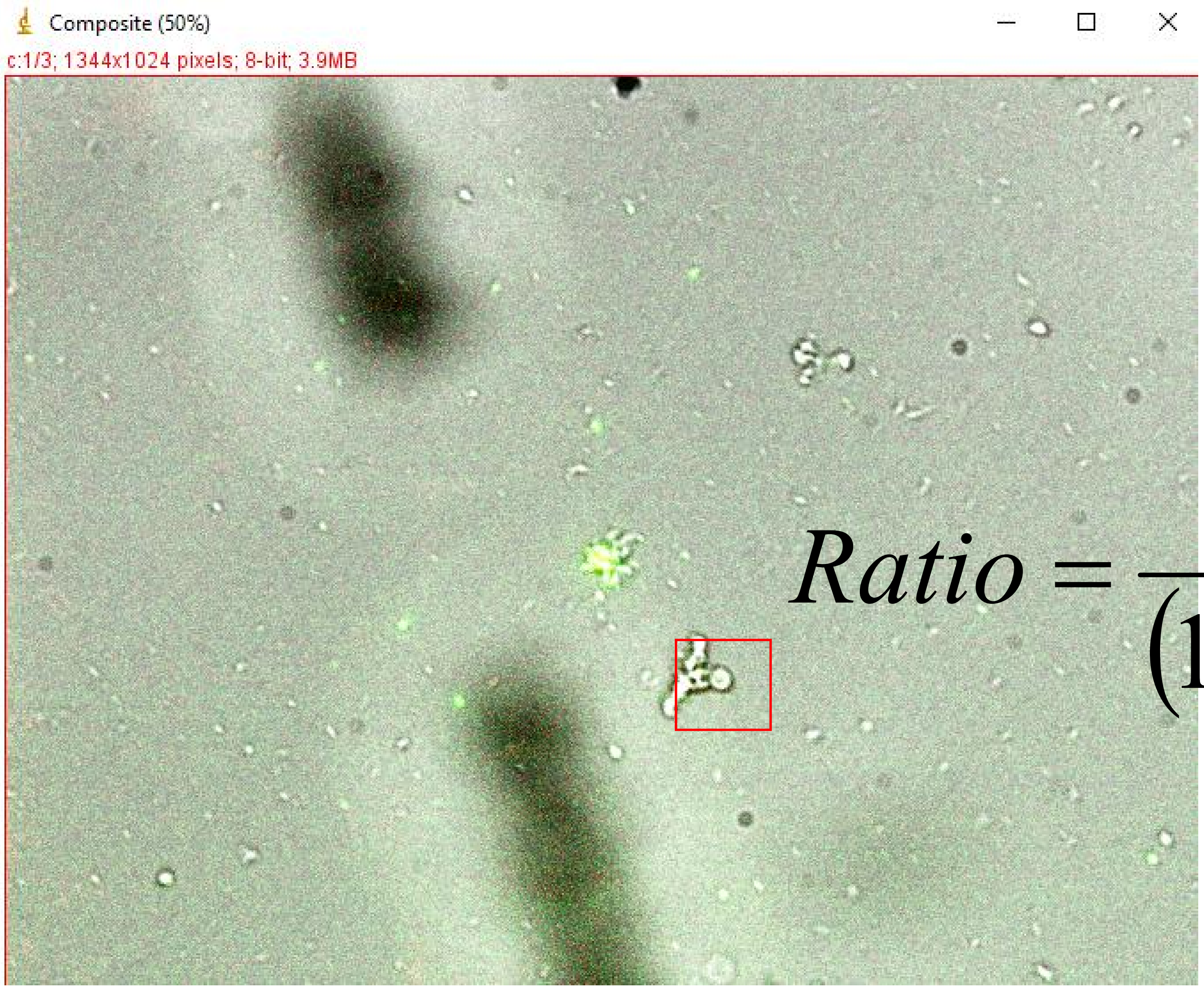

$$Ratio = \frac{1}{(12+3+1)/3} = 0.188$$

Whole supernantant - Sample 4

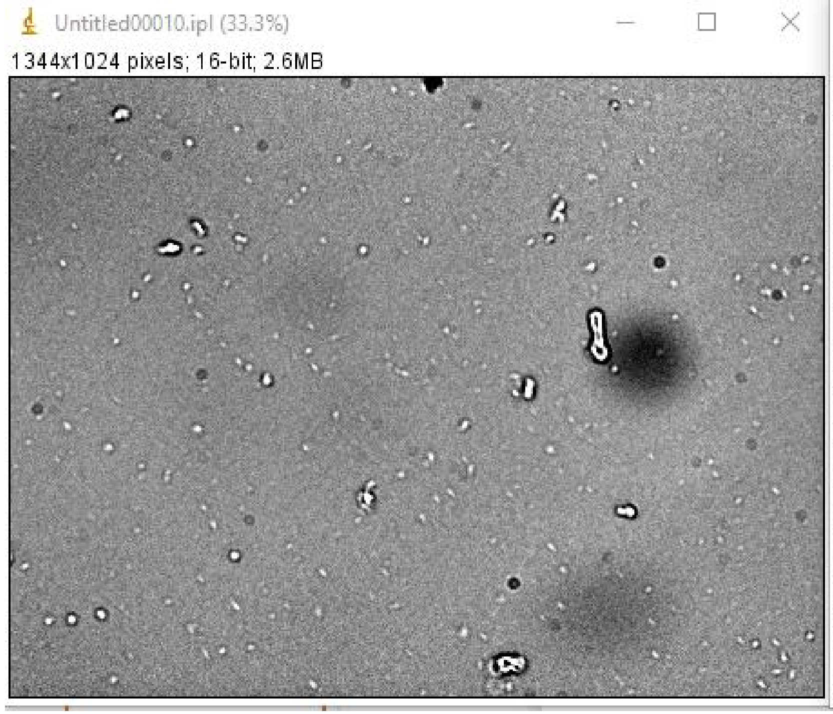

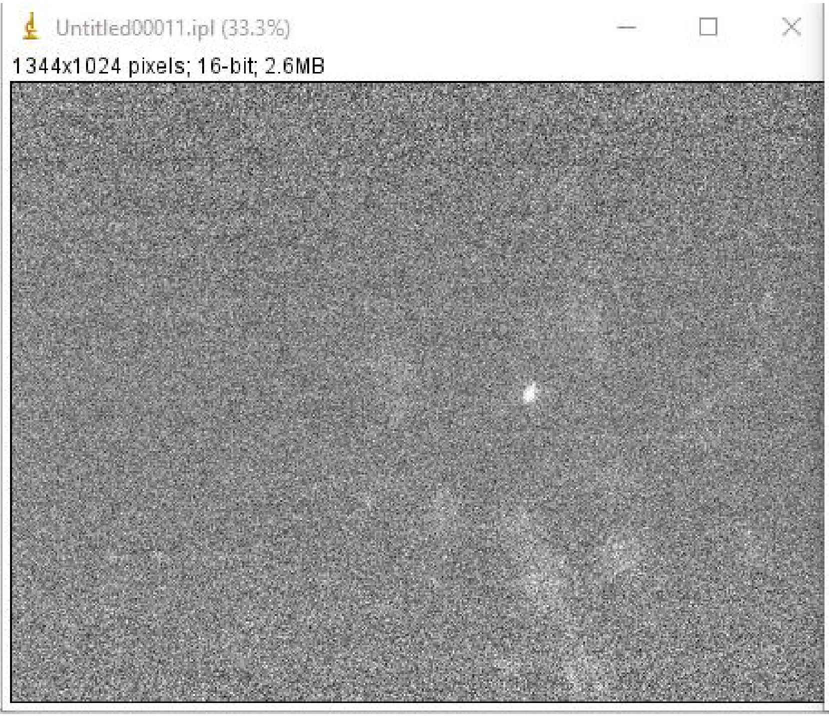

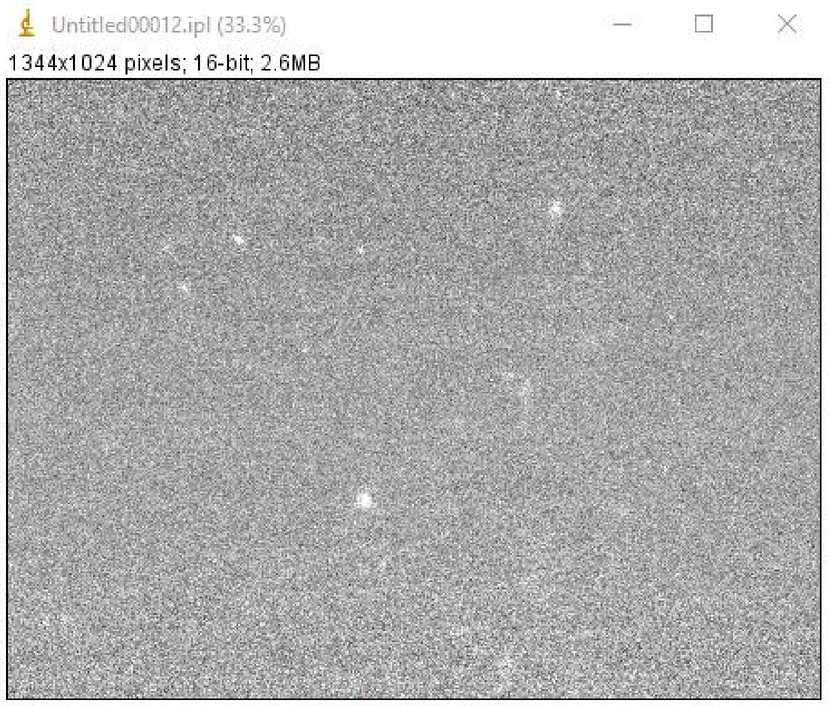

Whole supernantant - Sample 4

18 marks TxR

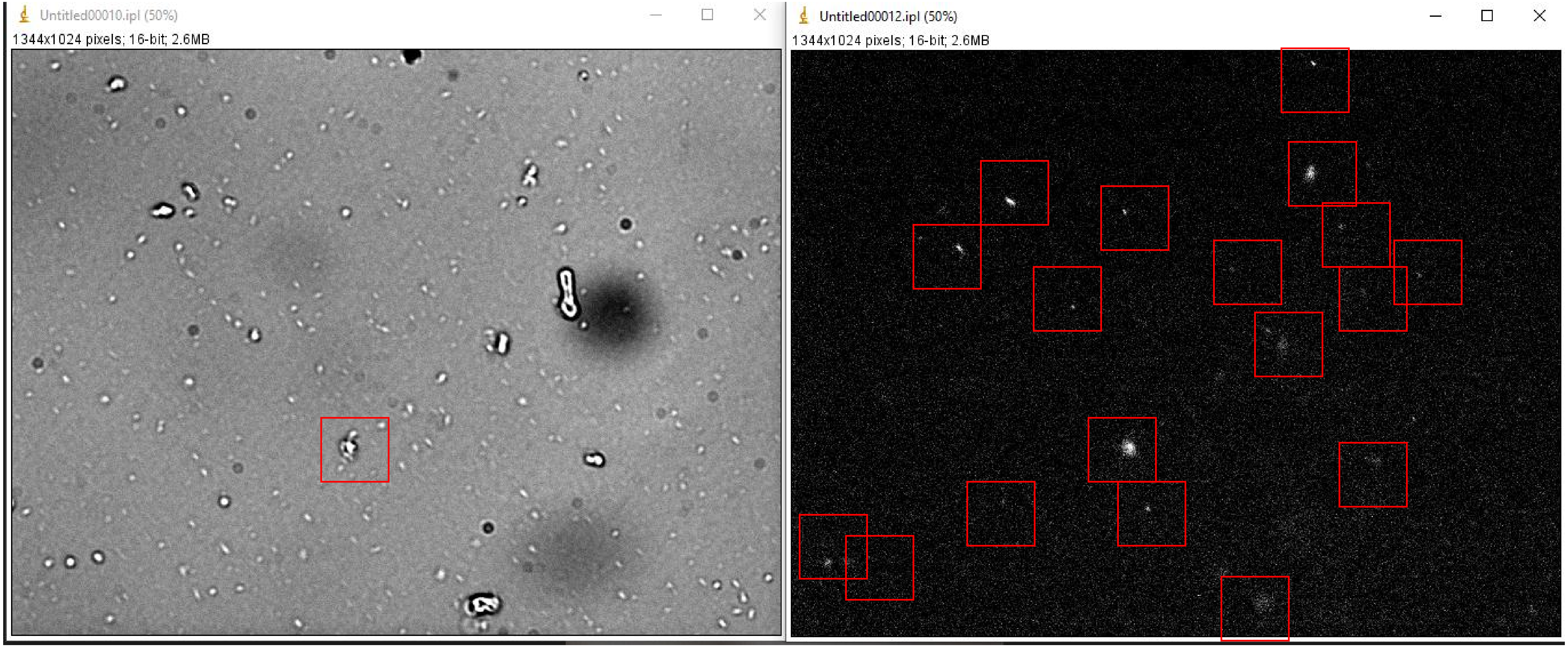

Whole supernantant - Sample 4

1 round mark

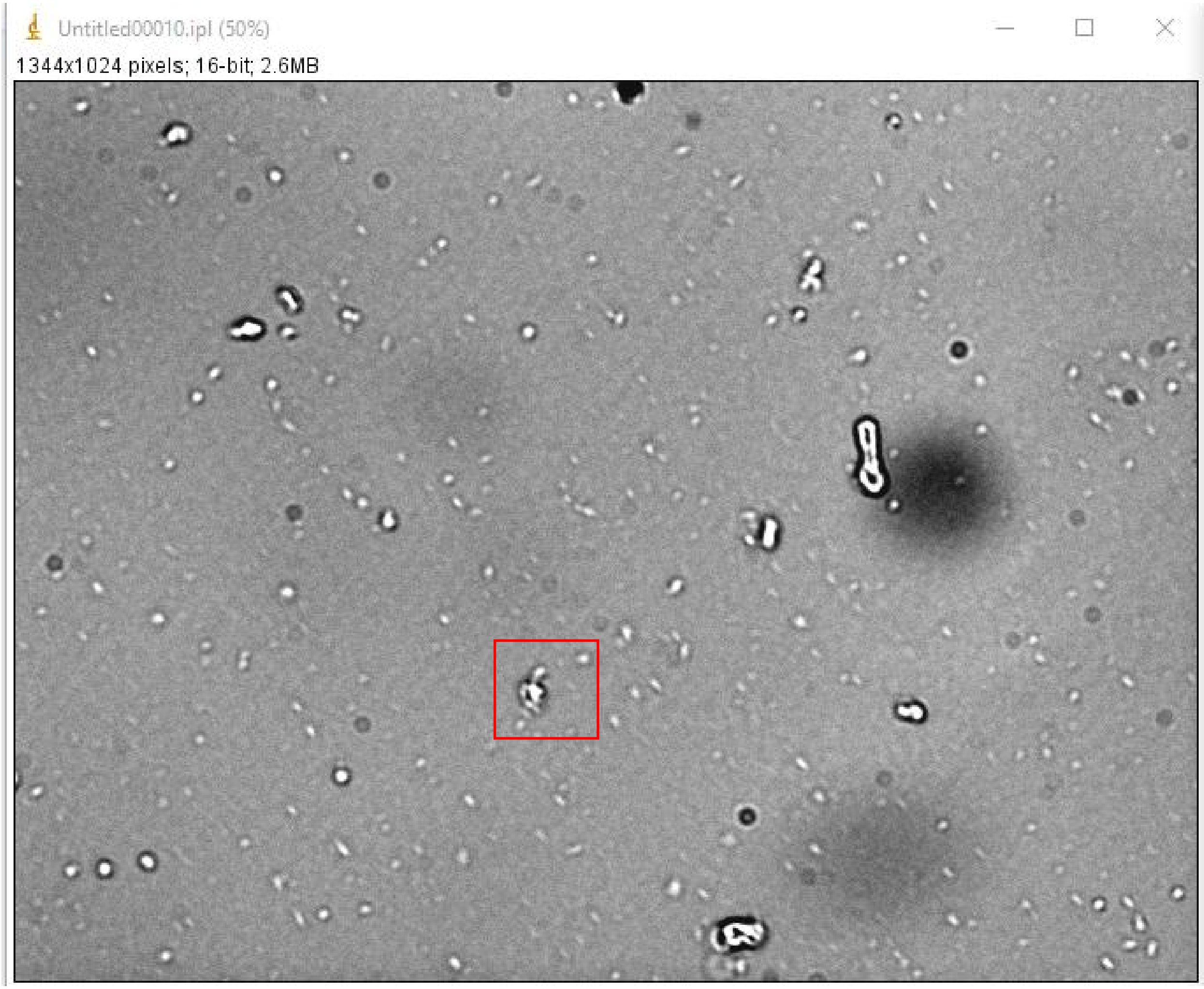

5 marks FITC

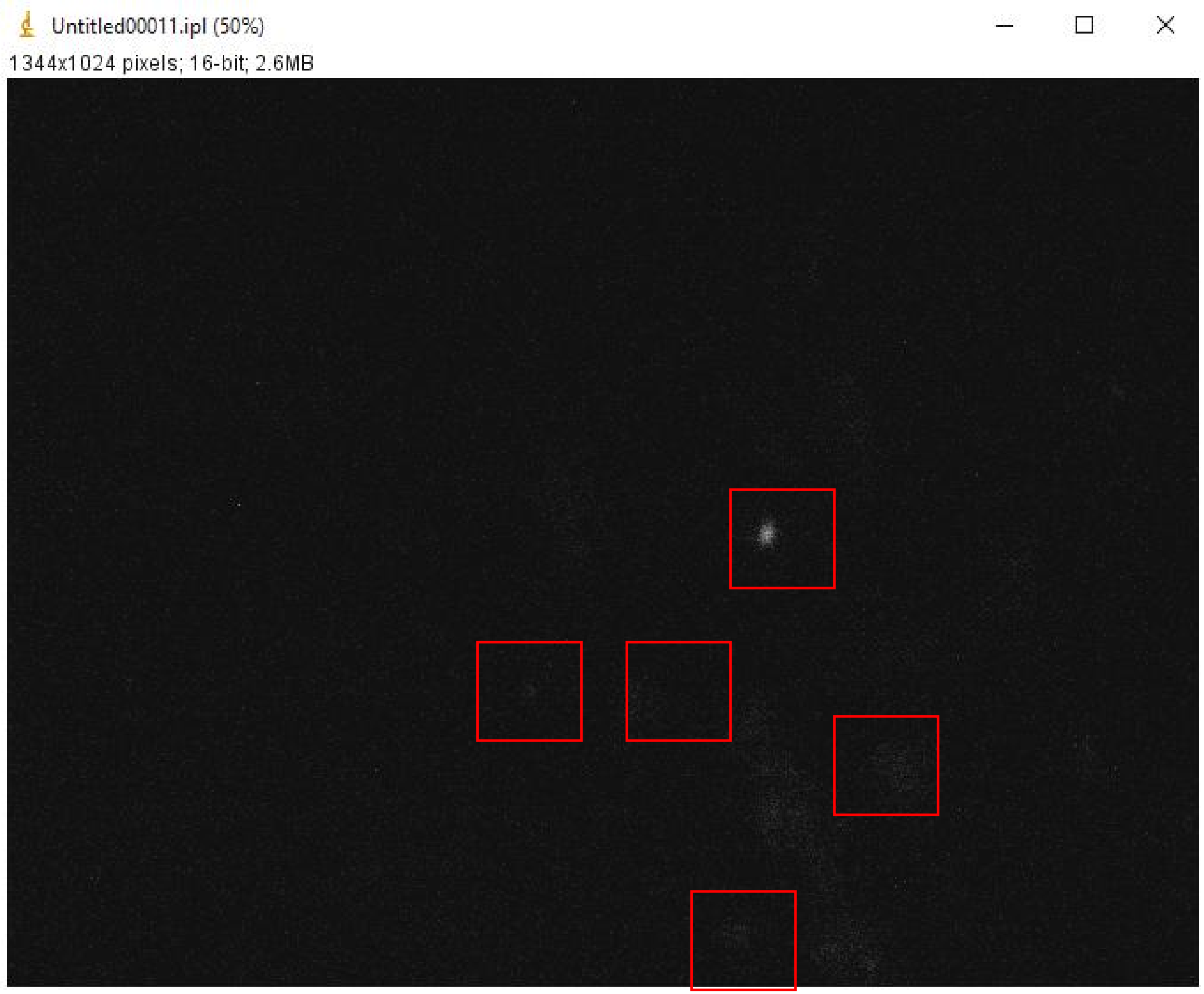

Whole supernantant - Sample 4

1 DIC + FITC + TxR merge

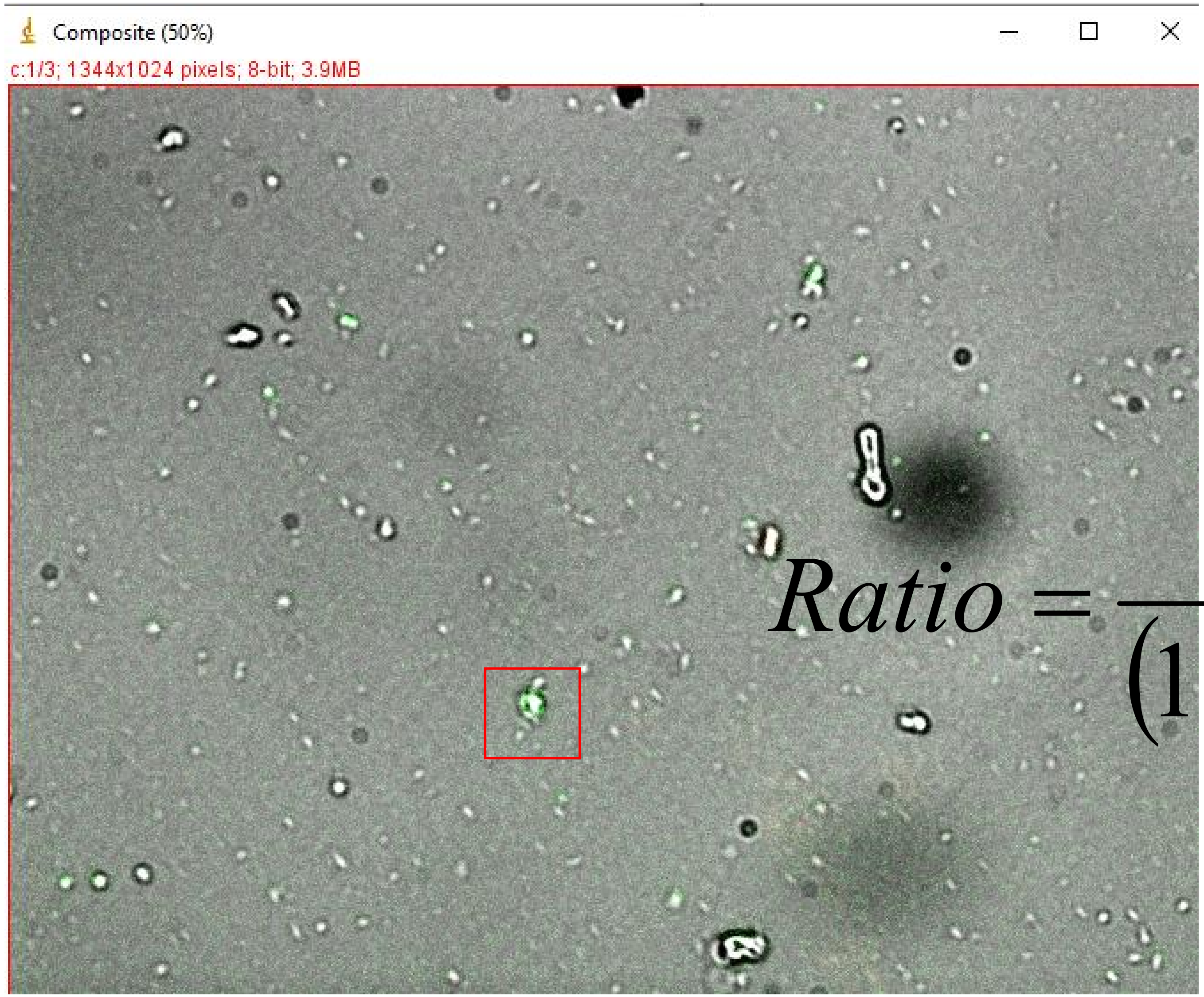

$$Ratio = \frac{1}{\left(18+5+1\right)/3} = 0.125$$

DS fraction - Sample 1

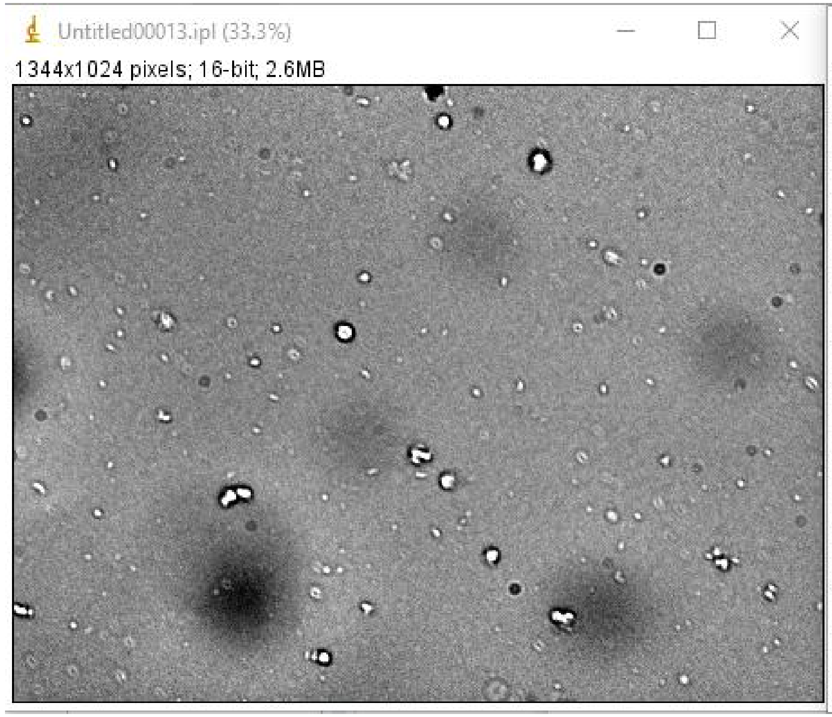

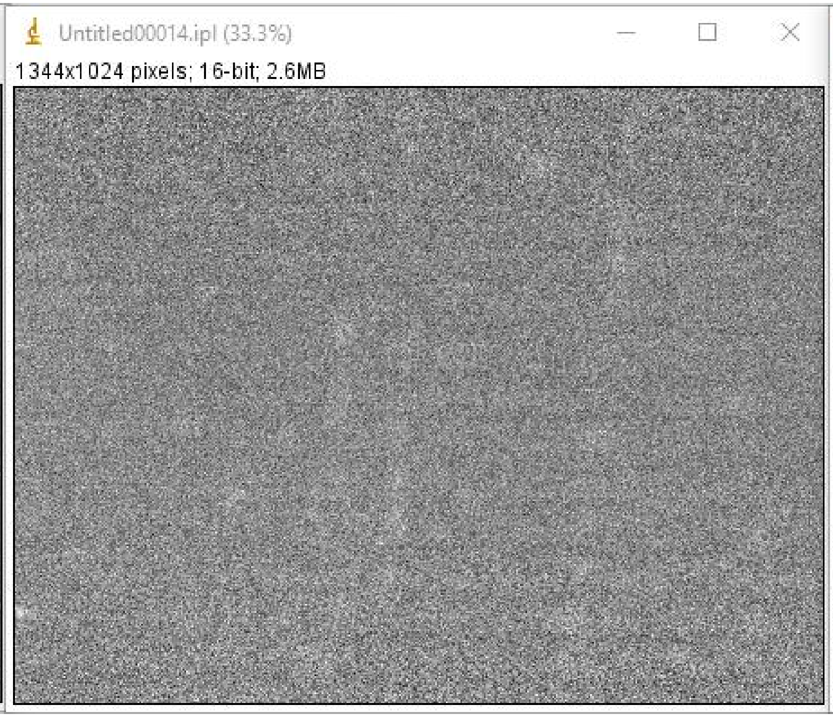

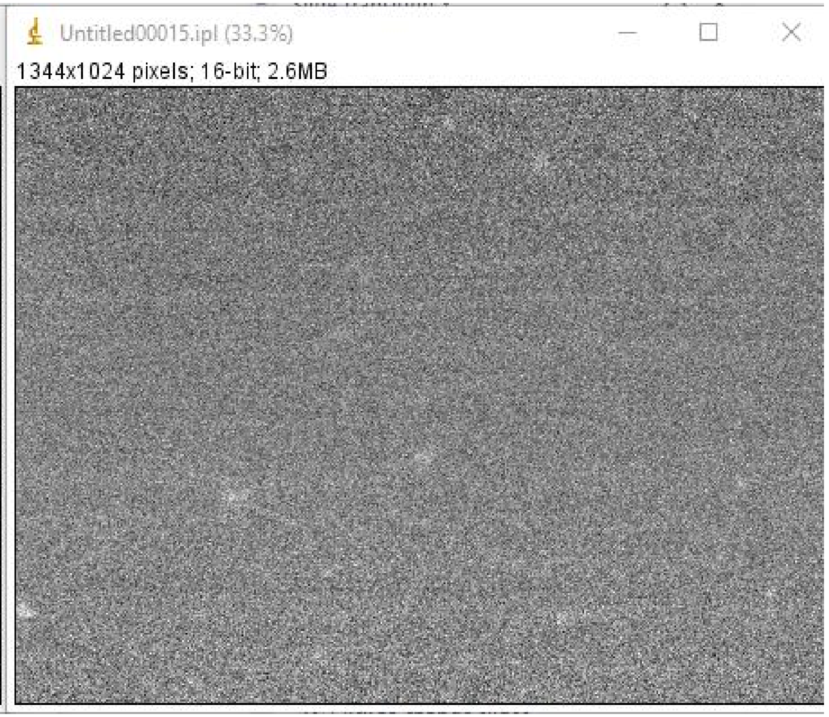

DS fraction - Sample 1

Round marks = 8

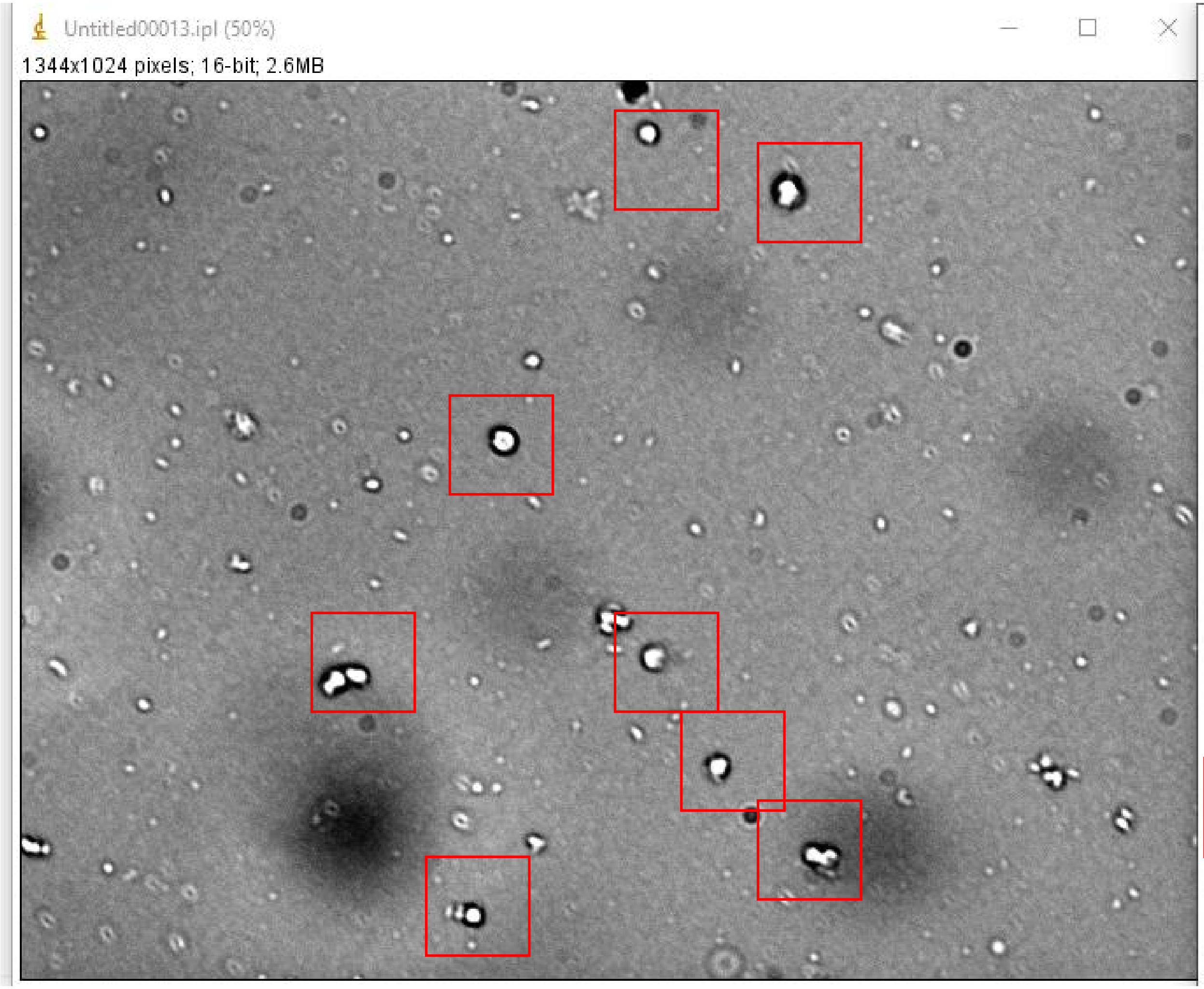

12 marks TxR

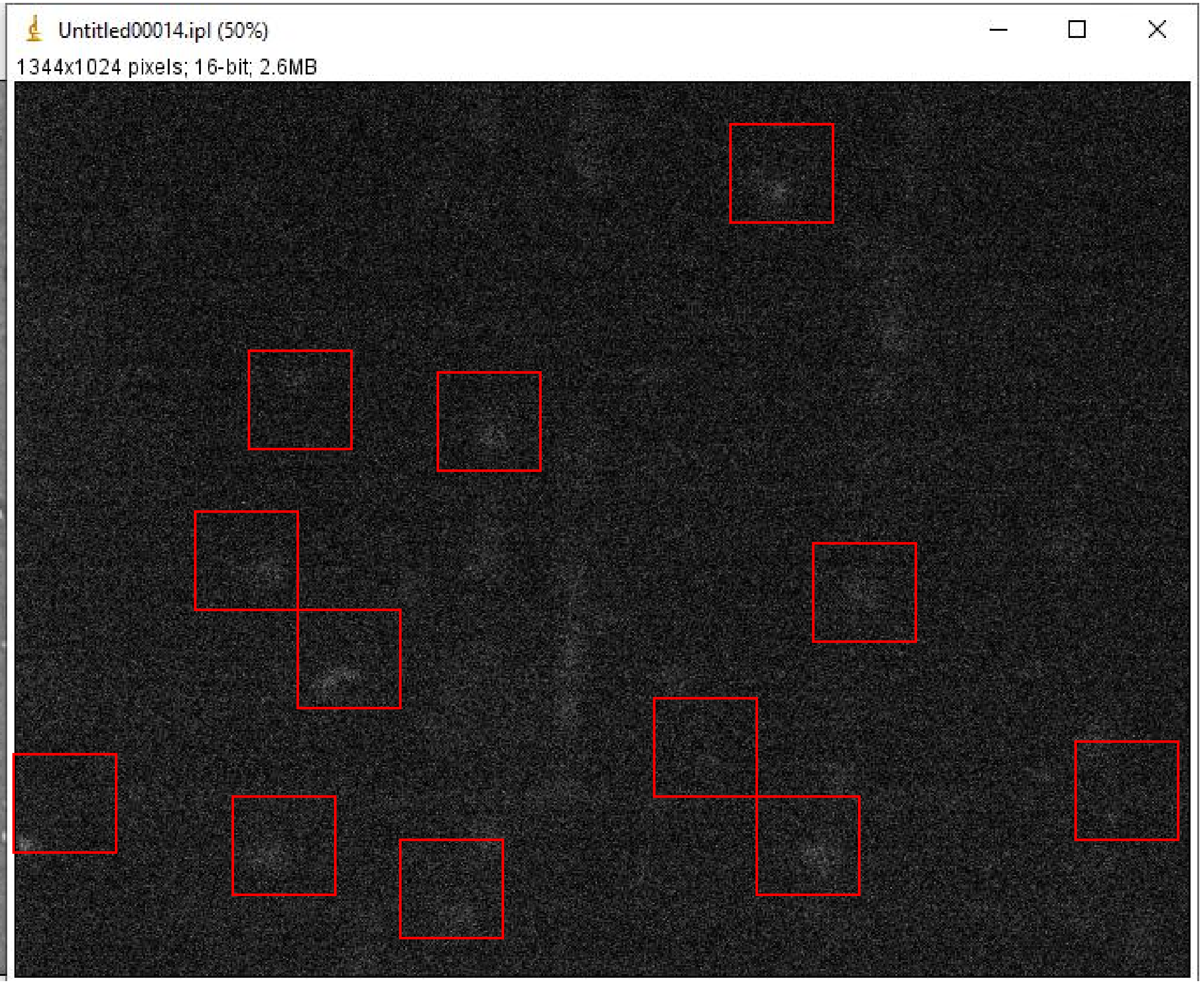

DS fraction - Sample 1

Round marks = 8

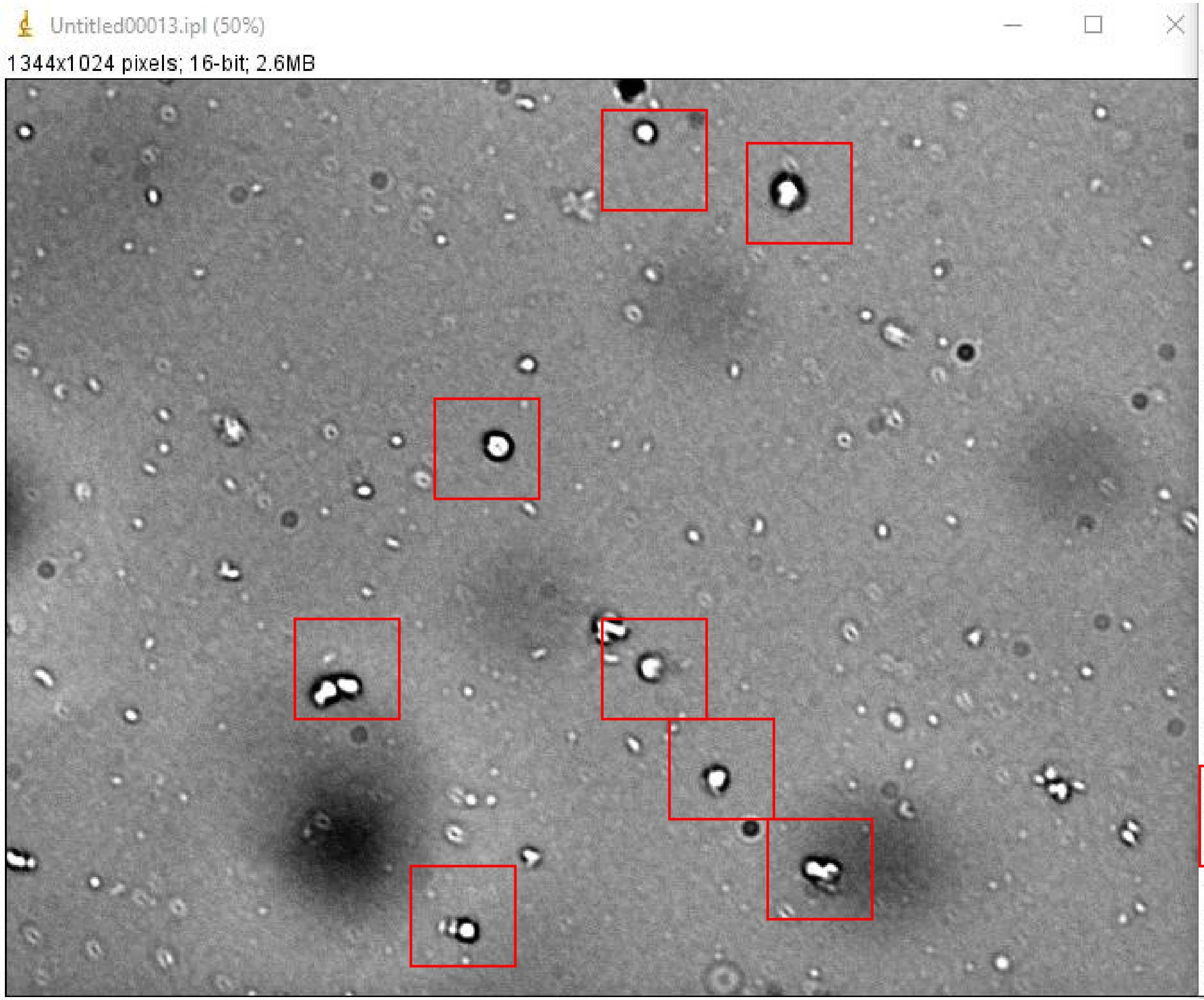

12 marks FITC

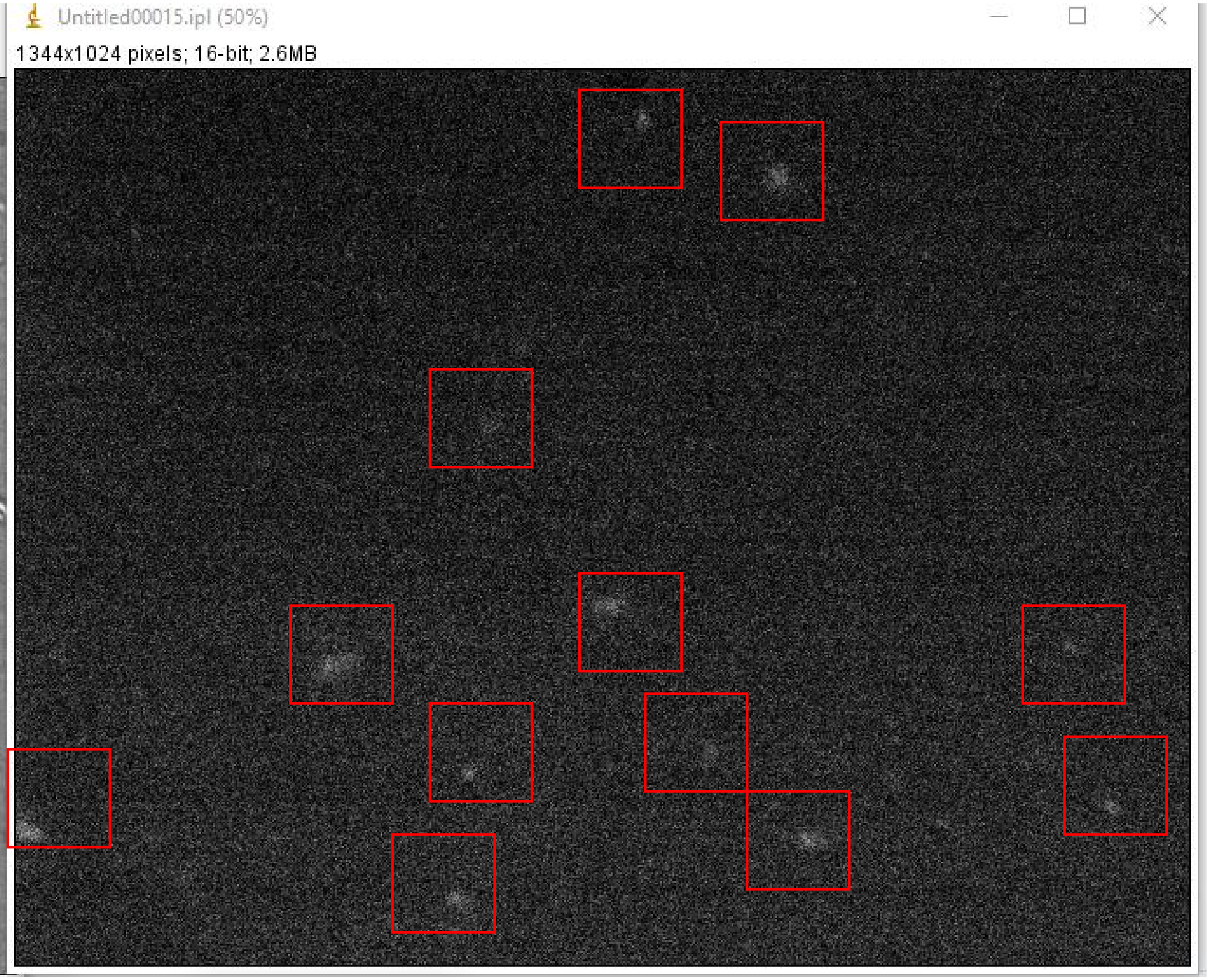

DS fraction - Sample 1

6 marks with the three criteria, merge

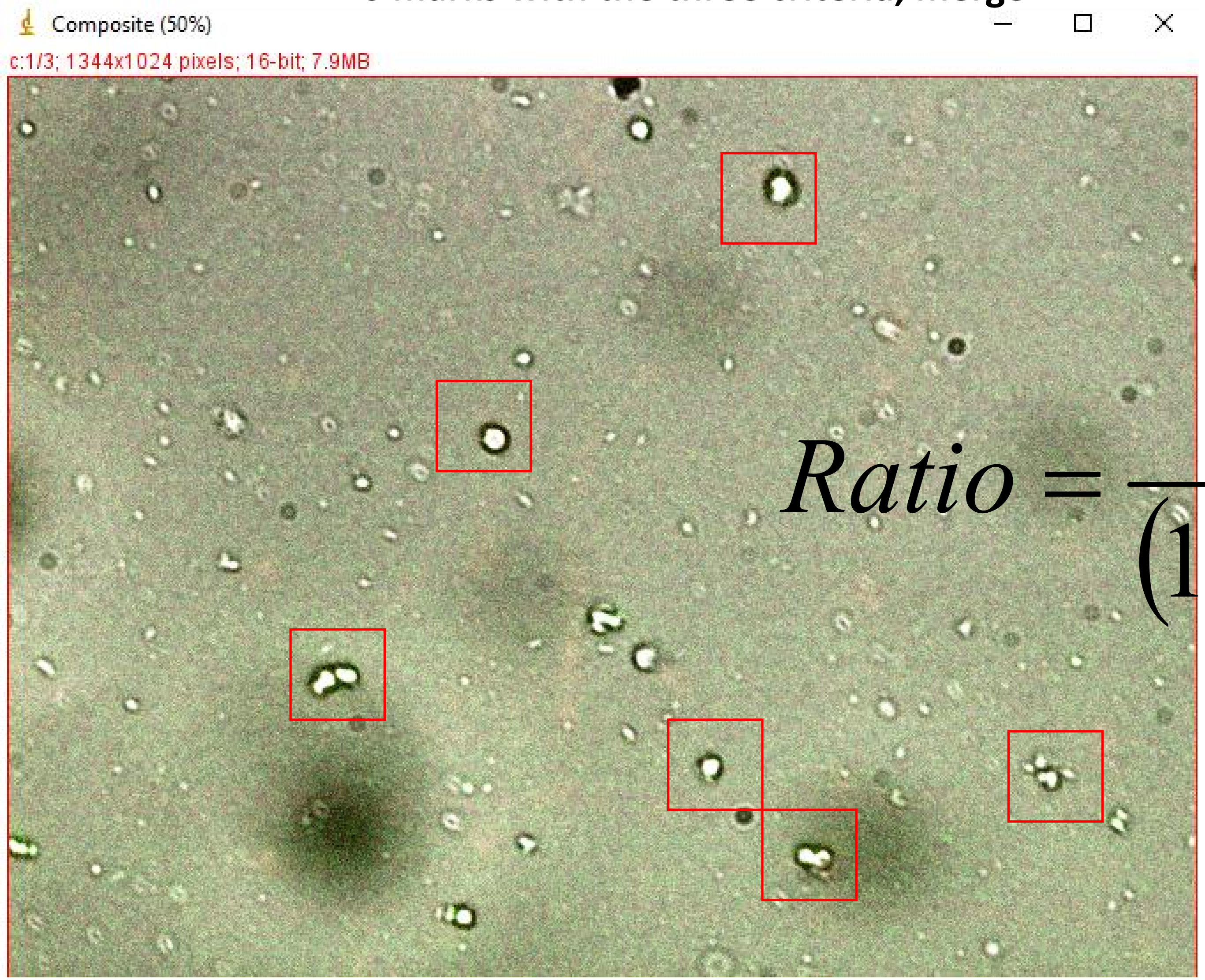

DS fraction - Sample 1

$$Ratio = \frac{6}{(12+12+8)/3} = 0.563$$

DS fraction - Sample 2

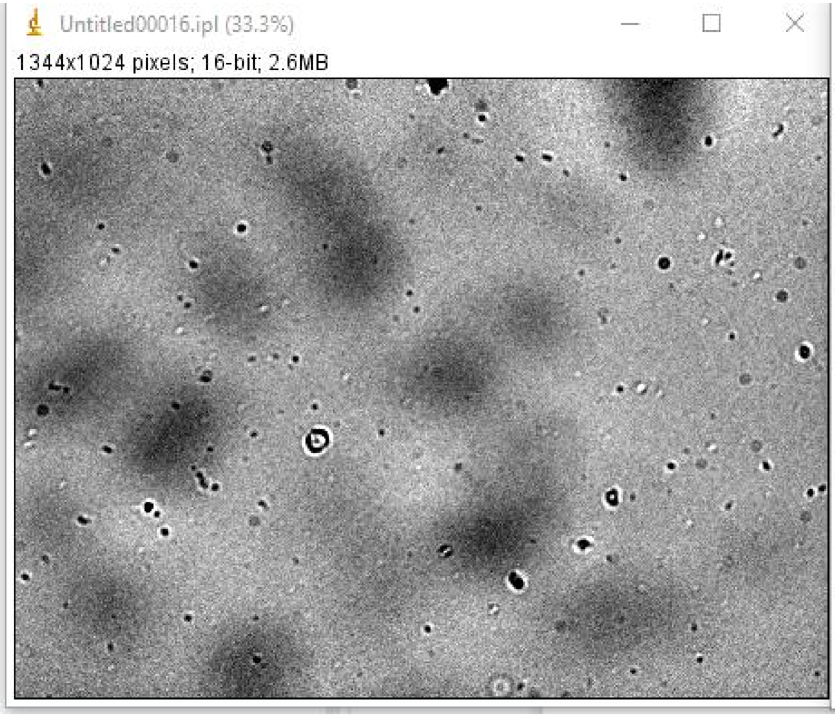

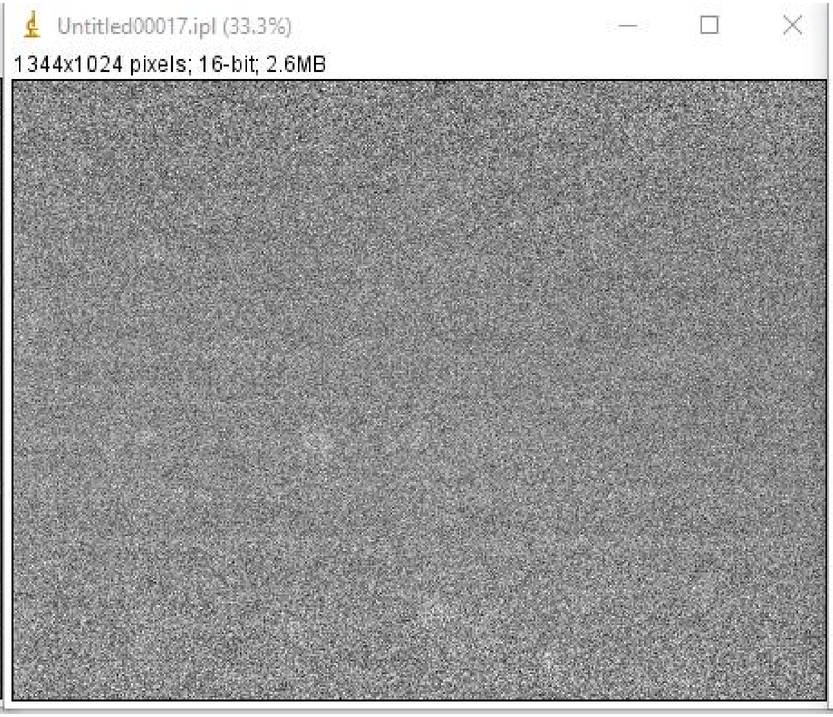

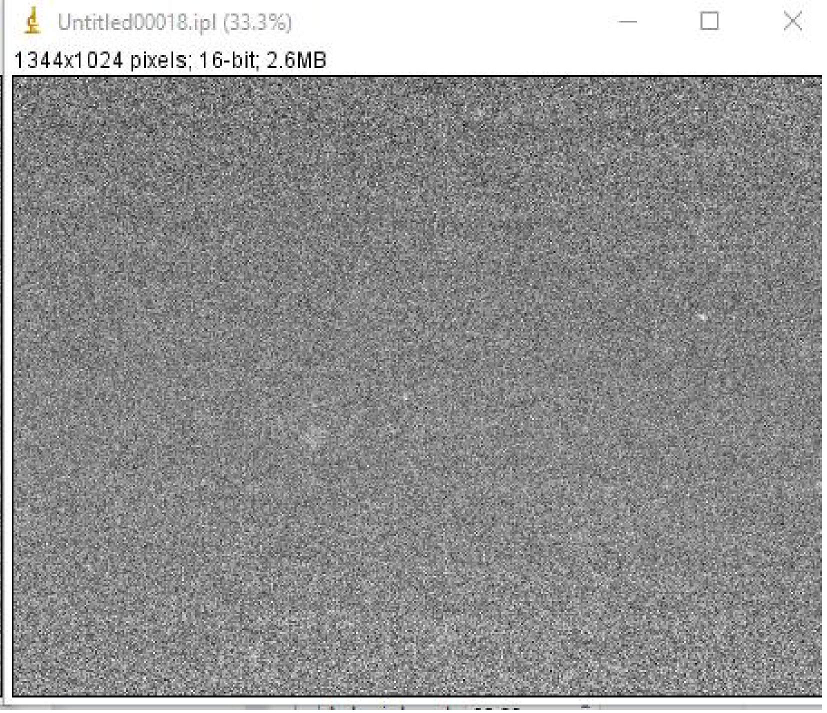

DS fraction - Sample 2

Ronud marks = 1

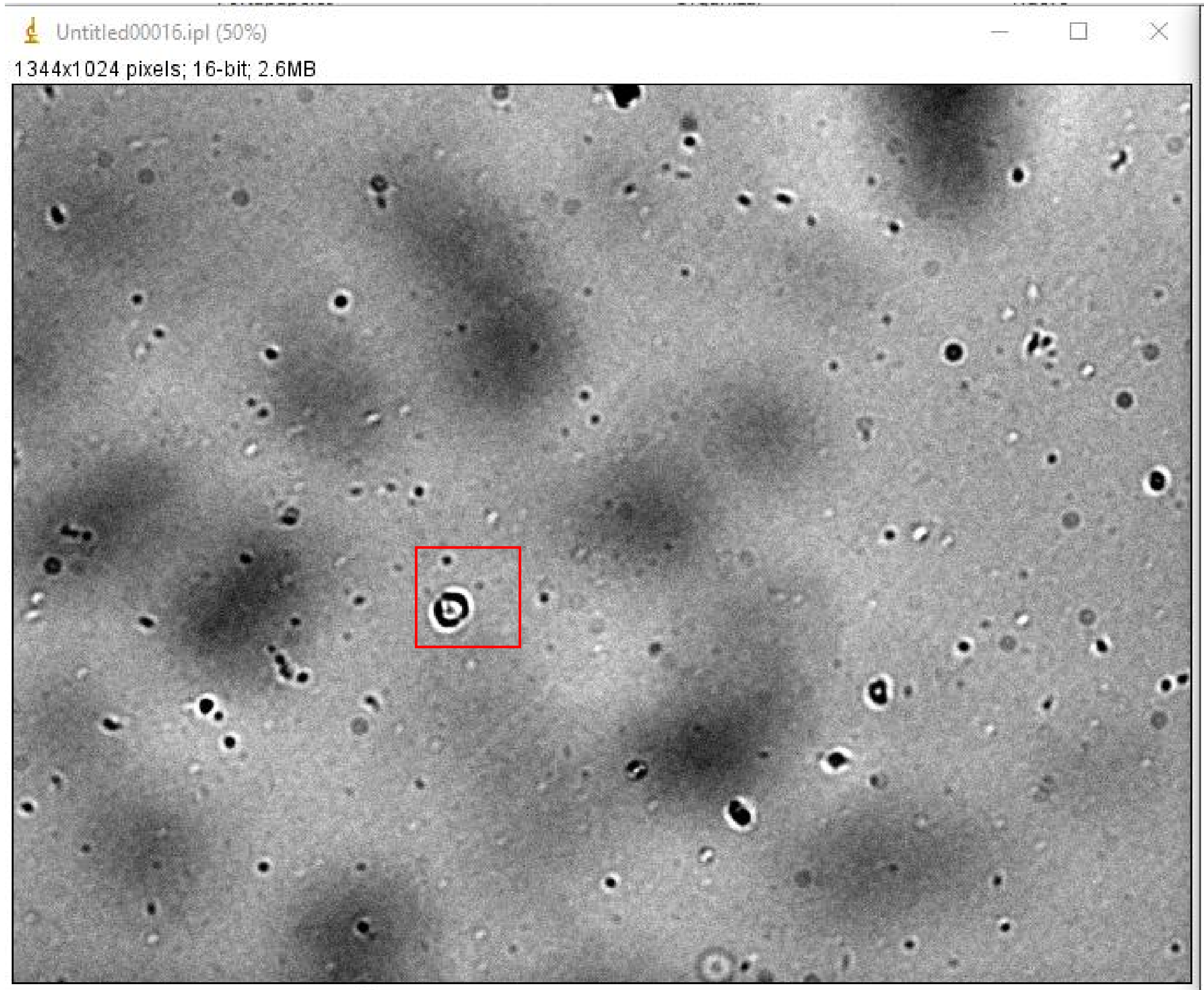

3 marks TxR

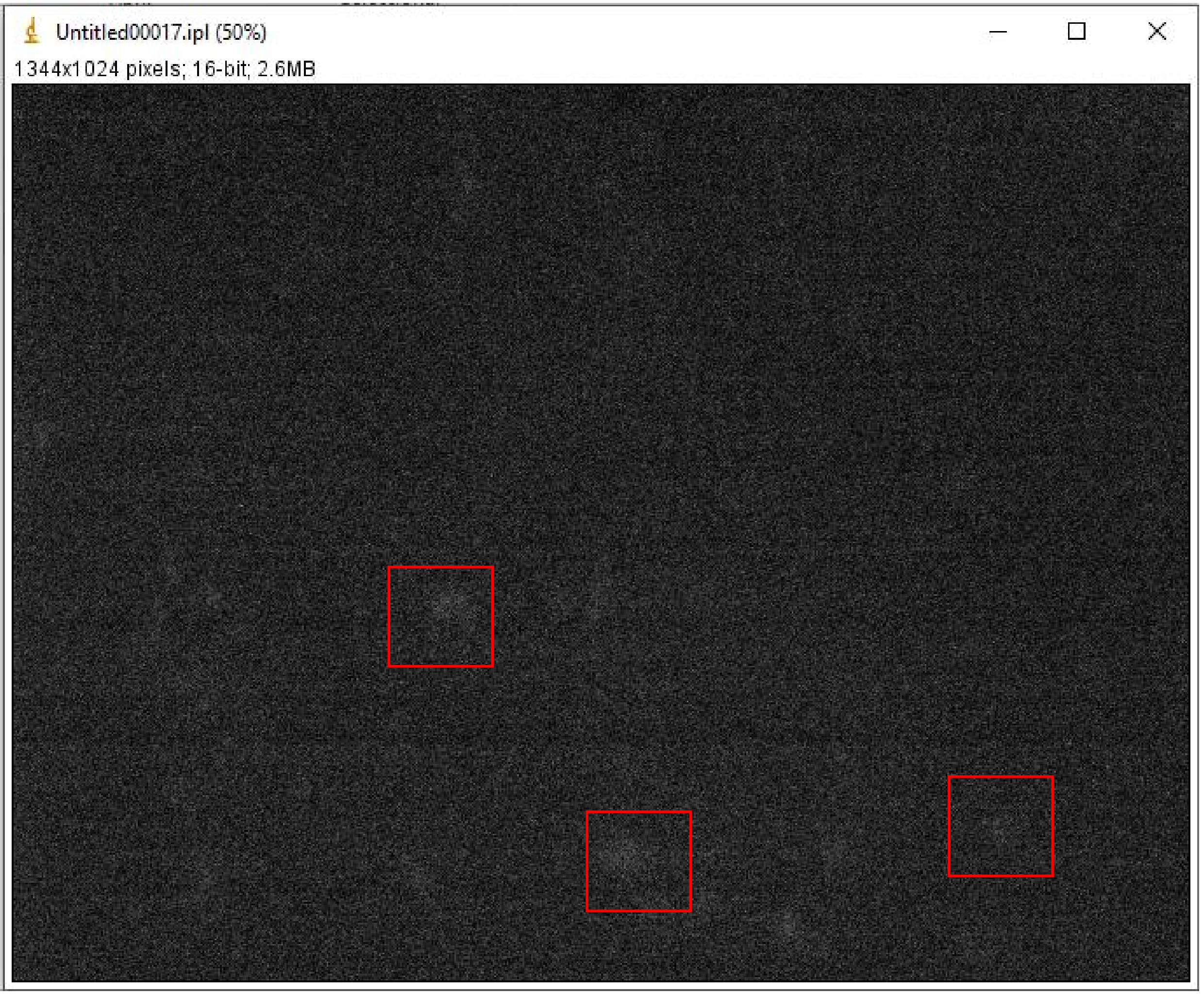

DS fraction - Sample 2

Round marks = 1

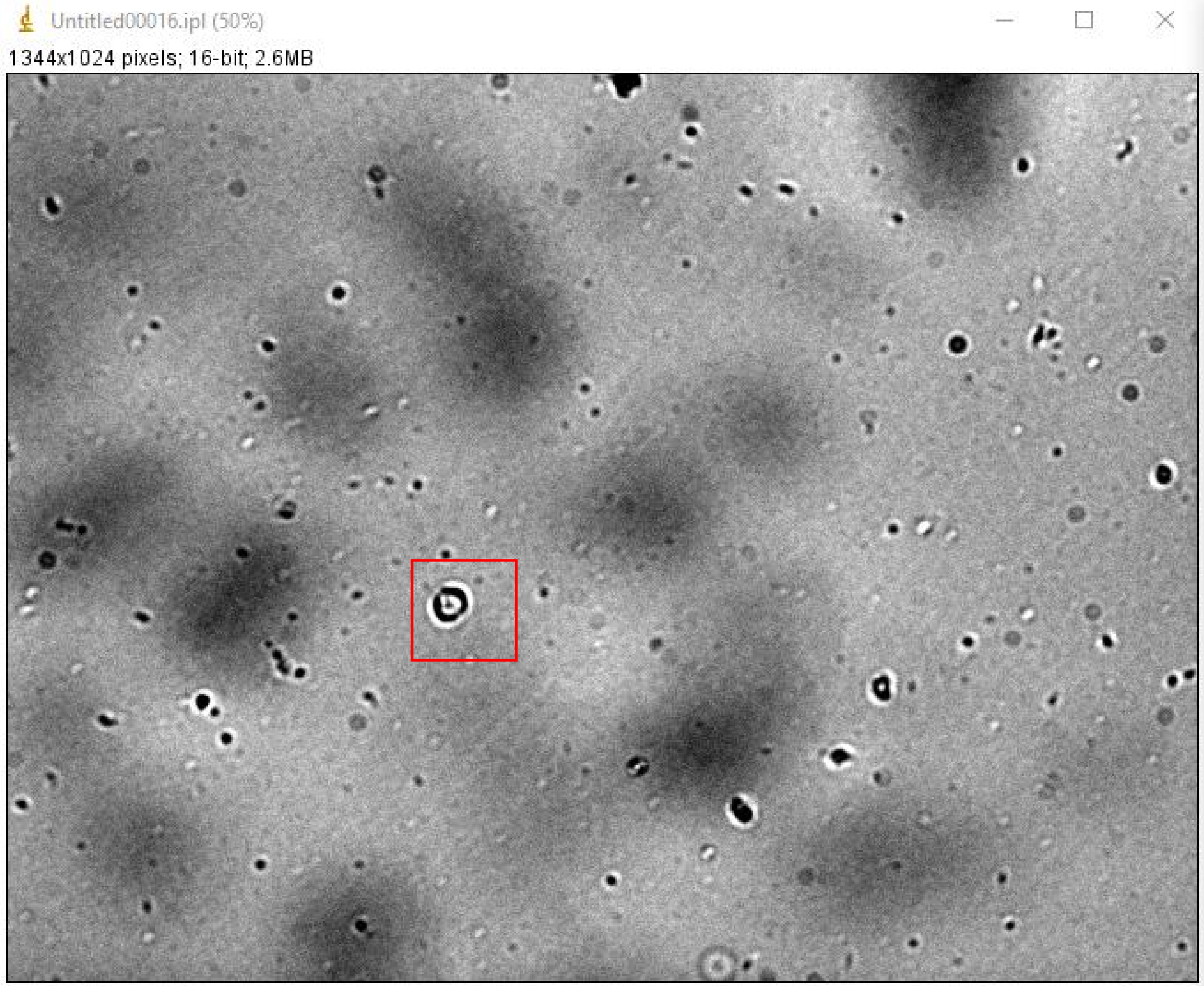

4 marks FITC

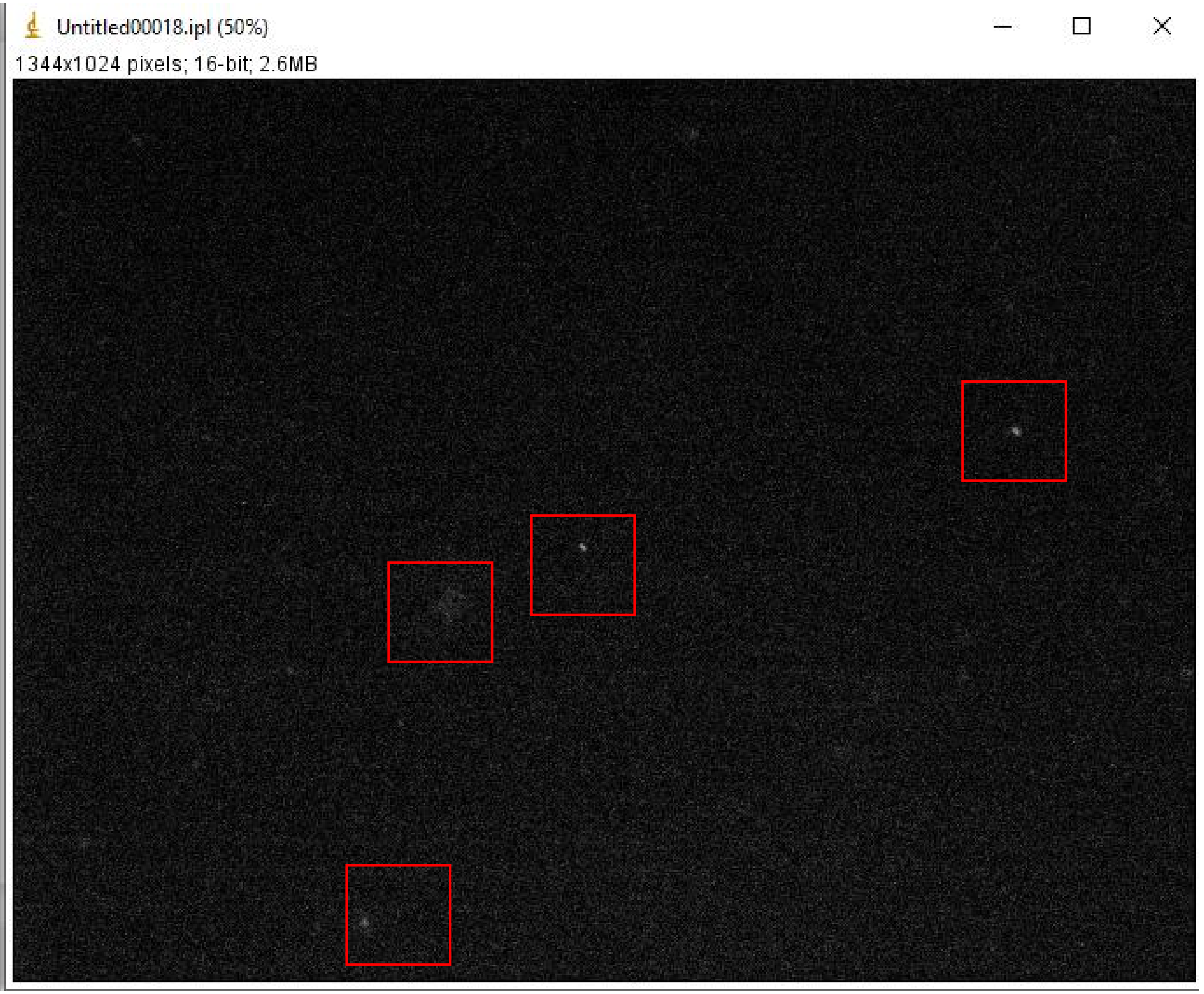

DS fraction - Sample 2

DS fraction - Sample 2

One mark with the three criteria, merge

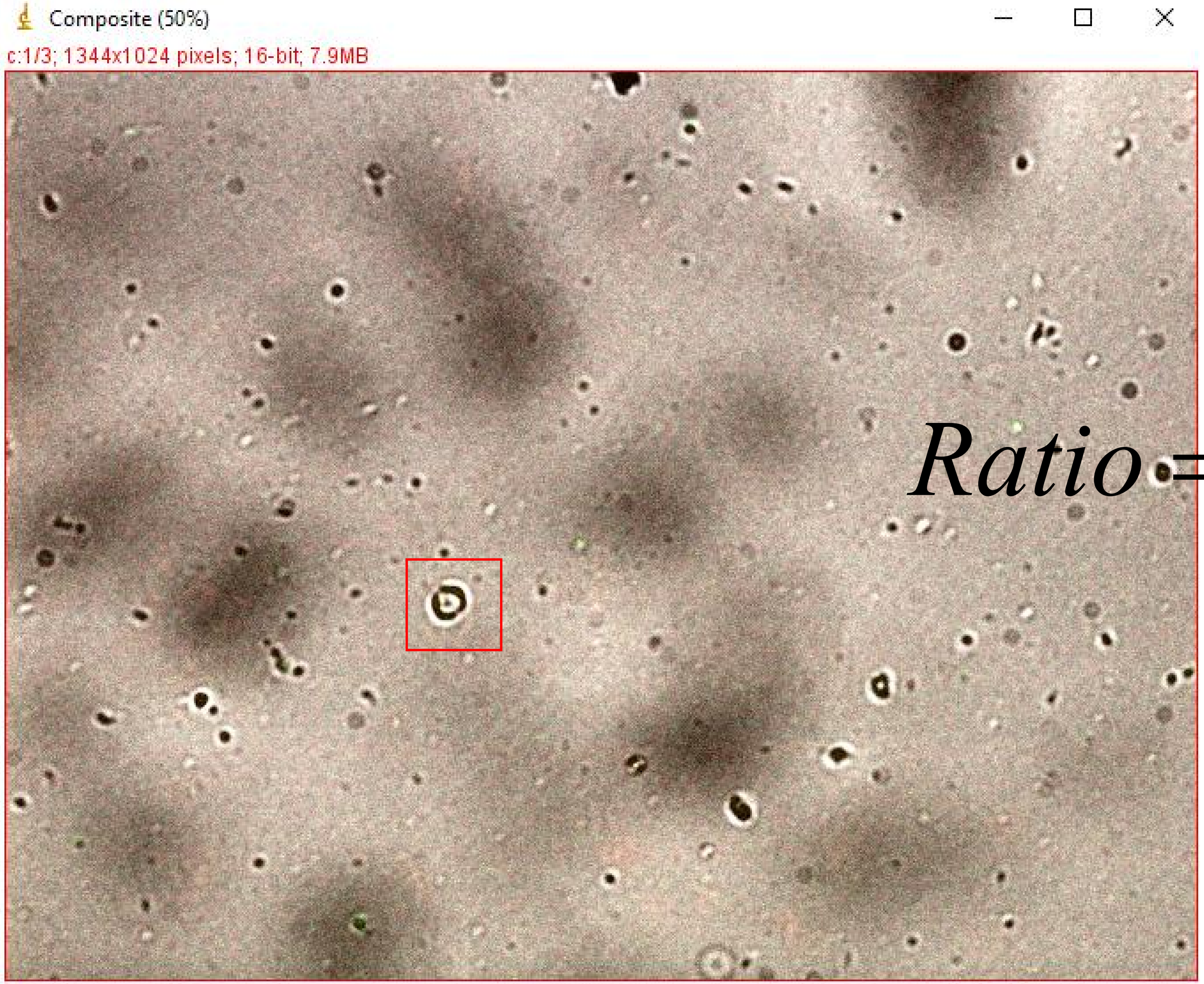

$$Ratio = \frac{1}{(1+3+4)/3} = 0.375$$

DS fraction - Sample 3

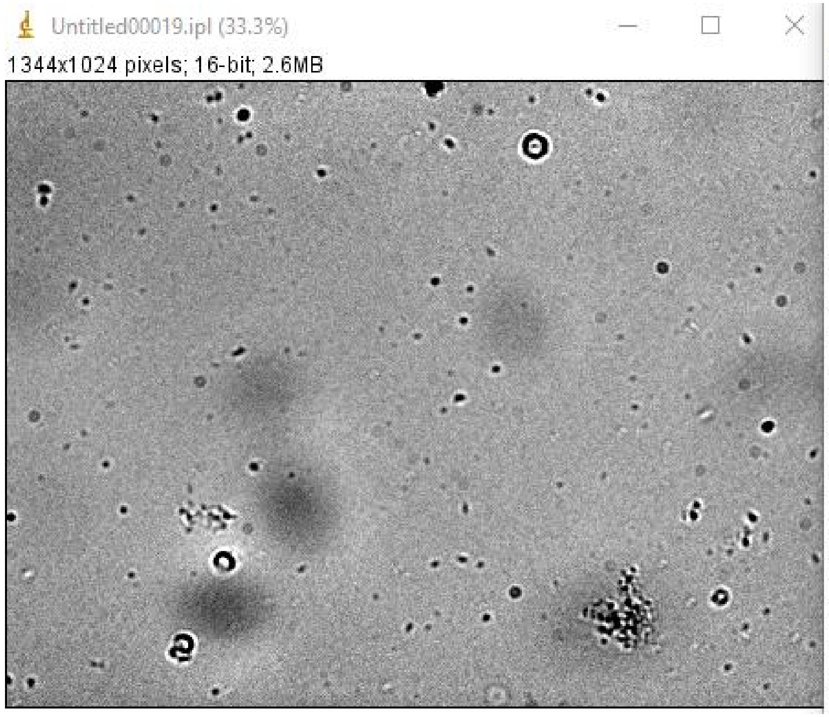

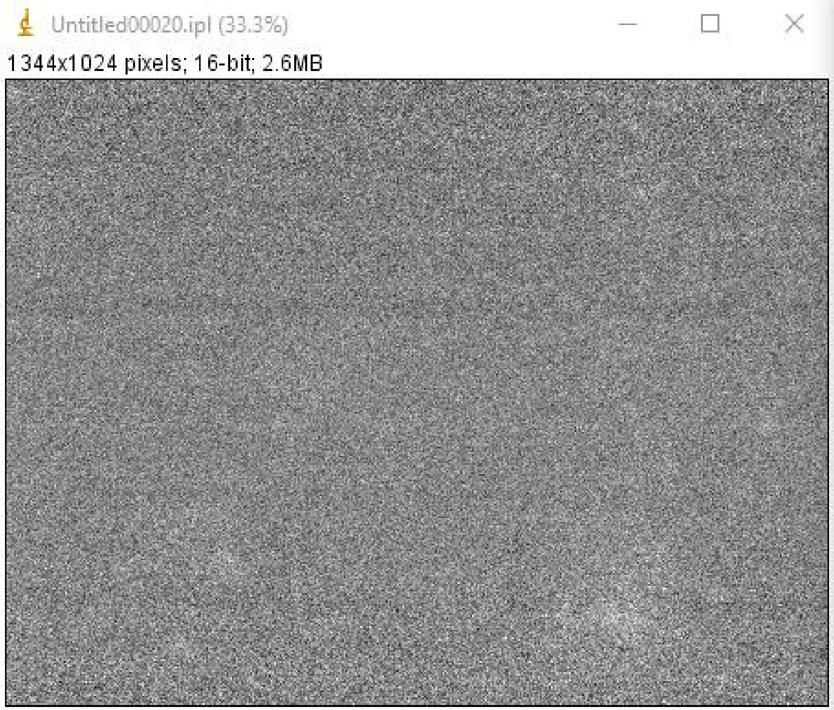

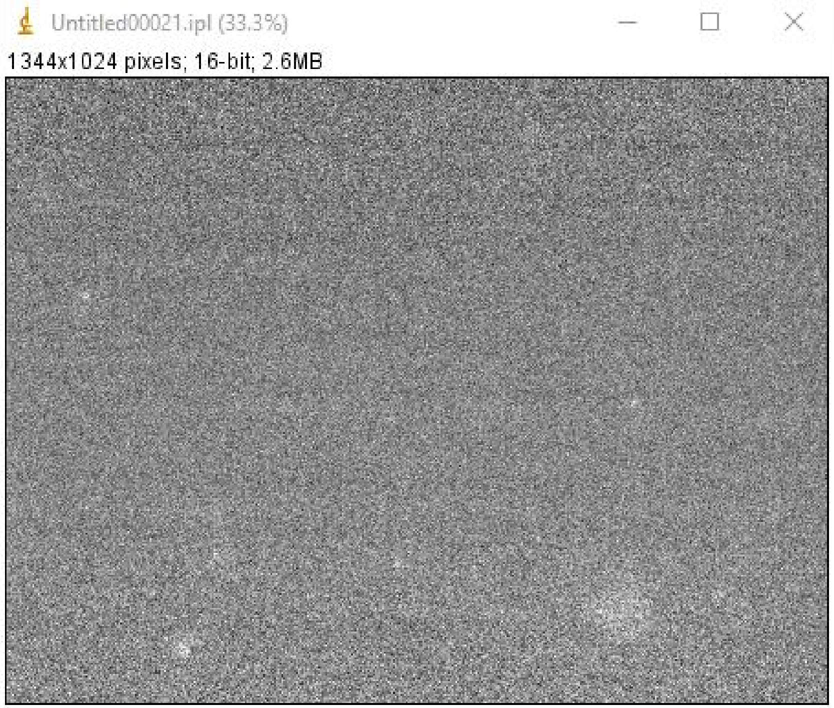

DS fraction - Sample 3

Round marks = 4

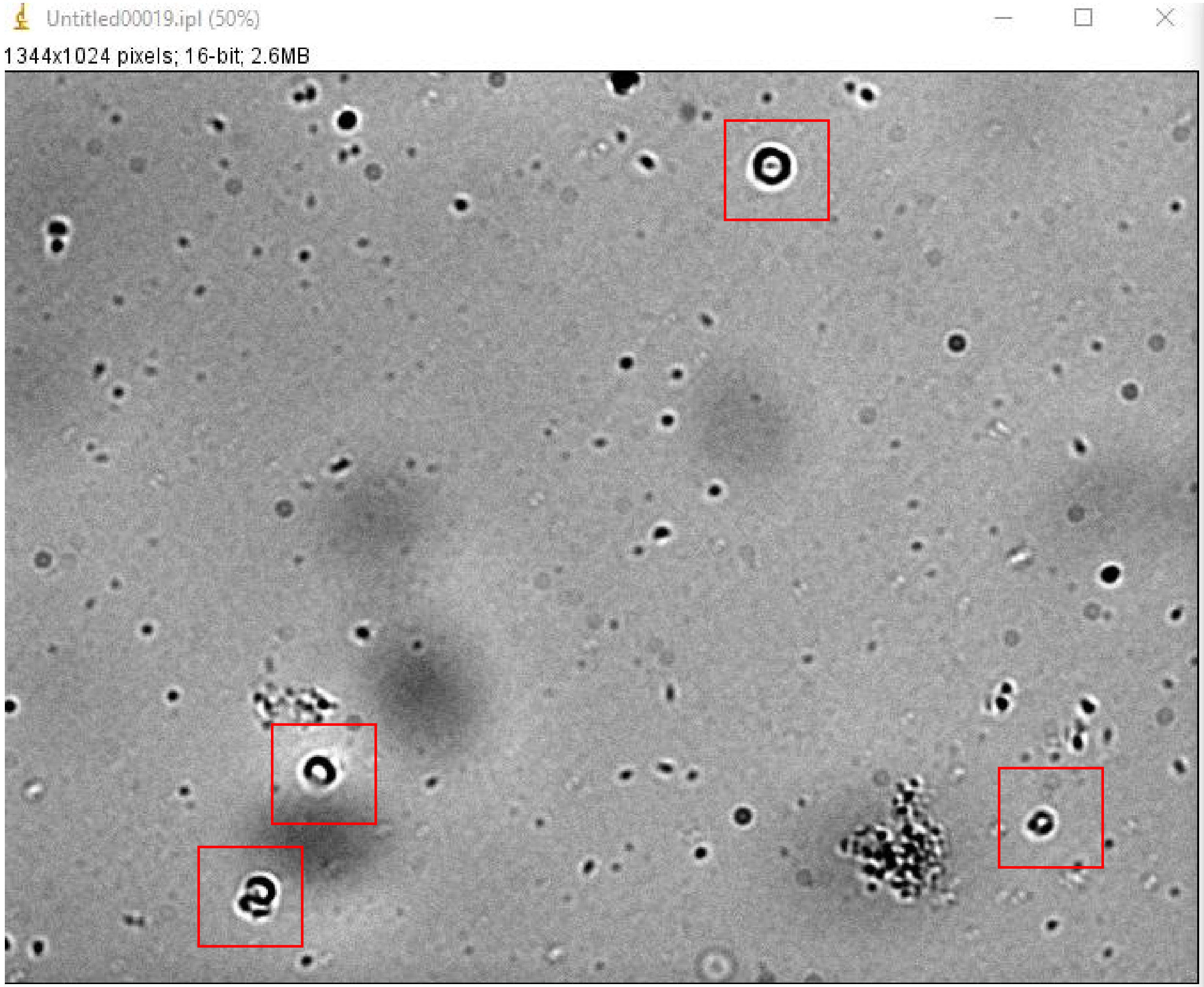

5 marks TxR

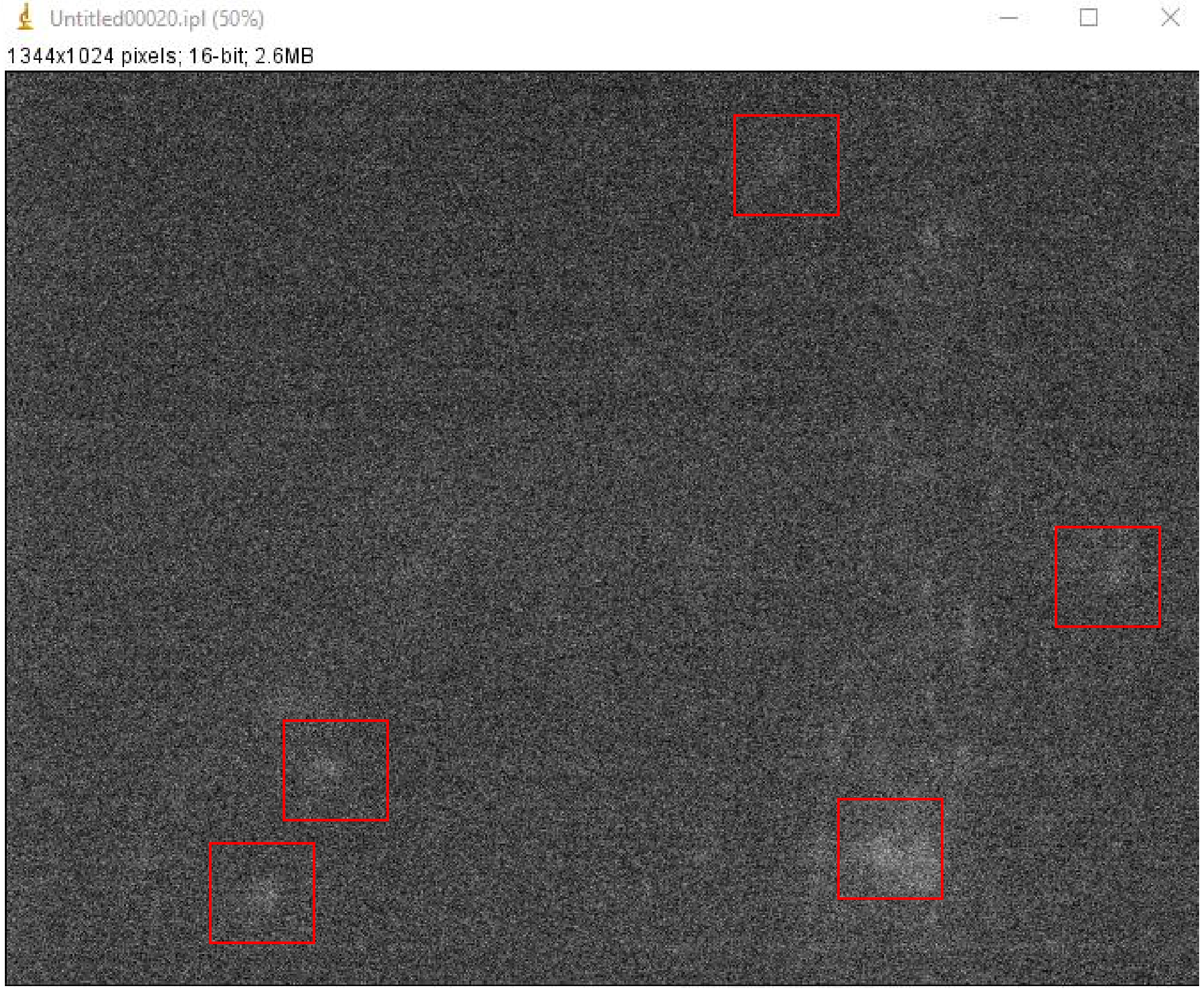

DS fraction - Sample 3

Round marks = 4

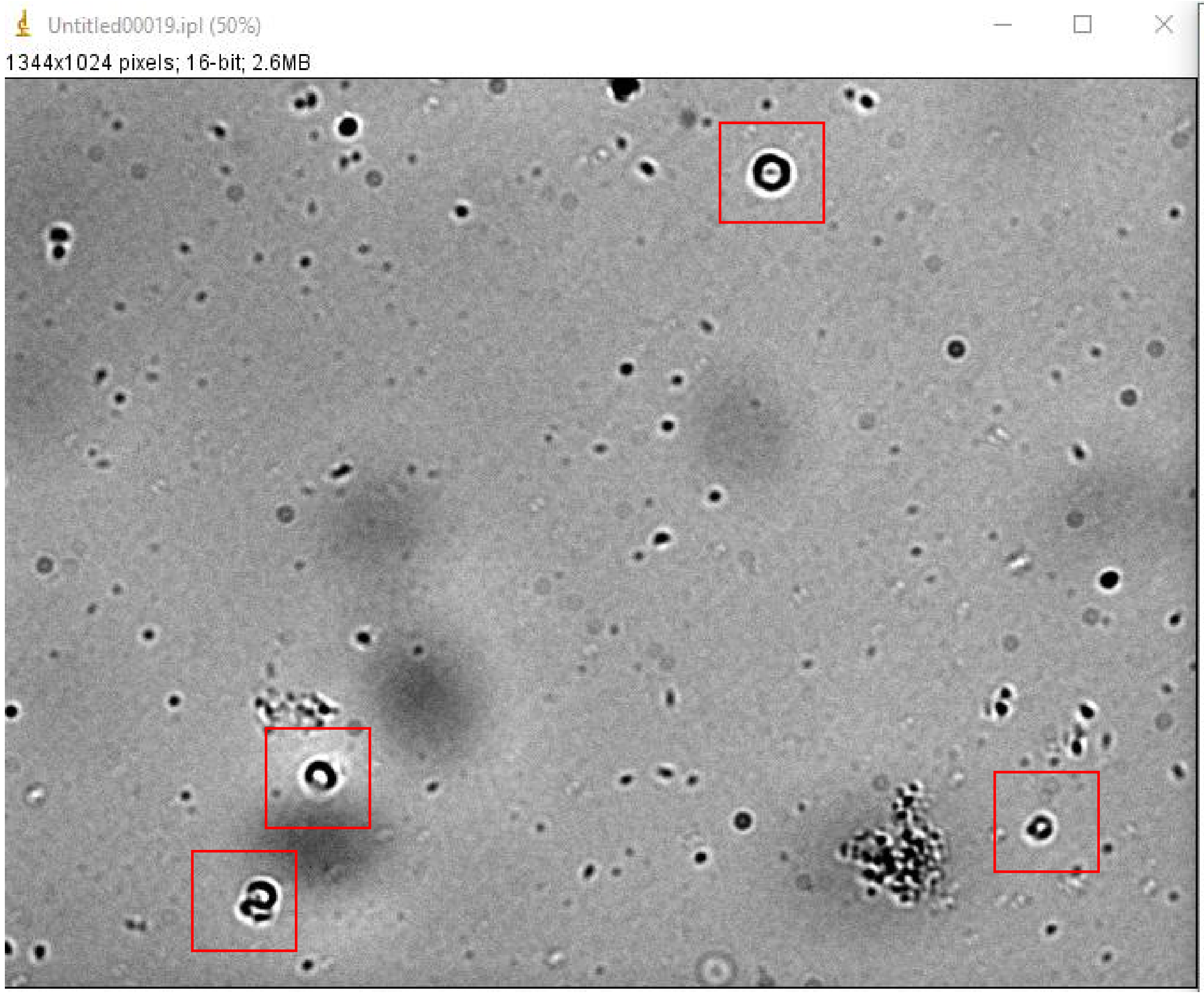

11 marks FITC

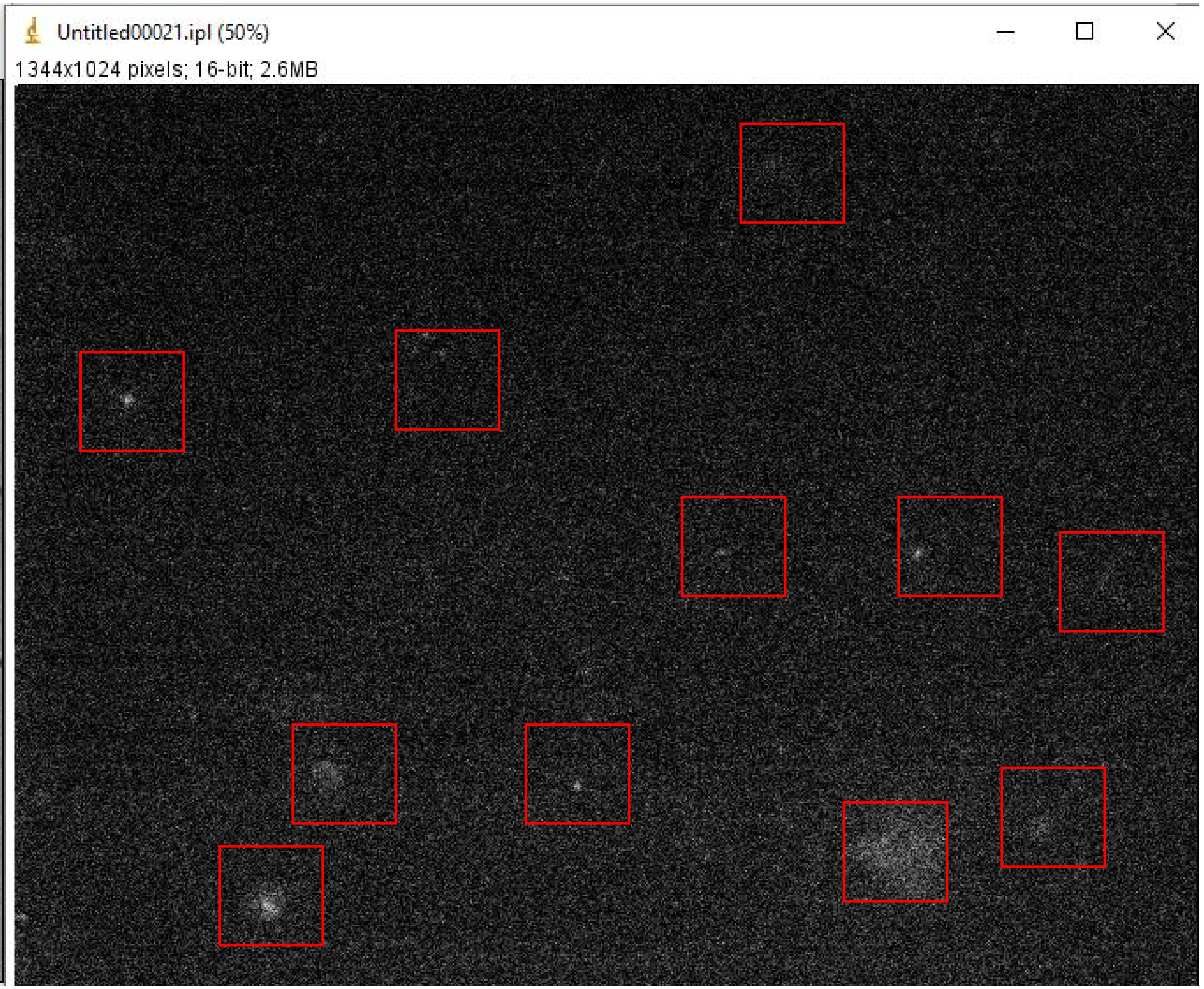

DS fraction - Sample 3

4 marks with the three criteria, merge

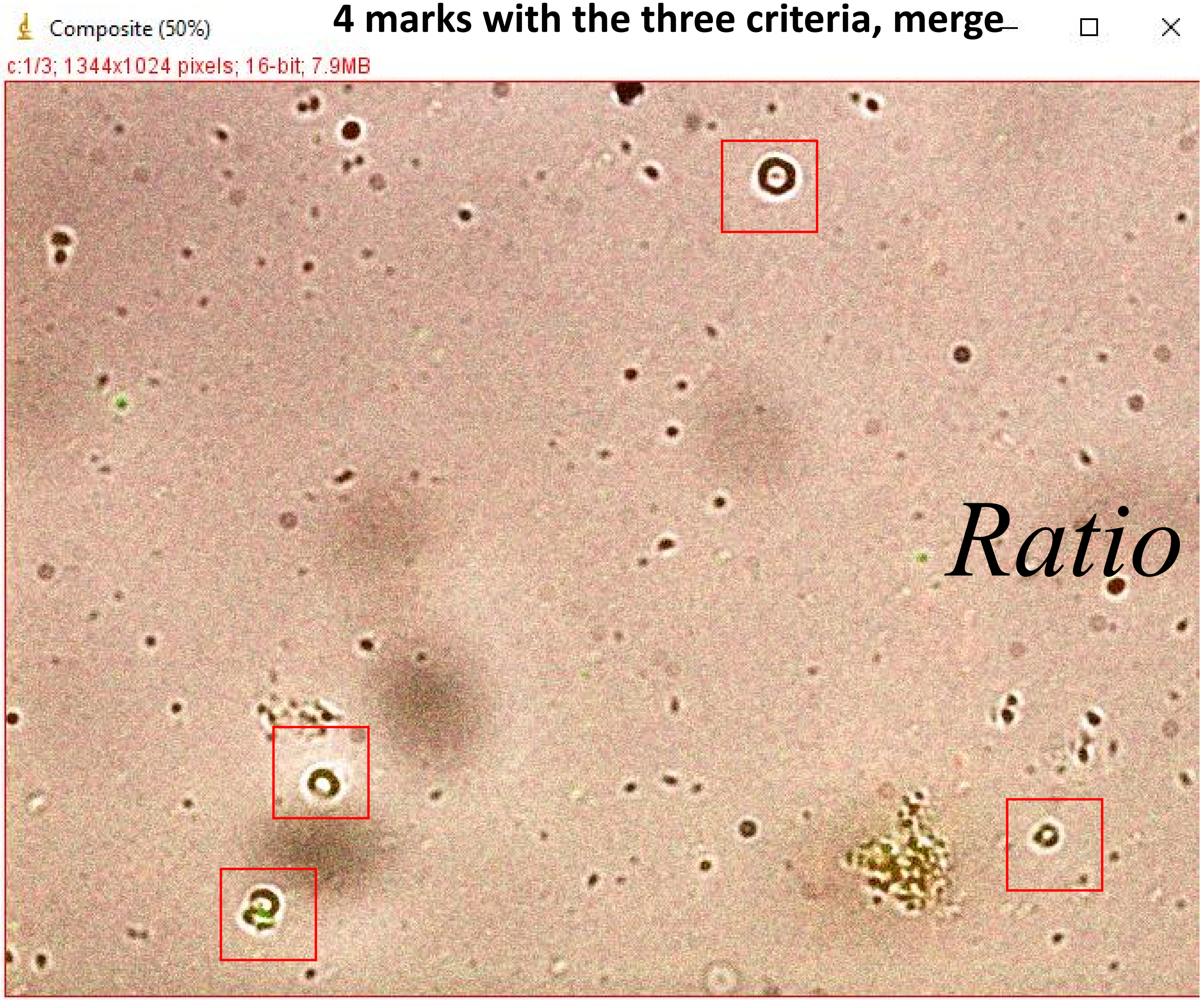

$$Ratio = \frac{4}{(4+11+5)/3} = 0.6$$

DS fraction - Sample 4

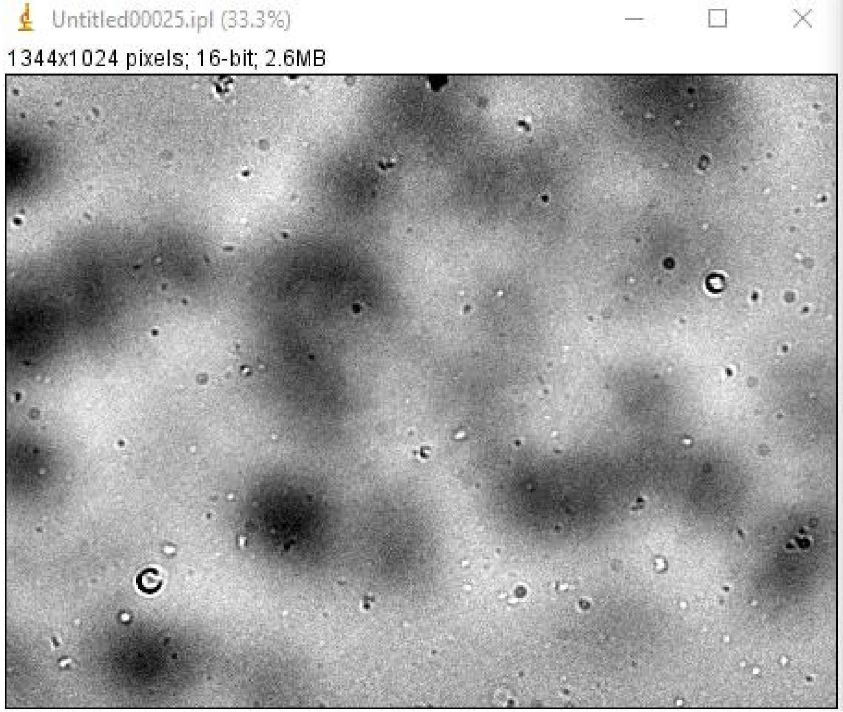

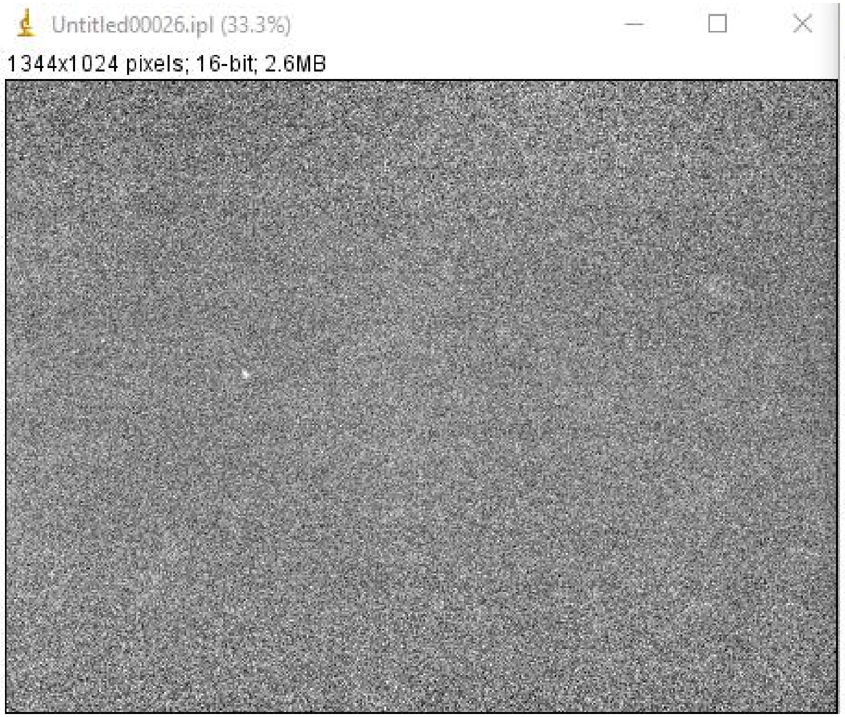

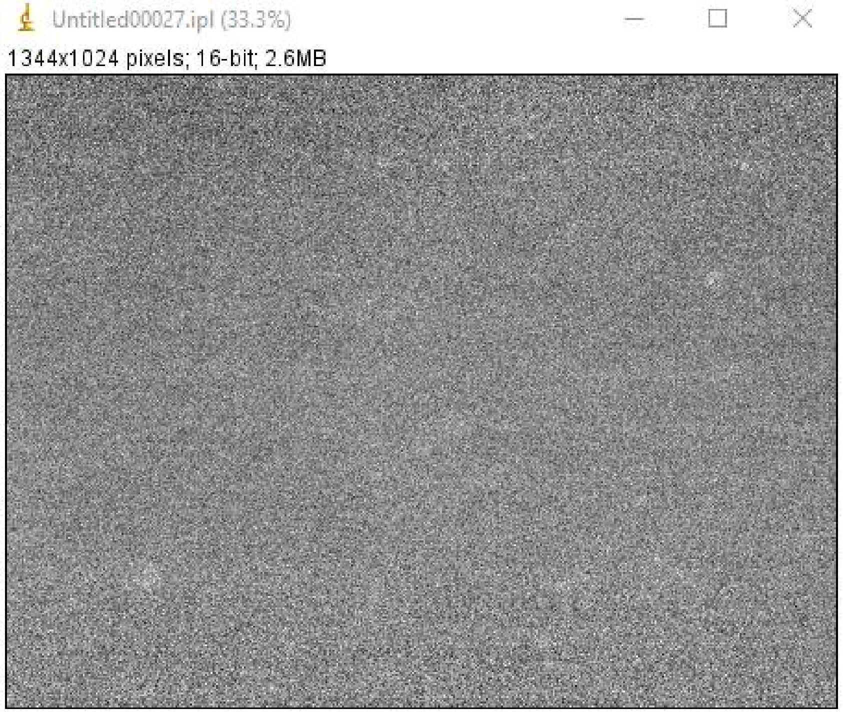

DS fraction - Sample 4

Round marks = 2

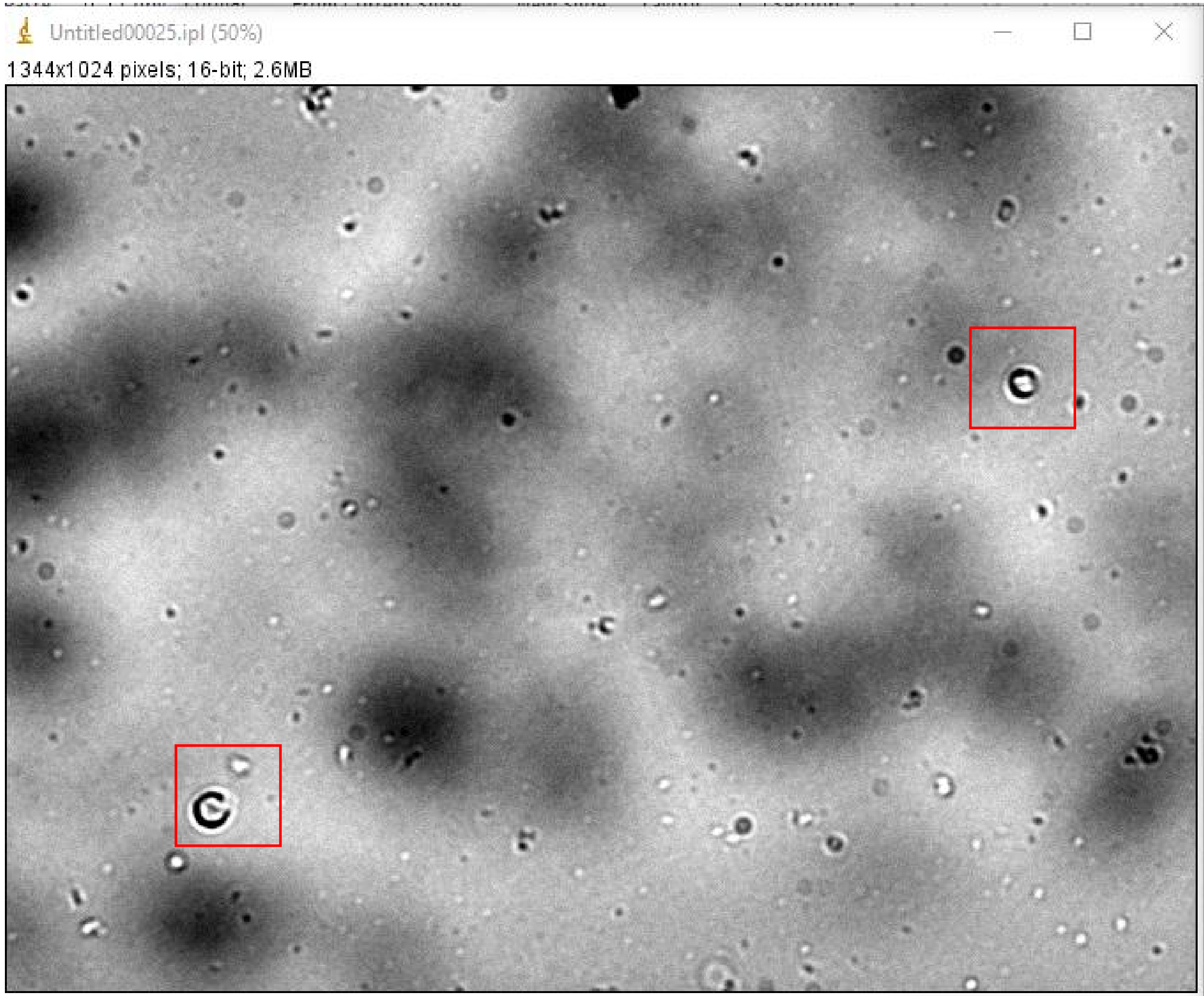

7 marks TxR

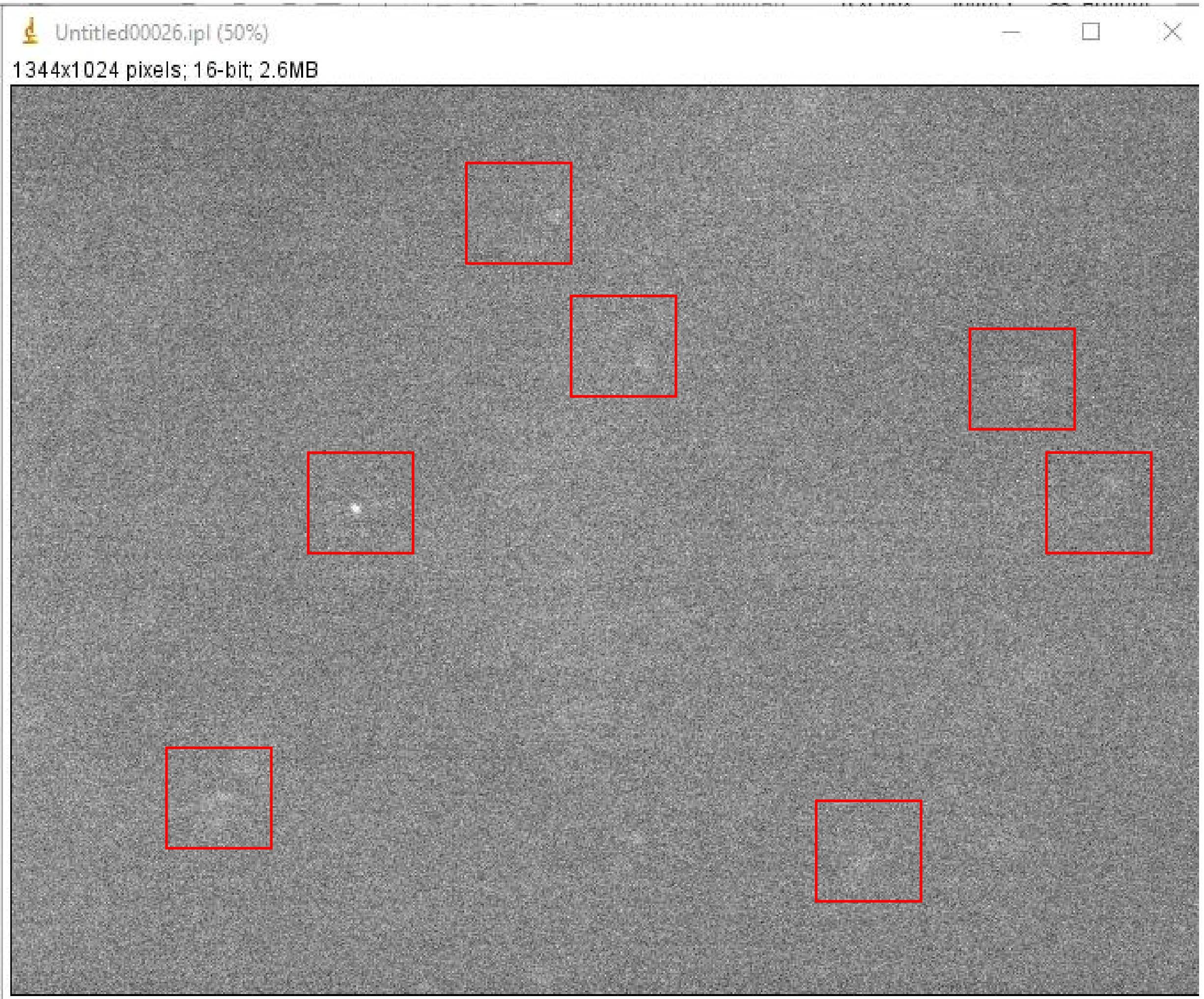

DS fraction - Sample 4

Round marks = 2

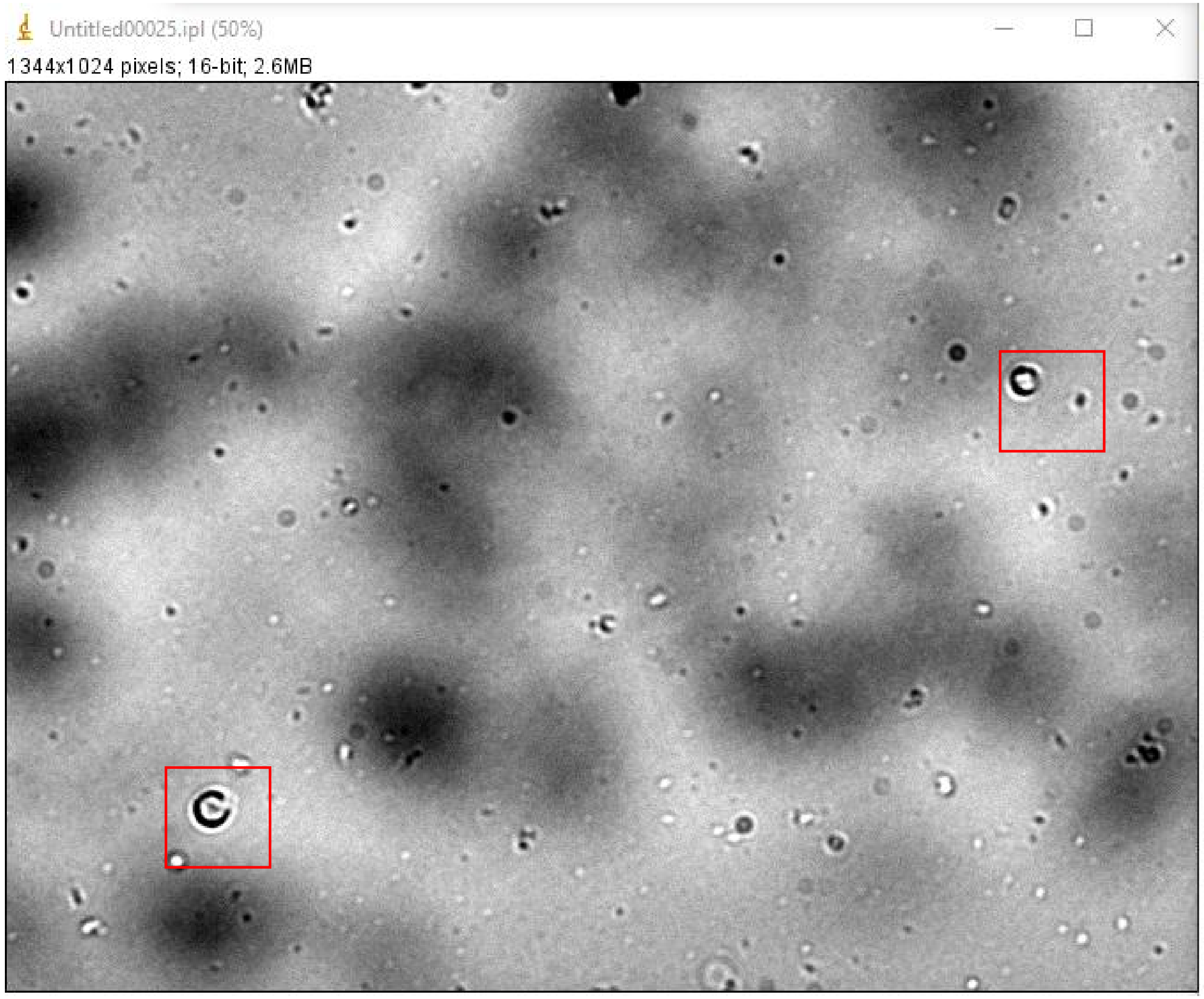

13 marks FITC

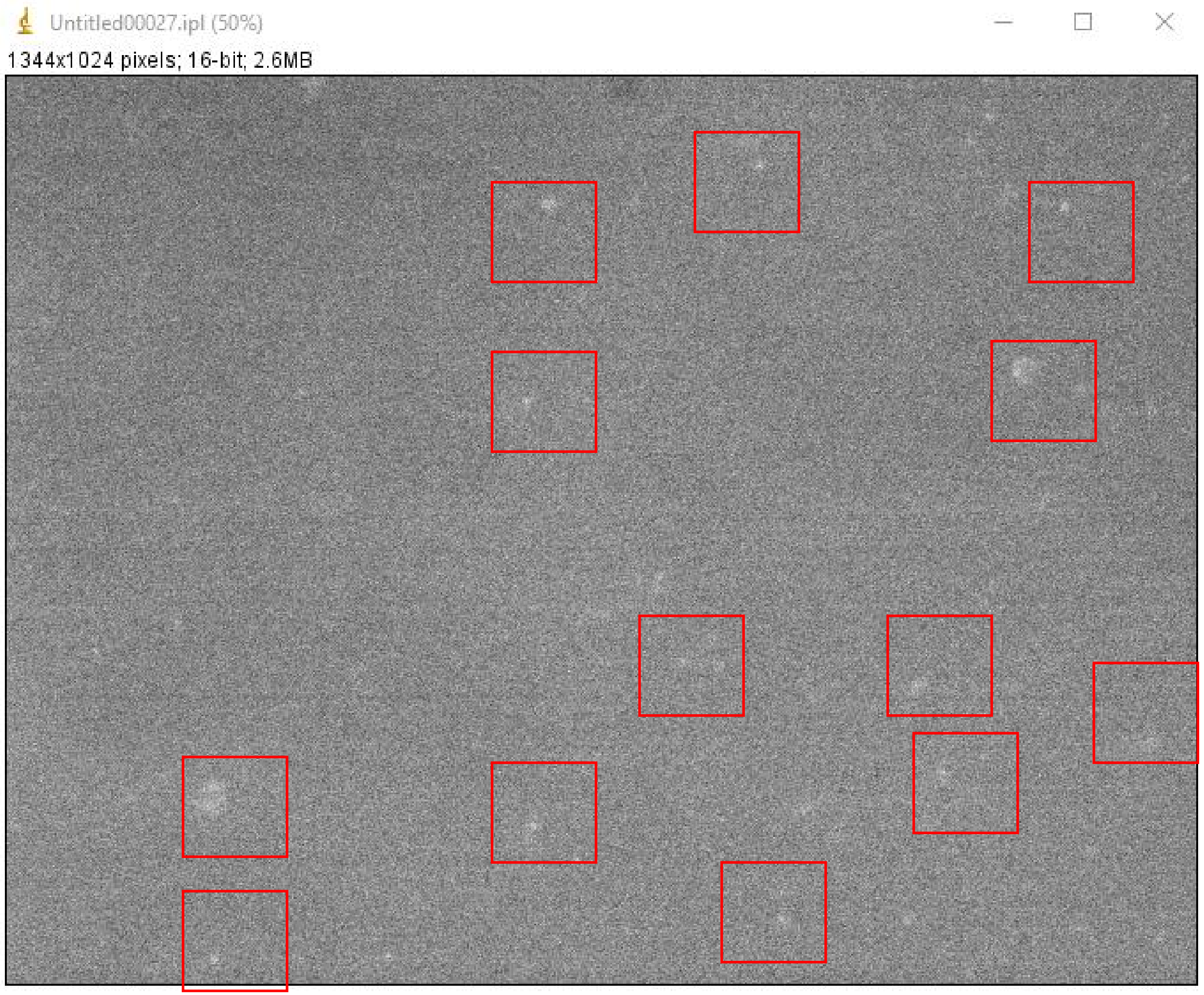

DS fraction - Sample 4

Two marks with the three criteria merge

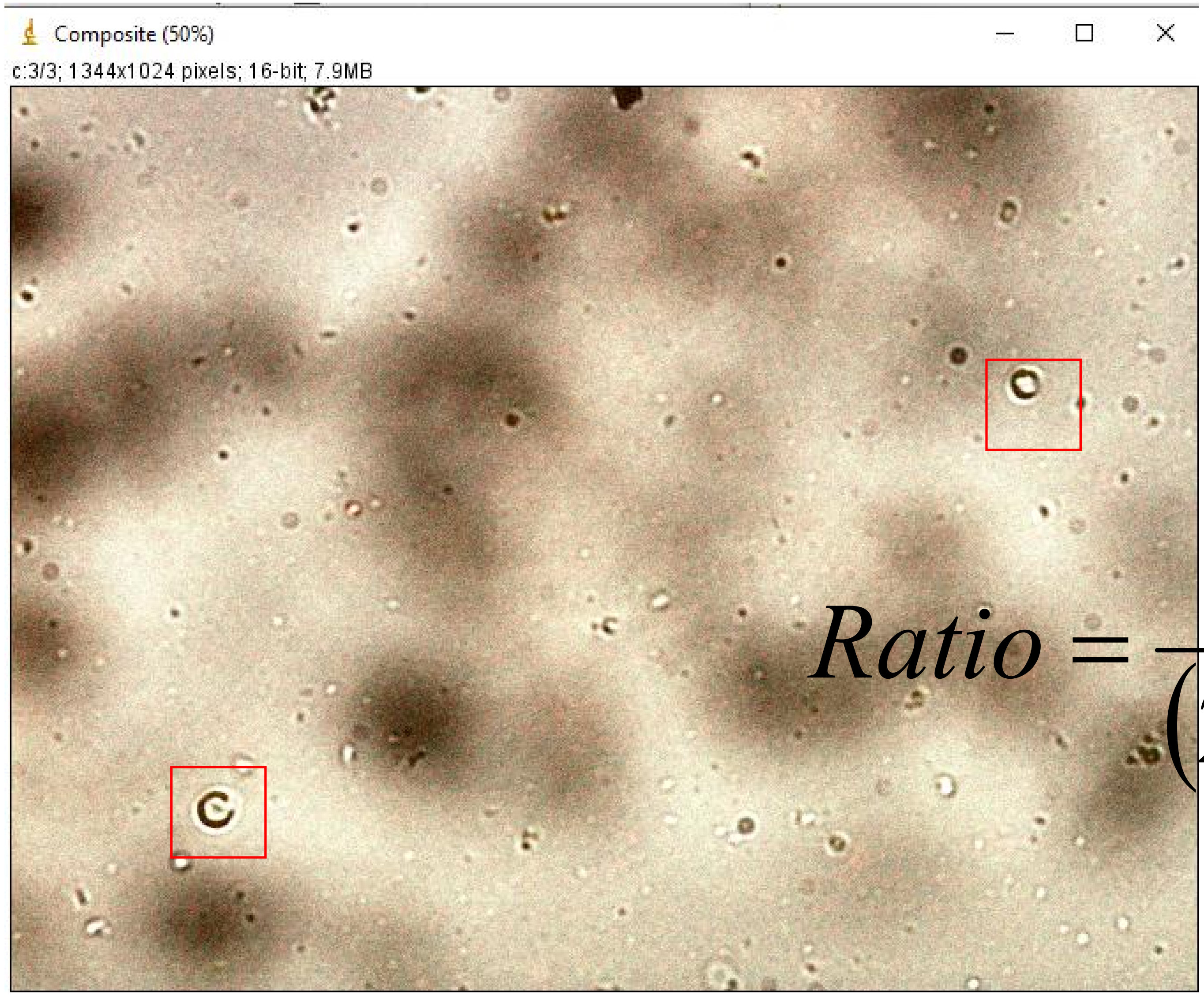

$$Ratio = \frac{2}{(2+7+13)/3} = 0.27$$

Summary Values
Ratios, Mean ± SEM

Whole Supernatant	DS Fraction -Sample 2
0.08<br>0<br>0.188<br>0.125<br><br>**Mean=0.0983**<br>**SE=0.0395**	0.563<br>0.375<br>0.6<br>0.27<br><br>**Mean=0.4520**<br>**SE=0.0781**

Overall Ratio
4.59 (p = 0.007)
